# Replication and single-cycle delivery of SARS-CoV-2 replicons

**DOI:** 10.1126/science.abj8430

**Published:** 2021-10-14

**Authors:** Inna Ricardo-Lax, Joseph M. Luna, Tran Thi Nhu Thao, Jérémie Le Pen, Yingpu Yu, H.-Heinrich Hoffmann, William M. Schneider, Brandon S. Razooky, Javier Fernandez-Martinez, Fabian Schmidt, Yiska Weisblum, Bettina Salome Trüeb, Inês Berenguer Veiga, Kimberly Schmied, Nadine Ebert, Eleftherios Michailidis, Avery Peace, Francisco J. Sánchez-Rivera, Scott W. Lowe, Michael P. Rout, Theodora Hatziioannou, Paul D. Bieniasz, John T. Poirier, Margaret R. MacDonald, Volker Thiel, Charles M. Rice

**Affiliations:** 1 Laboratory of Virology and Infectious Disease, The Rockefeller University, New York, NY 10065, USA.; 2 Institute of Virology and Immunology (IVI), Bern, Switzerland.; 3 Department of Infectious Diseases and Pathobiology, Vetsuisse Faculty, University of Bern, Bern, Switzerland.; 4 Graduate School for Biomedical Science, University of Bern, Bern, Switzerland.; 5 Laboratory of Cellular and Structural Biology, The Rockefeller University, New York, NY 10065, USA.; 6 Laboratory of Retrovirology, The Rockefeller University, New York, NY 10065, USA.; 7 Cancer Biology and Genetics, MSKCC, New York, NY 10065, USA.; 8 Laura and Isaac Perlmutter Cancer Center, New York University Grossman School of Medicine, NYU Langone Health, New York, NY 10016, USA.

## Abstract

Molecular virology tools are critical for basic studies of the severe acute respiratory syndrome coronavirus 2 (SARS-CoV-2) and for developing new therapeutics. Experimental systems that do not rely on viruses capable of spread are needed for potential use in lower-containment settings. In this work, we use a yeast-based reverse genetics system to develop spike-deleted SARS-CoV-2 self-replicating RNAs. These noninfectious self-replicating RNAs, or replicons, can be trans-complemented with viral glycoproteins to generate replicon delivery particles for single-cycle delivery into a range of cell types. This SARS-CoV-2 replicon system represents a convenient and versatile platform for antiviral drug screening, neutralization assays, host factor validation, and viral variant characterization.

## A tool to study SARS-CoV-2

Work with infectious severe acute respiratory syndrome coronavirus 2 (SARS-CoV-2) requires high-level biocontainment facilities, making it important to develop safer molecular tools that can potentially be used under less stringent conditions. Self-replicating RNAs known as replicons have long been used to study pathogenic RNA viruses; however, developing replicons to study SARS-SoV-2 has been challenging because of its large genome. Ricardo-Lax *et al*. used a yeast-based system to construct SARS-CoV-2 replicons that cannot assemble infectious virus because they lack the spike protein required for host cell entry. Transfecting cells with a spike-expressing plasmid and separately with the replicon generates replicon delivery particles (RDPs) that are only capable of one cycle of infection. The replicons and the RDPs can be used in different contexts for drug screening, and viral assays. —VV

## Teaser

SARS-CoV-2 replicon delivery particles enable basic studies and drug development efforts against SARS-CoV-2.

Self-replicating RNAs, known as replicons, are model systems used to genetically probe numerous aspects of RNA virus life cycles without producing infectious virus (*1*–*7*). Replicons for positive-stranded RNA viruses are typically constructed using reverse genetics approaches to replace one or more viral structural proteins with selectable and reporter genes. When translated inside cells, replicon RNA produces viral gene products that establish RNA replication factories, with reporter genes providing readouts for replicon activity and selectable genes permitting selection of cells that stably harbor the replicon. Because key structural components of the virion are missing, replication proceeds without producing infectious virus. Replicon systems that do not require high-containment laboratory settings have been invaluable as molecular virology and high-throughput drug development platforms, as perhaps best exemplified for hepatitis C virus (*8*, *9*).

Reverse genetics systems for severe acute respiratory syndrome coronavirus 2 (SARS-CoV-2), the causative agent of COVID-19, have been developed for fully infectious recombinant virus production (*10*–*13*) and as replicon platforms (*14*–*19*). In the latter case, trans-complementation of the deleted structural gene nucleocapsid (*18*) or envelope and Orf3a genes (*19*) can enable single-cycle–infectious SARS-CoV-2 virion production that may reduce the need for high containment for a range of applications. Although these systems permit spike-dependent replicon delivery, investigation of newly emerging spike variants requires replicon reengineering for each variant. By contrast, SARS-CoV-2 spike-pseudotyped lentiviruses (*20*–*22*) or chimeric rhabdoviruses (*22*, *23*) bearing spike(s) offer a rapid, plasmid-based means of virion production for spike-directed studies (*24*), such as the characterization of neutralizing antibodies. There remains a need for experimental systems that harness the noninfectious advantages of replicons while also enabling studies of virus entry and replication. In principle, combining a spike-deleted SARS-CoV-2 replicon with viral glycoprotein trans-complementation would achieve this goal. Such a system would enable isogenic studies of spike variants in a SARS-CoV-2–based platform while also serving as an appropriately safeguarded infection-based means of replicon launch that could be extended to additional cell types through the use of heterologous viral glycoproteins. Here we describe the construction, activity, and single-cycle virion generation of spike-deleted SARS-CoV-2 replicons and the use of viral glycoprotein trans-complementation to create replicon delivery particles (RDPs).

## Spike-deleted replicon design and optimization of RNA production

**Fig. 1.**
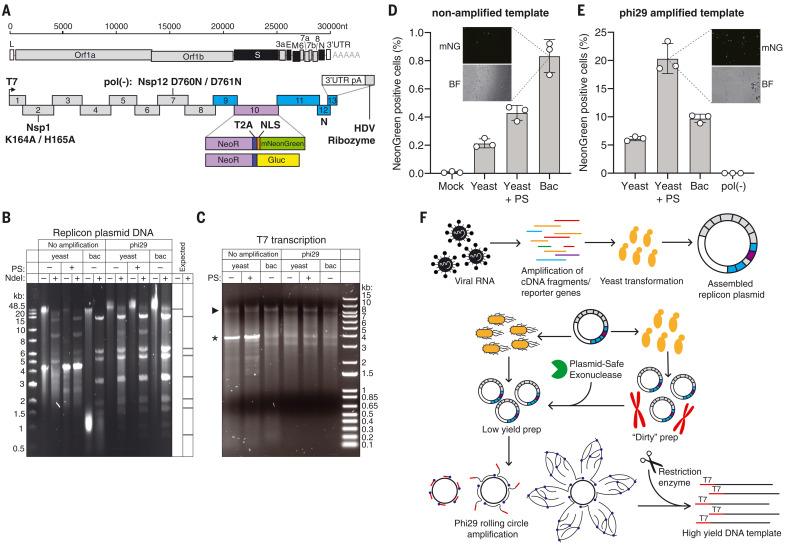
SARS-CoV-2 replicon design and launch optimization. (**A**) (Upper schematic) SARS-CoV-2 genome, with structural proteins in black. (Lower schematic) Replicon amplicon fragments for yeast assembly. Fragments from (*10*) are shown in gray; fragments harboring mutations in Nsp1 or Nsp12 [pol(-)] are marked as such. The reporter gene cassette in place of spike is shown in purple; reengineered flanking regions are in blue. nt, nucleotides; L, leader; UTR, untranslated region; pA: polyA, HDV, hepatitis delta virus; NLS, nuclear localization sequence. (**B**) Agarose gel of replicon DNA recovered from yeast or bacteria (bac). Phi29 amplification or plasmid-safe (PS) DNAse treatment is indicated. Expected NdeI digest is depicted at right. (**C**) Agarose gel of T7 RNA transcription reactions from the DNA plasmids in (B). The arrowhead indicates the expected size of full-length RNA; the asterisk denotes truncated product. (**D** and **E**) Percent of mNeonGreen replicon–positive BHK-21 cells from nonamplified (D) or phi29-amplified (E) DNA templates measured by flow cytometry. Insets show representative mNeonGreen (mNG) and bright-field (BF) images. *N* = 3 biological replicates. Error bars indicate SEM; “Mock” indicates no RNA electroporation. (**F**) Optimized RNA production for SARS-CoV-2 replicons. Overlapping PCR fragments are assembled in yeast and propagated in bacteria or yeast, in which case they are treated with PS DNAse. Subsequent phi29 amplification ensures full-length DNA template availability for transcription.

The SARS-CoV-2 genome is thought to encode 16 nonstructural proteins (Nsp1 to Nsp16) in two overlapping reading frames (Orf1a and Orf1b), as well as four structural proteins [spike (S), membrane (M), envelope (E), and nucleocapsid (N)] and at least seven accessory proteins (3a, 3c, 6, 7a, 7b, 8, and 9b) expressed from subgenomic RNAs or alternative reading frames (Fig. 1A) (*25*, *26*). We adopted a modular design to assemble a replicon that consists of all viral proteins except the primary structural glycoprotein spike (ΔS). The spike transcription-regulating sequence (TRS) was instead used to drive expression of a gene cassette that consists of neomycin-resistance (NeoR) and a reporter gene [nuclear-localized monomeric NeonGreen or secreted *Gaussia* luciferase (Gluc)] separated by a T2A ribosome shift sequence (Fig. 1A). The plasmid encoding the replicon cDNA contains an upstream T7 promoter at the 5′ end for in vitro transcription and a self-cleaving hepatitis delta virus ribozyme at the 3′ end, which cleaves after an encoded polyA sequence yielding an authentic terminus.

For replicon assembly, we employed a recently published RNA virus reverse genetics system in the yeast *Saccharomyces cerevisiae* (*10*). This system leverages transformation-associated recombination to accurately assemble numerous, large overlapping DNA fragments (*27*). Transformation of yeast with equimolar ratios of replicon cDNA fragments led to efficient replicon assembly, as assessed by multiplex polymerase chain reaction (PCR) (fig. S1A). We performed restriction enzyme digests of the resulting DNA to assess plasmid integrity and observed that yeast-derived plasmids were contaminated with yeast genomic DNA and did not reveal the expected NdeI digest pattern (Fig. 1B). In an alternative approach, we propagated yeast-assembled plasmids in bacteria, which boosted plasmid purity, as demonstrated previously (*10*). However, the overall DNA yield was suboptimal in both instances and, in the case of bacterial propagation, often resulted in mutations in the coding region of the viral RNA-dependent RNA polymerase (RdRp). To improve plasmid purity and yield, we developed a method that relies on selective enzymatic digestion of contaminating yeast DNA, followed by preferential amplification of the plasmid product. We first treated plasmid preparations with BamHI, which digests yeast genomic DNA but whose recognition sequence is absent in the replicon plasmid. We subsequently treated the DNA with plasmid-safe (PS) deoxyribonuclease (DNase) to digest linear contaminating yeast DNA. We next turned to multiple-displacement rolling circle amplification using phi29 DNA polymerase (*28*) and random primers to amplify replicon plasmids from either yeast or sequence-verified bacterial clones. Owing to its processivity (>70 kb per binding event), strand-displacement activity, and low error rate (<1 × 10^−6^), phi29 polymerase can exponentially amplify long, circular DNA sequences with high fidelity under isothermal conditions and has been used to replicate bacterial artificial chromosomes, cosmids, mitochondrial DNA, and microbial genomes at the megabase length scale (*29*–*31*). Indeed, amplification of replicon plasmids from yeast or bacteria yielded useful quantities of full-length and intact replicon DNA (Fig. 1B), as verified by amplicon sequencing. We used the resulting DNA for T7 transcription reactions and observed apparently full-length (~27.5 kb) replicon RNA in addition to shorter RNA products (Fig. 1C). To directly compare launch efficiency, mNeonGreen-encoding replicon RNAs were electroporated into BHK-21 cells together with additional in vitro transcribed N mRNA, which has previously been shown to boost launch efficiency (*10*, *15*). The percentage of cells expressing the mNeonGreen reporter was measured 24 hours after electroporation. We observed fewer than 1% of replicon-positive cells using RNA from non–phi29-amplified templates, where bacteria-propagated plasmids yielded the highest (0.8%) mNeonGreen reporter signal (Fig. 1D). By contrast, PS DNase-treated and phi29-amplified templates yielded ~20% mNeonGreen-positive cells. The mNeonGreen signal was dependent on productive replication and transcription, as no signal was observed with the use of replicon RNA that harbors inactivating mutations in Nsp12, the viral RdRp (*32*, *33*) [Fig. 1E; pol(-)]. In summary, the most efficient means of reaching high replicon launch efficiency was achieved by making capped RNA from yeast plasmids that are PS-digested and phi29-amplified before transcription (Fig. 1F).

## Spike-deleted SARS-CoV-2 replicons are convenient and versatile assay platforms

**Fig. 2.**
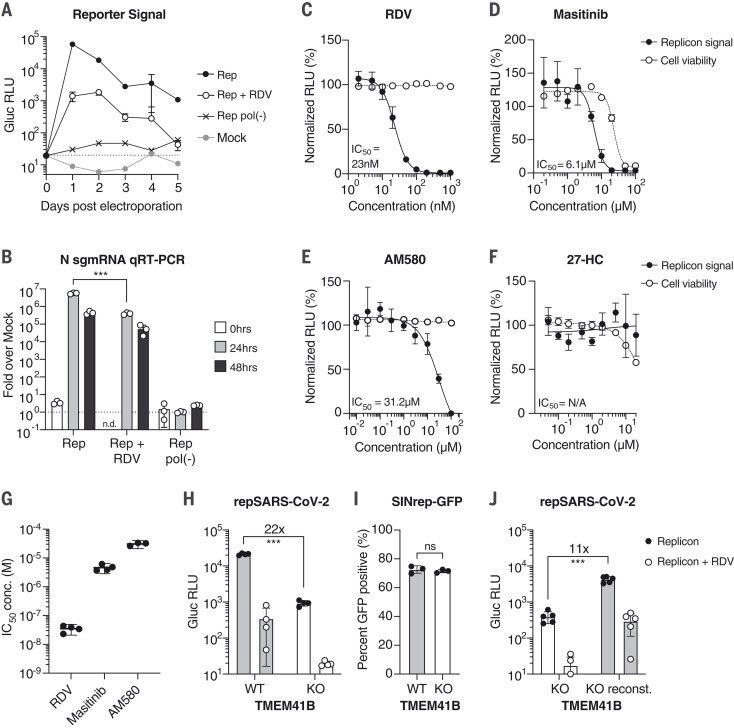
SARS-CoV-2 replicons are sensitive to antiviral compounds, host factor loss, and viral mutant phenotypes. (**A**) *Gaussia* luciferase (Gluc) in Huh-7.5 supernatant from cells electroporated with Gluc replicon RNA (Rep), seeded with 100 nM remdesivir (RDV) or vehicle. Mock electroporation and pol(-) replicons were used as controls. The dashed line indicates the limit of detection. *N* = 4. Error bars indicate SD. RLU, relative light units. (**B**) qRT-PCR measurements for subgenomic N RNA for cells in (A). Signal from mock-infected cells was used for normalization (dashed line). *N* = 3. Error bars indicate SD. ***P < 0.001; n.d., not determined. sgmRNA, subgenomic mRNA. (**C** to **F**) Representative experiments in Huh-7.5 cells were electroporated with the Gluc replicon RNA and seeded with (C) remdesivir (*N* = 4), (D) masitinib (*N* = 4), (E) AM580 (*N* = 3), or (F) 27-hydroxycholesterol (27-HC) (*N* = 3). After 24 hours, Gluc signal in the supernatant (filled circles) and cell viability (empty circles) were measured and normalized to vehicle-treated cells. Error bars indicate SEM. (**G**) IC_50_ values from independent experiments using the compounds presented in (C) to (E). (**H**) Parental Huh-7.5 (WT) and clonal TMEM41B KO cells were electroporated as indicated in (A). Gluc was measured 24 hours after electroporation. *N* = 4. (**I**) Cells as in (H) were electroporated with SINrep-GFP alphavirus replicon RNA. After 24 hours, GFP-positive cells were quantified by flow cytometry. *N* = 3. (**J**) As in (H), using cells reconstituted with TMEM41B. *N* = 5. Error bars indicate SD. ***P < 0.001 (two-sided Student’s *t* test); ns, not significant.

To characterize the utility of spike-deleted replicons for antiviral compound evaluation and screening, host factor validation, and viral mutant phenotype assessment, we constructed a replicon encoding Gluc (Fig. 1A). RNA transcripts were electroporated into Huh-7.5 cells, and cumulative reporter activity per 24-hour period was monitored via luciferase quantification from culture supernatant. By 24 hours after electroporation, we detected robust luciferase activity, which steadily decreased over time (Fig. 2A). Luciferase activity was dependent on viral replication, as no signal was observed for the pol(-) replicon mutant. Replicon-driven luciferase expression was also sensitive to remdesivir, a well-characterized inhibitor of the SARS-CoV-2 RdRp (*34*). As a luciferase-independent measure of replicon activity, we measured N subgenomic mRNA levels by quantitative reverse transcription (qRT)–PCR and observed RNA accumulation kinetics that paralleled reporter gene expression (Fig. 2B). This signal was distinct from that of coelectroporated N mRNA, which is needed for efficient replicon or virus launch from RNA (*10*) and was detectable in all measured samples as expected (fig. S1B). These results show that SARS-CoV-2 replicons undergo robust and quantifiable RNA replication.

Drug screening platforms are among the most fruitful applications for replicons. As a proof of concept, we treated cells bearing luciferase reporter replicons with compounds reported to have direct-acting antiviral (DAA) activity at different stages of the viral life cycle: the SARS-CoV-2 RdRp inhibitor remdesivir (*34*), and masitinib, a proposed 3C-like protease inhibitor (*35*). As a negative control, we used 27-hydroxycholesterol (27-HC), a compound reported to act against both SARS-CoV-2 and human coronavirus OC43 (HCoV-OC43) by affecting viral entry, which we bypassed by means of RNA transfection (*36*). We also tested the host-targeting agent (HTA) AM580, a retinoid derivative reported to have broad antiviral activity by disrupting sterol regulatory element–binding protein (SREBP) lipid signaling (*37*). For remdesivir, we observed median inhibitory concentration (IC_50_) values and low cytotoxicity profiles similar to those previously reported for live virus (Fig. 2C) (*34*). For masitinib, we observed inhibition with IC_50_ values similar to what was shown with infectious HCoV-OC43 and SARS-CoV-2 (*35*). However, cellular toxicity was observed at high concentrations (Fig. 2D). For AM580, IC_50_ values were consistent with reported results, with low cytotoxicity at inhibitory concentrations (Fig. 2E) (*37*). As expected, 27-HC showed no detectable inhibition (Fig. 2F). These representative results showcase the utility of SARS-CoV-2 replicons as scalable drug discovery platforms focused on intracellular viral replication events (Fig. 2G).

We next evaluated whether replicons could be used to identify or validate intracellular SARS-CoV-2 host factors. Transmembrane protein 41B (TMEM41B) was recently reported to be a critical intracellular host factor for multiple coronaviruses (*38*). To test whether SARS-CoV-2 replicon activity depended on TMEM41B function, we electroporated wild-type (WT) and TMEM41B knockout (KO) Huh-7.5 cells with reporter replicon RNA. Consistent with results obtained with virus, TMEM41B KO resulted in a 22-fold decrease in reporter activity compared with that for WT Huh-7.5 cells (Fig. 2H)—an effect similar in magnitude to that of remdesivir treatment. By contrast, an alphavirus replicon was not affected by TMEM41B ablation (Fig. 2I). TMEM41B reconstitution in KO cells led to an 11-fold increase in replicon activity (Fig. 2J). These results demonstrate that SARS-CoV-2 replicons are sensitive to disruption of critical intracellular host factors.

## SARS-CoV-2 replicons reveal viral determinants of interferon sensitivity

**Fig. 3.**
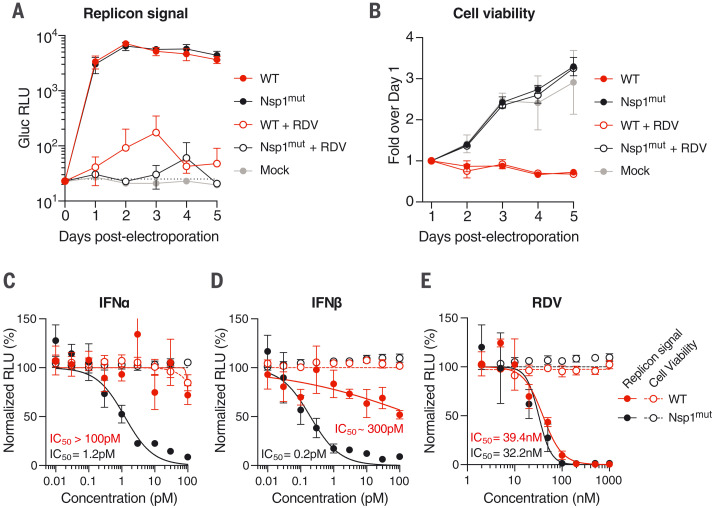
Nsp1-deficient replicons are hypersensitive to interferons. (**A** and **B**) Time course measurements of Gluc in the supernatant (A) or cell viability (B) of Huh-7.5 cells electroporated with WT or Nsp1 K164A/H165A double mutant (Nsp1^mut^) Gluc replicon RNA. Cells were seeded with 100 nM remdesivir or vehicle and were washed in phosphate-buffered saline 24 hours before each respective time-point collection. Mock-electroporated cells were used as controls for post-electroporation cell viability. The dashed line indicates the limit of detection. *N* = 4. Error bars indicate SD. (**C** to **E**) Huh-7.5 cells were electroporated with WT or Nsp1^mut^ replicon RNA and seeded on 96-well plates containing the indicated concentrations of (C) IFNα, (D) IFNβ, or (E) remdesivir. Gluc activity (filled circles) and cell viability (empty circles) were measured 24 hours after electroporation. *N* = 4. Error bars indicate SD.

SARS-CoV-2 replicons can also be used to characterize viral mutants. Recent studies have highlighted the importance of Nsp1 as a suppressor of host translation (*39*–*44*), consistent with prior studies of SARS-CoV (*45*, *46*). This Nsp1 activity can be ablated by two amino acid substitutions at positions 164 and 165 (*47*) that are important for association with the 40*S* ribosomal subunit (*39*, *40*). Nsp1 mutant (Nsp1^mut^) replicons might preserve translation and cell viability and hence prolong replicon activity—or, given the proposed role of Nsp1 in blunting the innate immune response (*14*, *39*, *48*), such mutations might attenuate the replicon. We generated Nsp1^mut^ replicons and found that they performed similarly to WT versions in Huh-7.5 cells and could be inhibited by remdesivir (Fig. 3A) but exhibited less cellular toxicity (Fig. 3B). By contrast, viability of cells harboring WT replicons remained low even when replicons were launched in the presence of remdesivir (Fig. 3B). Taken together, these results suggest that the initial burst of SARS-CoV-2 Nsp1, expressed from transfected replicon RNA, is an important mediator of cell toxicity.

Because SARS-CoV-2 Nsp1 activity is proposed to halt production of interferon-stimulated gene (ISG) products (*39*, *40*), we hypothesized that Nsp1^mut^ replicons would be more sensitive to interferons. Indeed, upon launch in Huh-7.5 cells, Nsp1 mutants were hypersensitive to interferon-α (IFNα) and interferon-β (IFNβ) compared with WT replicons (Fig. 3, C and D), whereas both replicons exhibited similar sensitivity to remdesivir (Fig. 3E). These results highlight the ability of Nsp1 to blunt the antiviral ISG response by inhibiting host translation, which contributes to cytotoxicity. Although these features of Nsp1^mut^ replicons may be advantageous for DAA and HTA screening, the lack of Nsp1 functions may affect screening outcomes in different cell backgrounds, depending on innate immune competence or other factors. Further, attempts to leverage the lower cytotoxicity of Nsp1^mut^ replicons to select for stable cell lines that harbor noncytopathic replicons, as done previously with HCoV-229E (*49*), have thus far been unsuccessful in Huh-7.5 and BHK-21 cells. Different cell backgrounds and multiple adaptive mutations may be necessary to achieve this goal.

## SARS-CoV-2 replicons can be trans-complemented with spike to generate single-cycle virions

**Fig. 4.**
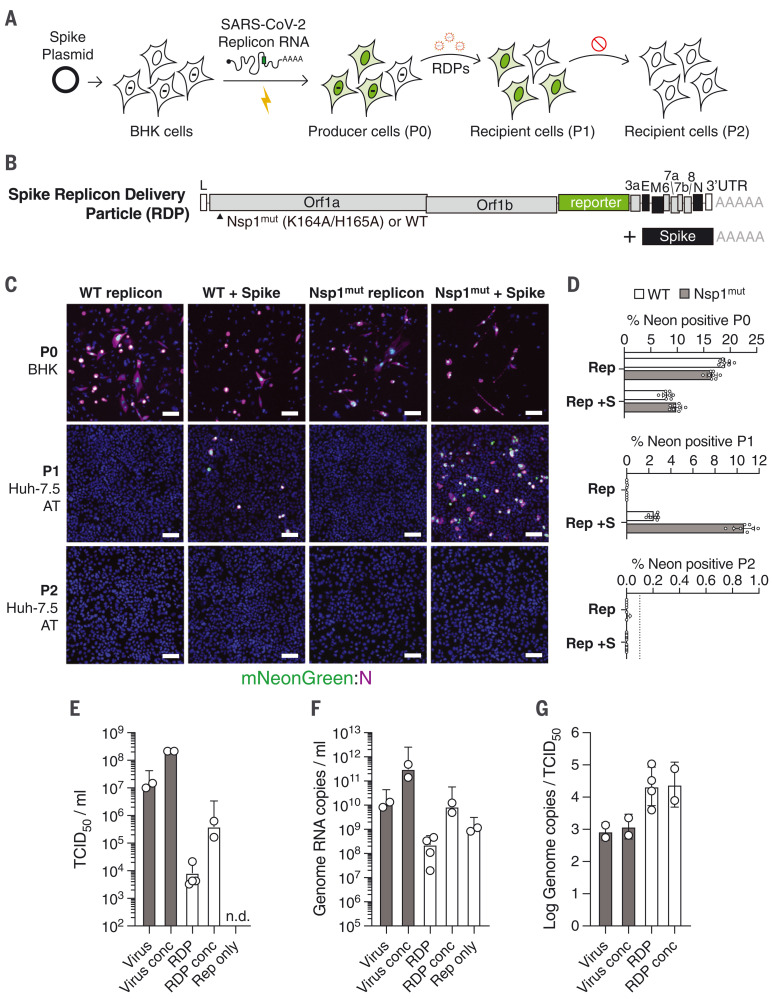
Trans-complementation of replicons with spike yields single-cycle SARS-CoV-2. (**A**) A scheme to trans-complement replicons with ectopically expressed spike for single-cycle virion production. BHK-21 cells are transfected with a spike-encoding plasmid; 24 hours later, they are electroporated with ΔS mNeonGreen SARS-CoV-2 replicon RNA. Supernatant from these producer cells (P0) is collected and passed onto naïve recipient cells (P1), yielding reporter activity. A second round of passaging onto naïve recipient cells (P2) fails to propagate the replicon. (**B**) A spike trans-complemented replicon consists of spike-deleted replicon RNA alongside plasmid-driven spike expression. Nsp1 mutations relative to the WT sequence are indicated. (**C**) BHK-21 producer cells (P0) alone or transfected with a spike-encoding plasmid were electroporated with WT or Nsp1^mut^ replicon RNAs. The RDPs in resulting supernatants were concentrated after 24 hours and passaged onto Huh-7.5 cells that overexpress ACE2 and TMPRSS2 (Huh-7.5 AT cells; P1 and P2), as in (A). Immunofluorescence images at 4× magnification of the mNeonGreen signal (green) and N antibody staining (magenta) are shown. Scale bars, 100 μm. (**D**) Quantification of the percentage of NeonGreen-positive cells in each passage for the results in (C). The dashed line denotes the lower limit of quantification. *N* = 8. Error bars indicate SD. (**E**) TCID_50_ per milliliter of independently prepared SARS-CoV2 and RDP stocks were calculated by end-point dilution assay on Huh-7.5 AT cells. Conc, stocks concentrated by PEG precipitation; n.d., not detected. (**F**) Genome RNA copies per milliliter from the virus and RDP stocks indicated in (E) were calculated by qRT-PCR. (**G**) The ratio between RNA copies per milliliter indicated in (F) and TCID_50_ per milliliter indicated in (E) was calculated to reflect specific infectivity. In (E) to (G), error bars indicate SD.

Although BHK-21 and Huh-7.5 cells are permissive for replicon launch via electroporation, physiologically relevant lung cell lines and primary cell types are more challenging to transfect. Such cells also have intact RNA sensing and innate immune functions, which BHK-21 and Huh-7.5 cells lack (*50*, *51*). Electroporation or other methods of RNA delivery also preclude the study of normal viral entry processes, and the large quantities of transfected RNA do not mimic virus infection, in which one or few RNA genomes initiate productive replication. As a more authentic route for replicon delivery, we attempted to package replicons as single-cycle virions that could infect cells in a spike-dependent manner but would not produce infectious progeny capable of further spread. We refer to these single-cycle virions as RDPs (replicon delivery particles). We transfected BHK-21 cells with a spike-expressing plasmid; 24 hours later, we coelectroporated spike-deleted mNeonGreen replicon RNA and N mRNA (Fig. 4, A and B). The full-length native spike gene was optimized for human codons and designed to lack 5′ untranslated region (5′UTR), leader, TRS, and 3′UTR viral sequences to minimize the possibility of recombination into the replicon (*52*, *53*). Given that Nsp1 hindered cell survival after electroporation, we hypothesized that Nsp1^mut^ replicons could be trans-complemented more efficiently than WT replicons. To test this, WT or Nsp1^mut^ replicons were electroporated into spike-expressing cells. After 24 hours, the putative RDPs were concentrated from the supernatant of producer cells by polyethylene glycol (PEG) precipitation and introduced to the culture medium of recipient Huh-7.5 cells overexpressing ACE2 and TMPRSS2 (Huh-7.5 AT). We detected mNeonGreen-positive cells in a spike-dependent manner (Fig. 4, C and D; P1) and found that the proportion of positive cells from Nsp1^mut^ RDPs was five times that from WT replicons. No mNeonGreen-positive cells were detected upon further passage of the supernatant from RDP-positive Huh-7.5 AT cells (P1) onto naïve Huh-7.5 AT cells (P2) (Fig. 4, C and D). This suggests that spike RDPs exhibit single-cycle infectivity. Consistent with this observation, spike expression was readily detected in the producer cells (P0) but absent in RDP-infected P1 cells (fig. S2, A and B). N protein expression was observed in producer and P1 cells, but not in P2 cells as expected (fig. S2C). Further, consistent with single-cycle infectivity, spike RDP infection did not yield characteristic syncytia indicative of spike expression, as is typically seen with live virus (fig. S2, D and E). We observed spike RDP titers of 10^3^ to 10^4^ median tissue culture infectious doses per milliliter (TCID_50_/ml) in Huh-7.5 AT cells using unconcentrated stocks and up to 6.2 × 10^5^ TCID_50_/ml upon concentration, with specific infectivity about 20-fold lower than that of virus (Fig. 4, E to G). This may indicate that RDPs have reduced spike density compared with virus.

## Neutralization assays with RDPs recapitulate authentic SARS-CoV-2 antibody phenotypes

**Fig. 5.**
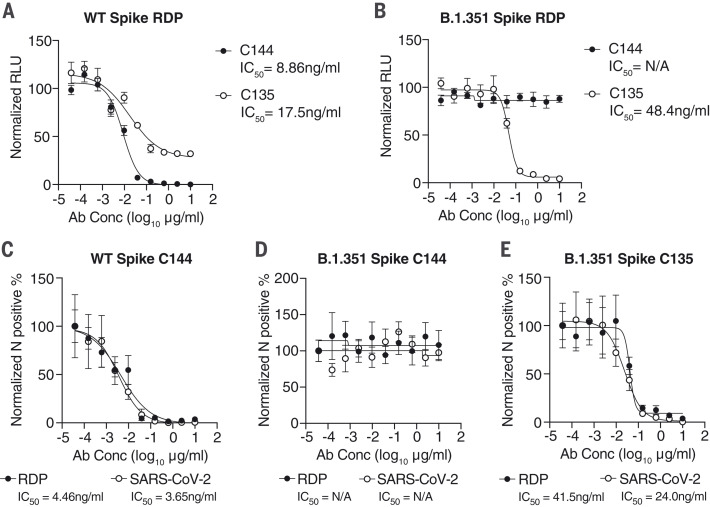
Neutralization assays with RDPs recapitulate authentic SARS-CoV-2 antibody phenotypes. (**A** and **B**) Antibody neutralization assays in Huh-7.5 cells of Gluc RDPs trans-complemented with WA1/2020 spike (A) or the B.1.351 South African variant (B) in the presence of increasing concentrations of C144 and C135 neutralizing antibodies. Data are representative of two independent experiments and are normalized to infected cells without antibody. *N* = 3. Error bars indicate SEM. Ab Conc, antibody concentration; IC_50_, concentration resulting in 50% reduction in relative RLU values; N/A, not applicable. (**C** to **E**) Neutralization assays for SARS-CoV-2 RDPs or virus in the presence of increasing antibody concentrations. WT spike with C144 antibody (C), B.1.351 spike with C144 antibody (D), and B.1.351 with C135 antibody (E) are shown, representative of two independent experiments. Data are normalized to infected cells without antibody. *N* = 3. Error bars indicate SEM.

Spike RDPs could complement pseudovirus assays based on HIV-1 or vesicular stomatitis virus (VSV), with a SARS-CoV-2–based, and therefore more authentic, single-cycle alternative. We generated Nsp1^mut^ Gluc RDPs trans-packaged with either the prototype spike (WA1/2020 isolate) or the B.1.351 (Beta) variant of concern (*54*). We performed neutralization assays with these RDPs using two well-characterized human monoclonal antibodies—C135 and C144—capable of potently neutralizing SARS-CoV-2 or pseudoviruses expressing the prototype spike (*55*). C144 binding and neutralization is ablated by the spike Glu^484^→Lys (E484K) mutation, which was originally identified by antibody selection experiments using a VSV pseudovirus (*56*) and which later appeared in the B.1.351 variant (*54*). RDPs harboring the prototype spike were neutralized with both antibodies, whereas only C135 neutralized B.1.351 spike RDPs (Fig. 5, A and B). Neutralization curves and relative IC_50_ values were comparable to those obtained with the respective SARS-CoV-2 isolates (Fig. 5, D to F) and to the findings of previous studies that used pseudovirus assays (*55*, *56*), with the added advantage that RDP generation does not require deletions in the spike coding sequence (*22*).

## Spike-deleted SARS-CoV-2 replicons can incorporate VSV glycoprotein

**Fig. 6.**
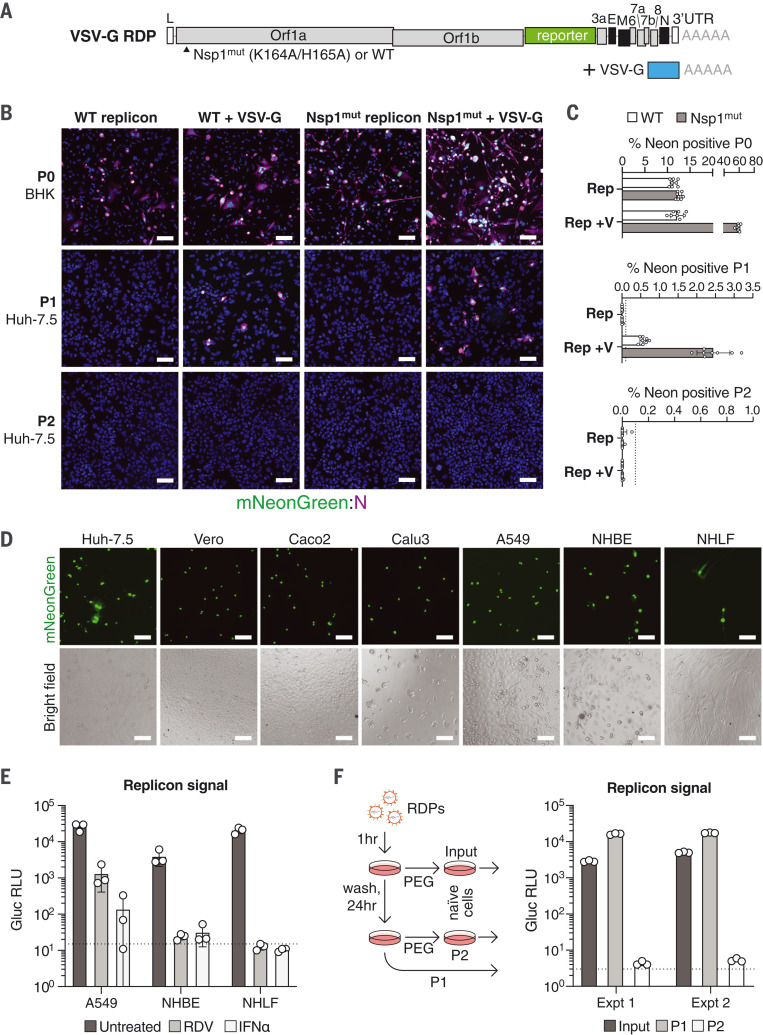
VSV-G pseudotyping for efficient SARS-CoV-2 replicon delivery. (**A**) Schematic of the elements required for production of VSV-G RDPs. (**B**) BHK-21 producer cells (P0) alone or transfected with VSV-G were electroporated with WT or Nsp1^mut^ replicons. After 48 hours, RDPs in supernatants were serially passaged onto Huh-7.5 cells (P1 and P2). mNeonGreen signal and N antibody staining is depicted at 4× magnification. Scale bars, 100 μm. (**C**) Percentage of mNeonGreen-positive cells in each passage from (B). *N* = 8. Error bars indicate SD; dashed lines indicate the lower limit of quantification. (**D**) Indicated cell lines were incubated with mNeonGreen VSV-G RDPs for 24 hours. Bright-field and fluorescent images were taken at 10× magnification. Scale bars, 100 μm. (**E**) NHBE, NHLF, and A549 cells were pretreated for 24 hours with 100 nM remdesivir or 100 pM IFNα and infected with Gluc VSV-G RDPs. Gluc activity was measured 24 hours after infection. *N* = 3. Error bars indicate SD; the dashed line represents the limit of detection. (**F**) Single-cycle infectivity of VSV-G RDPs. Supernatant from RDP-infected cells (P1) was read for Gluc activity and passaged onto naive cells (P2) after PEG concentration. Input supernatant serves as a positive control for concentrated input. Schema are shown at left and results at right for two experiments. The dashed line represents the limit of detection.

Efficient entry with spike RDPs appears to require high levels of ACE2 and TMPRSS2 in Huh-7.5 cells because RDP addition to Huh-7.5 or Vero cells not overexpressing these factors was unproductive. Because numerous cell lines relevant for SARS-CoV-2 studies may have insufficient ACE2 or TMPRSS2 levels unless engineered to support viral entry (*57*), we tested whether spike-deleted replicons could be packaged with VSV glycoprotein (VSV-G), analogous to one-way lentiviral transduction. In such systems, VSV-G pseudotyping provides an efficient means of lentiviral vector entry (*58*) and, for RDPs, might provide an ACE2-independent means of replicon delivery. We transfected BHK-21 cells with a VSV-G expression plasmid followed by coelectroporation with spike-deleted mNeonGreen replicon RNA and N mRNA (Fig. 6A). The resulting RDPs in the supernatant were concentrated and added to naïve Huh-7.5 cells. After 24 hours, we observed mNeonGreen-positive cells in a VSV-G–dependent manner (Fig. 6, B and C; P1). Notably, there was no measurable signal in a second passage (Fig. 6, B and C; P2) and no VSV-G carryover was detected in the P1 cells (fig. S3, A and B), which suggests that VSV-G RDPs have single-cycle infectivity. In contrast to spike RDPs, producer (P0) VSV-G–expressing BHK-21 cells had a significantly higher ratio of mNeonGreen than did control cells when the Nsp1^mut^ replicon was used, which is suggestive of local VSV-G–dependent spread. P1 cells also exhibited higher mNeonGreen and N protein positivity using Nsp1^mut^ RDPs than did WT RDPs (Fig. 6, B and C, and fig. S3C). We observed VSV-G RDP titers between 1 × 10^3^ and 8 × 10^3^ TCID_50_/ml on Huh-7.5 cells. We then tested VSV-G RDP infectivity on additional cell types: African green monkey VeroE6 cells, human Caco2 intestinal epithelial cells (*59*, *60*), and both Calu3 and A549 human lung adenocarcinoma cells (*61*–*64*). Additionally, we tested RDP activity in two lines of human airway cells: normal human bronchial (or tracheal) epithelial (NHBE) cells and normal human lung fibroblast (NHLF) cells (Fig. 6D). All cells were susceptible to infection with VSV-G RDPs and exhibited productive viral replication, as evident from mNeonGreen reporter expression. We pretreated NHBE, NHLF, and A549 cells with 100 nM remdesivir or 100 pM IFNα and subsequently added Gluc VSV-G RDPs. As shown in Fig. 6E, both treatments inhibited Gluc reporter activity to near-baseline levels. Overall, these data demonstrate that VSV-G RDPs provide a flexible means of replicon launch in primary cell contexts.

To further examine whether VSV-G RDPs have single-cycle infectivity using a more sensitive approach, we generated Gluc-expressing VSV-G RDPs and infected naïve Huh-7.5 cells followed by Gluc measurements 24 hours later (P1). Whereas the VSV-G RDP–positive cells produced a robust Gluc signal, no signal was observed when the supernatant from P1 was concentrated and passaged onto naïve cells (P2) (Fig. 6F). Input supernatant was similarly concentrated and used as positive control for VSV-G RDP concentration and single-cycle infection.

## VSV-G trans-complementation of a SARS-CoV-2 replicon lacking all accessory genes

Because our RDP results to this point had relied on spike-deleted replicons with or without mutations in Nsp1, we next examined whether replicons harboring additional deletions could be packaged into RDPs. Previous work with SARS-CoV and SARS-CoV-2 has shown that M and E genes are required for virion morphogenesis and release (*65*, *66*). On that basis, we tested VSV-G packaging of a replicon that lacked all accessory genes but retained genes encoding the structural M, E, and N proteins (ΔAcc) (fig. S4A). VSV-G–dependent infectivity was observed on recipient Huh-7.5 cells when both mNeonGreen and Gluc ∆Acc versions were used at levels comparable to that of the spike-deleted (∆S) replicon (fig. S4, B to D). Thus, accessory genes are dispensable for replicon pseudotyping with VSV-G.

In this paper, we highlight several features of SARS-CoV-2 RDPs. First, RDPs enable a rapid and isogenic means of generating single-cycle SARS-CoV-2 virions for spike-directed efforts, such as screening spike variants against neutralizing antibodies while eliminating the potential for spike mutations to arise, as is commonly encountered with cell culture–passaged virus (*67*, *68*) and for screening antibodies against additional structural proteins. Second, single-cycle infectivity can be achieved with VSV-G, which may be beneficial for studies in systems where ACE2 receptor overexpression is infeasible. Finally, in contrast to replicon launch by RNA transfection, RDPs can be frozen, stored, and distributed because they require no specialized equipment to use once generated. These attributes could eventually enable high-throughput drug screening without the need for high-containment settings. Overall, these features highlight how SARS-CoV-2 spike-deleted replicons and RDPs provide a flexible and single-cycle–infectious platform for future studies of this pandemic virus.

## Supplementary Material

20211014-1

## Supplementary Materials

### This PDF file includes:

Materials and Methods Figs. S1 to S4 References (*69*–*73*)

### Other Supplementary Material for this manuscript includes the following:

Table S1

MDAR Reproducibility Checklist

## Notes

View/request a protocol for this paper from *Bio-protocol*.
