# Supplementary material for: Replication and single-cycle delivery of SARS-CoV-2 replicons
Source: Science. 2021 Oct 14;374(6571):1099–106. doi: 10.1126/science.abj8430 (PMC9007107; doi:10.1126/science.abj8430)
Supplement: 20211014-1 [file science.abj8430.v1.pdf]

Cite as: I. Ricardo-Lax *et al.*, *Science* 10.1126/science.abj8430 (2021).

Replication and single-cycle delivery of SARS-CoV-2 replicons

Inna Ricardo-Lax[1]†, Joseph M. Luna[1]†, Tran Thi Nhu Thao[2,3,4], Jérémie Le Pen[1], Yingpu Yu[1], H.-Heinrich Hoffmann[1], William M. Schneider[1], Brandon S. Razooky[1], Javier Fernandez-Martinez[5], Fabian Schmidt[6], Yiska Weisblum[6], Bettina Salome Trüeb[2,3], Inês Berenguer Veiga[2,3], Kimberly Schmied[2,3], Nadine Ebert[2,3], Eleftherios Michailidis[1], Avery Peace[1], Francisco J. Sánchez-Rivera[7], Scott W. Lowe[7], Michael P. Rout[5], Theodora Hatziioannou[6], Paul D. Bieniasz[6], John T. Poirier[8], Margaret R. MacDonald[1], Volker Thiel[2,3]*, Charles M. Rice[1]*

[1]Laboratory of Virology and Infectious Disease, The Rockefeller University, New York, NY 10065, USA. [2]Institute of Virology and Immunology (IVI), Bern, Switzerland. [3]Department of Infectious Diseases and Pathobiology, Vetsuisse Faculty, University of Bern, Bern, Switzerland. [4]Graduate School for Biomedical Science, University of Bern, Bern, Switzerland. [5]Laboratory of Cellular and Structural Biology, The Rockefeller University, New York, NY 10065, USA. [6]Laboratory of Retrovirology, The Rockefeller University, New York, NY 10065, USA. [7]Cancer Biology and Genetics, MSKCC, New York, NY 10065, USA. [8]Laura and Isaac Perlmutter Cancer Center, New York University Grossman School of Medicine, NYU Langone Health, New York, NY 10016, USA.

†These authors contributed equally to this work.

***Corresponding author. Email: volker.thiel@vetsuisse.unibe.ch (V.T.); ricec@rockefeller.edu (C.M.R.)**

Molecular virology tools are critical for basic studies of the Severe Acute Respiratory Syndrome Coronavirus 2 (SARS-CoV-2) and for developing new therapeutics. There remains a need for experimental systems that do not rely on viruses capable of spread that could potentially be used in lower containment settings. Here, we develop spike-deleted SARS-CoV-2 self-replicating RNAs using a yeast-based reverse genetics system. These non-infectious self-replicating RNAs, or replicons, can be trans-complemented with viral glycoproteins to generate Replicon Delivery Particles (RDPs) for single-cycle delivery into a range of cell types. This SARS-CoV-2 replicon system represents a convenient and versatile platform for antiviral drug screening, neutralization assays, host factor validation, and characterizing viral variants.

Self-replicating RNAs, known as replicons, are model systems used to genetically probe numerous aspects of RNA virus life cycles without producing infectious virus (*1*–*7*). Replicons for positive-stranded RNA viruses are typically constructed using reverse genetics approaches to replace one or more viral structural proteins with selectable and reporter genes. When translated inside cells, replicon RNA produces viral gene products that establish RNA replication factories, with reporter genes providing readouts for replicon activity and selectable genes permitting selection of cells that stably harbor the replicon. Because key structural components of the virion are missing, replication proceeds without producing infectious virus. Replicon systems that do not require high containment laboratory settings have been invaluable as molecular virology and high-throughput drug development platforms, as perhaps best exemplified for hepatitis C virus (*8*, *9*).

Reverse genetics systems for SARS-CoV-2, the causative agent of COVID-19, have been developed for fully-infectious recombinant virus production (*10*–*13*) and as replicon platforms (*14*–*19*). In the latter case, *trans*-complementation of the deleted structural gene nucleocapsid (N) (*18*) or envelope (E) and Orf3a genes (*19*) can enable single-cycle infectious SARS-CoV-2 virion production that may reduce the need for high containment for a range of applications. While these systems permit spike-dependent replicon delivery, interrogating newly emerging spike variants requires replicon reengineering for each variant. In contrast, SARS-CoV-2 spike-pseudotyped lentiviruses (*20*–*22*) or chimeric rhabdoviruses (*22*, *23*) bearing spike(s) offer a rapid, plasmid-based means of virion production for spike directed studies (*24*), such as the characterization of neutralizing antibodies. There remains a need for experimental systems that harness the non-infectious advantages of replicons, while also enabling studies of virus entry and replication. In principle, combining a spike-deleted SARS-CoV-2 replicon with viral glycoprotein *trans*-complementation would achieve this goal. Such a system would enable isogenic studies of spike variants in a SARS-CoV-2 based platform, while also serving as an appropriately safe-guarded infection-based means of replicon launch that may be extended to additional cell types by using heterologous viral glycoproteins. Here we describe the construction, activity and single-cycle virion generation of spike-deleted SARS-CoV-2 replicons using viral glycoprotein *trans*-complementation to create replicon delivery particles (RDPs).

Spike-deleted replicon design and optimization of RNA production

The SARS-CoV-2 genome is thought to encode 16 non-structural proteins (Nsp1-Nsp16) in two overlapping reading frames (Orf1a/1b), as well as four structural proteins—spike (S),

membrane (M), envelope (E), nucleocapsid (N)—and at least seven accessory proteins (3a, 3c, 6, 7a, 7b, 8, and 9b) expressed from subgenomic RNAs or alternative reading frames (Fig. 1A) (*25*, *26*). We adopted a modular design to assemble a replicon consisting of all viral proteins with the exception of the primary structural glycoprotein spike (ΔS). The spike transcription-regulating sequence (TRS) was instead used to drive expression of a gene cassette consisting of neomycin-resistance (NeoR) and a reporter gene (nuclear-localized monomeric NeonGreen or secreted *Gaussia* luciferase) separated by a T2A ribosome shift sequence (Fig. 1A). The plasmid encoding the replicon cDNA contains an upstream T7 promoter at the 5′ end for in vitro transcription and a self-cleaving hepatitis delta virus (HDV) ribozyme at the 3′ end, which cleaves after an encoded polyA sequence yielding an authentic terminus.

For replicon assembly, we employed a recently published RNA virus reverse genetics system in the yeast *Saccharomyces cerevisiae* (*10*). This system leverages transformation associated recombination (TAR) to accurately assemble numerous, large overlapping DNA fragments (*27*). Transformation of yeast with equimolar ratios of replicon cDNA fragments led to efficient replicon assembly as assessed by multiplex PCR (fig. S1A). We performed restriction enzyme digests of the resulting DNA to assess plasmid integrity and observed that yeast-derived plasmids were contaminated with yeast genomic DNA and did not reveal the expected *Nde*I digest pattern (Fig. 1B). In an alternative approach, we propagated yeast assembled plasmids in bacteria, which boosted plasmid purity, as demonstrated previously (*10*). However, the overall DNA yield was suboptimal in both instances and, in the case of bacterial propagation, often resulted in mutations in the coding region of the viral RNA-dependent RNA polymerase (RdRp). To improve plasmid purity and yield, we developed a method that relies on selective enzymatic digestion of contaminating yeast DNA followed by preferential amplification of the plasmid product. We first treated plasmid preparations with *Bam*HI, which digests yeast genomic DNA but whose recognition sequence is absent in the replicon plasmid. We subsequently treated the DNA with Plasmid-Safe (PS) DNase to digest linear contaminating yeast DNA. We next turned to multiple displacement rolling circle amplification (RCA) using phi29 DNA polymerase (*28*) and random primers to amplify replicon plasmids from either yeast or sequence-verified bacterial clones. Due to its processivity (>70 kb per binding event), strand-displacement activity, and low error rate ($<1E^{-6}$), phi29 polymerase can exponentially amplify long, circular DNA sequences with high fidelity under isothermal conditions and has been used to replicate bacterial artificial chromosomes, cosmids, mitochondrial DNA, and microbial genomes megabases in length (*29*–*31*). Indeed, amplification of replicon plasmids from yeast or bacteria yielded useful quantities of full length and intact replicon DNA (Fig. 1B) as verified by amplicon sequencing. We used the resulting DNA for T7 transcription reactions and observed apparently full-length (~27.5 kb) replicon RNA in addition to shorter RNA products (Fig. 1C). To directly compare launch efficiency, mNeonGreen-encoding replicon RNAs were electroporated into BHK-21 cells together with additional in vitro transcribed N mRNA, previously shown to boost launch efficiency (*10*, *15*), and the percentage of cells expressing the mNeonGreen reporter was measured at 24 h post electroporation. We observed fewer than 1% of replicon positive cells using RNA from non-phi29 amplified templates, where bacteria propagated plasmids yielded the highest (0.8%) mNeonGreen reporter signal (Fig. 1D). In contrast, PS DNase-treated and phi29-amplified templates yielded approximately 20% mNeonGreen positive cells. The mNeonGreen signal was dependent on productive replication and transcription, as no signal was observed using replicon RNA harboring inactivating mutations in Nsp12, the viral RdRp (*32*, *33*) (Fig. 1E, pol(-)). In summary, the most efficient means of reaching high replicon launch efficiency was achieved by making capped RNA from yeast plasmids that are PS digested and phi29-amplified prior to transcription (Fig. 1F).

Spike-deleted SARS-CoV-2 replicons are convenient and versatile assay platforms

To characterize the utility of spike-deleted replicons for antiviral compound evaluation and screening, host factor validation, and viral mutant phenotype assessment, we constructed a replicon encoding secreted *Gaussia* luciferase (Gluc) (Fig. 1A). RNA transcripts were electroporated into Huh-7.5 cells and cumulative reporter activity per 24 h period was monitored via luciferase quantification from culture supernatant. We detected robust luciferase activity by 24 h post-electroporation which steadily decreased over time (Fig. 2A). Luciferase activity was dependent on viral replication, as no signal was observed for the pol(-) replicon mutant. Replicon-driven luciferase expression was also sensitive to remdesivir, a well-characterized inhibitor of the SARS-CoV-2 RdRp (*34*). As a luciferase-independent measure of replicon activity, we measured N subgenomic mRNA (sgmRNA) levels by qRT-PCR and observed RNA accumulation kinetics that paralleled reporter gene expression (Fig. 2B). This signal was distinct from co-electroporated N mRNA, needed for efficient replicon or virus launch from RNA (*10*), which was detectable in all measured samples as expected (fig. S1B). These results show that SARS-CoV-2 replicons undergo robust and quantifiable RNA replication.

One of the most fruitful applications for replicons are as drug screening platforms. As a proof-of-concept, we treated cells bearing luciferase reporter replicons with compounds reported to have direct acting antiviral (DAA) activity at

different stages of the viral life cycle: the SARS-CoV-2 RdRp inhibitor remdesivir (*34*), and masitinib, a proposed 3CLpro inhibitor (*35*). As a negative control, we used 27-hydroxycholesterol (27-HC), which was reported to have both anti-SARS-CoV-2 and anti-HCoV-OC43 activity by affecting viral entry, which we bypass by RNA transfection (*36*). We also tested a host-targeting agent (HTA), AM580, a retinoid derivative reported to have broad antiviral activity by disrupting sterol regulatory element binding protein (SREBP) lipid signaling (*37*). For remdesivir, we observed IC_{50} values and low cytotoxicity profiles similar to those previously reported for live virus (Fig. 2C) (*34*). For masitinib, we observed inhibition with similar IC_{50} values to what was shown with infectious HCoV-OC43 and SARS-CoV-2 (*35*). However, cellular toxicity was observed at high concentrations (Fig. 2D). For AM580, IC_{50} values were consistent with reported results, with low cytotoxicity at inhibitory concentrations (Fig. 2E) (*37*). As expected, 27-HC showed no detectable inhibition (Fig. 2F). These representative results showcase the utility of SARS-CoV-2 replicons as scalable drug discovery platforms focused on intracellular viral replication events.

We next tested if replicons could be used to identify or validate intracellular SARS-CoV-2 host factors. Transmembrane protein 41B (TMEM41B) was recently reported to be a critical intracellular host factor for multiple coronaviruses (*38*). To test whether SARS-CoV-2 replicon activity depended on TMEM41B function, we electroporated wild-type and TMEM41B KO Huh-7.5 cells with reporter replicon RNA. Consistent with results obtained with virus, TMEM41B KO resulted in a 22-fold decrease in reporter activity compared to wild-type Huh-7.5 cells (Fig. 2G), an effect similar in magnitude to remdesivir treatment. In contrast, an alphavirus replicon was not affected by TMEM41B ablation (Fig. 2H). TMEM41B reconstitution in KO cells led to an 11-fold rescue in replicon activity (Fig. 2I). These results demonstrate that SARS-CoV-2 replicons are sensitive to disruption of critical intracellular host factors.

SARS-CoV-2 replicons reveal viral determinants of interferon sensitivity

SARS-CoV-2 replicons can also be used to characterize viral mutants. Recent studies have highlighted the importance of Nsp1 as a suppressor of host translation (*39–44*) consistent with prior studies on SARS-CoV (*45*, *46*). This Nsp1 activity can be ablated by two amino acid substitutions at positions 164 and 165 (*47*) that are important for association with the 40S ribosomal subunit (*39*, *40*). Nsp1 mutant ($Nsp1^{mut}$) replicons might preserve translation and cell viability and hence prolong replicon activity or, given the proposed role of Nsp1 in blunting the innate immune response (*14*, *39*, *48*), such mutations might attenuate the replicon. We generated $Nsp1^{mut}$ replicons and found that they performed similarly to wild-type versions in Huh-7.5 cells, could be inhibited by remdesivir (Fig. 3A), but exhibited less cellular toxicity (Fig. 3B). In contrast, viability of cells harboring wild-type replicons remained low even when replicons were launched in the presence of remdesivir (Fig. 3B). Taken together, these results suggest that the initial burst of SARS-CoV-2 Nsp1, expressed from transfected replicon RNA, is an important mediator of cell toxicity.

As SARS-CoV-2 Nsp1 activity is proposed to halt production of interferon-stimulated gene (ISG) products (*39*, *40*), we hypothesized that $Nsp1^{mut}$ replicons would be more sensitive to interferon. Indeed, upon launch in Huh-7.5 cells, Nsp1 mutants were hypersensitive to IFNα and IFNβ compared to wild-type replicons (Fig. 3, C and D) while both replicons exhibited similar sensitivity to remdesivir (Fig. 3E). These results highlight the ability of Nsp1 to blunt the antiviral ISG response by inhibiting host translation, which contributes to cytotoxicity. While these features of $Nsp1^{mut}$ replicons may be advantageous for DAA and HTA screening, the lack of Nsp1 functions may affect screening outcomes in different cell backgrounds, depending upon innate immune competence or other factors. Further, attempts to leverage the lower cytotoxicity of $Nsp1^{mut}$ replicons to select for stable cell lines harboring non-cytopathic replicons, as done previously with HCoV-229E (*49*), have thus far been unsuccessful in Huh-7.5 and BHK-21 cells. Different cell backgrounds and multiple adaptive mutations may be necessary to achieve this goal.

SARS-CoV-2 replicons can be trans-complemented with spike to generate single-cycle virions

While BHK-21 and Huh-7.5 cells are permissive for replicon launch via electroporation, physiologically relevant lung cell lines and primary cell types are more challenging to transfect. Such cells also have intact RNA sensing and innate immune functions, which BHK-21 and Huh-7.5 cells lack (*50*, *51*). Electroporation or other methods of RNA delivery also preclude the study of normal viral entry processes, and the large quantities of transfected RNA do not mimic virus infection, where one or few RNA genomes initiate productive replication. As a more authentic route for replicon delivery, we attempted to package replicons as single-cycle virions that could infect cells in a spike-dependent manner but would not produce infectious progeny capable of further spread. We refer to these single-cycle virions as replicon delivery particles (RDPs). We transfected BHK-21 cells with a spike-expressing plasmid and 24 h later co-electroporated spike-deleted mNeonGreen replicon RNA and N mRNA (Fig. 4, A and B). The full-length native spike gene was human-codon optimized and designed to lack 5′UTR, leader, TRS, and 3′UTR viral sequences in order to minimize the possibility of recombination into the replicon (*52*, *53*). Given that Nsp1 hindered cell survival post electroporation, we hypothesized that $Nsp1^{mut}$ replicons could be

trans-complemented more efficiently than wild-type replicons. To test this, wild-type or Nsp1^{mut} replicons were electroporated into spike-expressing cells. After 24 h, the putative RDPs were concentrated from the supernatant of producer cells by polyethylene glycol (PEG) precipitation and introduced to the culture medium of recipient Huh-7.5 cells overexpressing ACE2 and TMPRSS2 (Huh-7.5 AT). We detected mNeonGreen-positive cells in a spike-dependent manner (Fig. 4, C and D, P1) with Nsp1^{mut} RDPs yielding a 5-fold higher proportion of positive cells. No mNeonGreen positive cells were detected upon further passage of the supernatant from RDP positive Huh-7.5 AT cells (P1) onto naïve Huh-7.5 AT cells (P2), (Fig. 4, C and D). This suggests that spike RDPs exhibit single-cycle infectivity. Consistent with this observation, spike expression was readily detected in the producer cells (P0), but absent in RDP-infected P1 cells (fig. S2, A and B). N protein expression was observed in producer and P1 cells, but not in P2 cells as expected (fig. S2C). Further, consistent with a single-cycle infectivity, spike RDP infection did not yield characteristic syncytia indicative of spike expression, as is typically seen with live virus (fig. S2, D and E). We observed spike RDP titers of 10^3-10^4 $TCID_{50}$/ml in Huh-7.5 AT using unconcentrated stocks and up to 6.2×10^5 $TCID_{50}$/ml upon concentration, with about 10-fold lower specific infectivity than virus (Fig. 4, E to G). This may indicate that RDPs have reduced spike density compared to virus.

Neutralization assays with RDPs recapitulate authentic SARS-CoV-2 antibody phenotypes

Spike RDPs could complement pseudovirus assays based on HIV-1 or vesicular stomatitis virus (VSV), with a SARS-CoV-2-based, and therefore more authentic, single cycle alternative. We generated Nsp1^{mut} Gluc RDPs trans-packaged with either the prototype spike (WA1/2020 isolate) or the B.1.351 variant of concern (*54*). We performed neutralization assays with these RDPs using two well-characterized human monoclonal antibodies, C135 and C144 capable of potently neutralizing SARS-CoV-2 or pseudoviruses expressing the prototype spike (*55*). C144 binding and neutralization is ablated by the spike E484K mutation, which was originally identified by antibody selection experiments using a VSV pseudovirus (*56*) and which later appeared in the B.1.351 variant (*54*). RDPs harboring the prototype spike were neutralized with both antibodies, while only C135, but not C144, neutralized B.1.351 spike RDPs (Fig. 5, A and B). Neutralization curves and relative IC_{50} values were comparable to those obtained with the respective SARS-CoV-2 isolates (Fig. 5, D to F) and with previous reports using pseudovirus assays (*55*, *56*) with the added advantage that RDP generation does not require deletions in the spike coding sequence (*22*).

Spike-deleted SARS-CoV-2 replicons can incorporate VSV glycoprotein

Efficient entry with spike RDPs appears to require high levels of ACE2 and TMPRSS2 in Huh-7.5 cells since RDP addition to Huh-7.5 or Vero cells not overexpressing these factors was unproductive. As numerous cell lines relevant for SARS-CoV-2 studies may have insufficient ACE2 or TMPRSS2 levels unless engineered to support viral entry (*57*), we tested whether spike-deleted replicons could be packaged with VSV-G, analogous to one-way lentiviral transduction. In such systems, VSV-G pseudotyping provides an efficient means of lentiviral vector entry (*58*) and for RDPs might provide an ACE2-independent means of replicon delivery. We transfected BHK-21 cells with a VSV-G expression plasmid followed by co-electroporation with spike-deleted mNeonGreen replicon RNA and N mRNA (Fig. 6A). The resulting RDPs in the supernatant were concentrated and added to naïve Huh-7.5 cells. After 24 h, we observed mNeonGreen-positive cells in a VSV-G dependent manner (Fig. 6, B and C, P1). Importantly, there was no measurable signal in a second passage (Fig. 6, B and C, P2) and no VSV-G carryover was detected in the P1 cells (fig. S3, A and B), suggesting that VSV-G RDPs possess single-cycle infectivity. In contrast to spike RDPs, producer (P0) VSV-G expressing BHK-21 cells had a significantly higher ratio of mNeonGreen compared to control cells when the Nsp1^{mut} replicon was used, suggestive of local VSV-G dependent spread. P1 cells also exhibited higher mNeonGreen and N protein positivity using Nsp1^{mut} RDPs compared to wild-type RDPs (Fig. 6, B and C, and fig. S3C). We observed VSV-G RDP titers between 1-8x10^3 $TCID_{50}$/ml on Huh-7.5 cells. We then tested VSV-G RDP infectivity on additional cell types: African green monkey VeroE6 cells, human Caco2 intestinal epithelial cells (*59*, *60*) and both Calu3 and A549 human lung adenocarcinoma cells (*61*–*64*). Additionally, we tested RDP activity in two lines of human airway cells: normal human bronchial/tracheal epithelial cells (NHBE) and normal human lung fibroblasts (NHLF) (Fig. 6D). All cells were susceptible to infection with VSV-G RDPs and exhibited productive viral replication, as evident from mNeonGreen reporter expression. We pretreated NHBE, NHLF and A549 cells with 100 nM remdesivir or 100 pM IFNα and subsequently added Gluc VSV-G RDPs. As shown in Fig. 6E, both treatments inhibited Gluc reporter activity to levels approaching baseline. Overall, these data demonstrate that VSV-G RDPs provide flexible means of replicon launch in primary cell contexts.

To further examine whether VSV-G RDPs have single-cycle infectivity using a more sensitive approach, we generated Gluc expressing VSV-G RDPs and infected naïve Huh-7.5 cells followed by Gluc measurements 24h later (P1). While the VSV-G RDP positive cells produced a robust Gluc signal, no signal was observed when the supernatant from P1 was concentrated and passaged onto naïve cells (P2) (Fig. 6F). Input

supernatant was similarly concentrated and used as positive control for VSV-G RDP concentration and singe-cycle infection.

VSV-G trans-complementation of a SARS-CoV-2 replicon lacking all accessory genes

As the RDP results presented thus far have relied on spike-deleted replicons with or without mutations in Nsp1, we examined whether replicons harboring additional deletions could be packaged into RDPs. Previous work with SARS-CoV and SARS-CoV-2 has shown that M and E genes are required for virion morphogenesis and release (*65*, *66*). On that basis, we tested VSV-G packaging of a replicon that lacked all accessory genes but retained genes encoding the structural M, E, and N proteins (ΔAcc) (fig. S4A). VSV-G dependent infectivity was observed on recipient Huh-7.5 cells using both mNeonGreen and Gluc ΔAcc versions at levels comparable to the spike-deleted (ΔS) replicon (fig. S4, B to D). Thus, accessory genes are dispensable for replicon pseudotyping with VSV-G.

We highlight several features of SARS-CoV-2 RDPs. First, RDPs enable a rapid and isogenic means of generating single-cycle SARS-CoV-2 virions for spike-directed efforts, such as screening spike variants against neutralizing antibodies while eliminating the potential for spike mutations to arise, as is commonly encountered with cell culture passaged virus (*67*, *68*) and for screening antibodies against additional structural proteins. Second, single-cycle infectivity can be achieved with VSV-G, which may be beneficial for studies in systems where ACE2 receptor overexpression is infeasible. Finally, in contrast to replicon launch by RNA transfection, RDPs can be frozen, stored, and distributed, since they require no specialized equipment to use once generated. These attributes could eventually enable high-throughput drug screening without the need for high containment. Overall, these features highlight how SARS-CoV-2 spike-deleted replicons and RDPs provide a flexible and single-cycle infectious platform for future studies of this pandemic virus.

REFERENCES AND NOTES

1. R. French, P. Ahlquist, Intercistronic as well as terminal sequences are required for efficient amplification of brome mosaic virus RNA3. *J. Virol.* **61**, 1457–1465 (1987). doi:10.1128/jvi.61.5.1457-1465.1987 Medline
2. P. J. Bredenbeek, I. Frolov, C. M. Rice, S. Schlesinger, Sindbis virus expression vectors: Packaging of RNA replicons by using defective helper RNAs. *J. Virol.* **67**, 6439–6446 (1993). doi:10.1128/jvi.67.11.6439-6446.1993 Medline
3. V. Lohmann, F. Körner, J. Koch, U. Herian, L. Theilmann, R. Bartenschlager, Replication of subgenomic hepatitis C virus RNAs in a hepatoma cell line. *Science* **285**, 110–113 (1999). doi:10.1126/science.285.5424.110 Medline
4. K. J. Blight, A. A. Kolykhalov, C. M. Rice, Efficient initiation of HCV RNA replication in cell culture. *Science* **290**, 1972–1974 (2000). doi:10.1126/science.290.5498.1972 Medline
5. V. Thiel, J. Herold, B. Schelle, S. G. Siddell, Viral replicase gene products suffice for coronavirus discontinuous transcription. *J. Virol.* **75**, 6676–6681 (2001). doi:10.1128/JVI.75.14.6676-6681.2001 Medline
6. K. M. Curtis, B. Yount, R. S. Baric, Heterologous gene expression from transmissible gastroenteritis virus replicon particles. *J. Virol.* **76**, 1422–1434 (2002). doi:10.1128/JVI.76.3.1422-1434.2002 Medline
7. G. Kaplan, V. R. Racaniello, Construction and characterization of poliovirus subgenomic replicons. *J. Virol.* **62**, 1687–1696 (1988). doi:10.1128/jvi.62.5.1687-1696.1988 Medline
8. H. Hannemann, Viral replicons as valuable tools for drug discovery. *Drug Discov. Today* **25**, 1026–1033 (2020). doi:10.1016/j.drudis.2020.03.010 Medline
9. V. Lohmann, Hepatitis C virus cell culture models: An encomium on basic research paving the road to therapy development. *Med. Microbiol. Immunol.* **208**, 3–24 (2019). doi:10.1007/s00430-018-0566-x Medline
10. T. Thi Nhu Thao, F. Labroussaa, N. Ebert, P. V'kovski, H. Stalder, J. Portmann, J. Kelly, S. Steiner, M. Holwerda, A. Kratzel, M. Gultom, K. Schmied, L. Laloli, L. Hüsser, M. Wider, S. Pfaender, D. Hirt, V. Cippà, S. Crespo-Pomar, S. Schröder, D. Muth, D. Niemeyer, V. M. Corman, M. A. Müller, C. Drosten, R. Dijkman, J. Jores, V. Thiel, Rapid reconstruction of SARS-CoV-2 using a synthetic genomics platform. *Nature* **582**, 561–565 (2020). doi:10.1038/s41586-020-2294-9 Medline
11. Y. J. Hou, K. Okuda, C. E. Edwards, D. R. Martinez, T. Asakura, K. H. Dinnon 3rd, T. Kato, R. E. Lee, B. L. Yount, T. M. Mascenik, G. Chen, K. N. Olivier, A. Ghio, L. V. Tse, S. R. Leist, L. E. Gralinski, A. Schäfer, H. Dang, R. Gilmore, S. Nakano, L. Sun, M. L. Fulcher, A. Livraghi-Butrico, N. I. Nicely, M. Cameron, C. Cameron, D. J. Kelvin, A. de Silva, D. M. Margolis, A. Markmann, L. Bartelt, R. Zumwalt, F. J. Martinez, S. P. Salvatore, A. Borczuk, P. R. Tata, V. Sontake, A. Kimple, I. Jaspers, W. K. O'Neal, S. H. Randell, R. C. Boucher, R. S. Baric, SARS-CoV-2 Reverse Genetics Reveals a Variable Infection Gradient in the Respiratory Tract. *Cell* **182**, 429–446.e14 (2020). doi:10.1016/j.cell.2020.05.042 Medline
12. S. Torii, C. Ono, R. Suzuki, Y. Morioka, I. Anzai, Y. Fauzyah, Y. Maeda, W. Kamitani, T. Fukuhara, Y. Matsuura, Establishment of a reverse genetics system for SARS-CoV-2 using circular polymerase extension reaction. *Cell Rep.* **35**, 109014 (2021). doi:10.1016/j.celrep.2021.109014 Medline
13. S. J. Rihn, A. Merits, S. Bakshi, M. L. Turnbull, A. Wickenhagen, A. J. T. Alexander, C. Baillie, B. Brennan, F. Brown, K. Brunker, S. R. Bryden, K. A. Burness, S. Carmichael, S. J. Cole, V. M. Cowton, P. Davies, C. Davis, G. De Lorenzo, C. L. Donald, M. Dorward, J. I. Dunlop, M. Elliott, M. Fares, A. da Silva Filipe, J. R. Freitas, W. Furnon, R. J. Gestuveo, A. Geyer, D. Giesel, D. M. Goldfarb, N. Goodman, R. Gunson, C. J. Hastie, V. Herder, J. Hughes, C. Johnson, N. Johnson, A. Kohl, K. Kerr, H. Leech, L. S. Lello, K. Li, G. Lieber, X. Liu, R. Lingala, C. Loney, D. Mair, M. J. McElwee, S. McFarlane, J. Nichols, K. Nomikou, A. Orr, R. J. Orton, M. Palmarini, Y. A. Parr, R. M. Pinto, S. Raggett, E. Reid, D. L. Robertson, J. Royle, N. Cameron-Ruiz, J. G. Shepherd, K. Smollett, D. G. Stewart, M. Stewart, E. Sugrue, A. M. Szemiel, A. Taggart, E. C. Thomson, L. Tong, L. S. Torrie, R. Toth, M. Varjak, S. Wang, S. G. Wilkinson, P. G. Wyatt, E. Zusinaite, D. R. Alessi, A. H. Patel, A. Zaid, S. J. Wilson, S. Mahalingam, A plasmid DNA-launched SARS-CoV-2 reverse genetics system and coronavirus toolkit for COVID-19 research. *PLOS Biol.* **19**, e3001091 (2021). doi:10.1371/journal.pbio.3001091 Medline
14. H. Xia, Z. Cao, X. Xie, X. Zhang, J. Y.-C. Chen, H. Wang, V. D. Menachery, R. Rajsbaum, P.-Y. Shi, Evasion of Type I Interferon by SARS-CoV-2. *Cell Rep.* **33**, 108234 (2020). doi:10.1016/j.celrep.2020.108234 Medline
15. Y. Zhang, W. Song, S. Chen, Z. Yuan, Z. Yi, A bacterial artificial chromosome (BAC)-vectored noninfectious replicon of SARS-CoV-2. *Antiviral Res.* **185**, 104974 (2021). doi:10.1016/j.antiviral.2020.104974 Medline
16. X. He, S. Quan, M. Xu, S. Rodriguez, S. L. Goh, J. Wei, A. Fridman, K. A. Koeplinger, S. S. Carroll, J. A. Grobler, A. S. Espeseth, D. B. Olsen, D. J. Hazuda, D. Wang, Generation of SARS-CoV-2 reporter replicon for high-throughput antiviral screening and testing. *Proc. Natl. Acad. Sci. U.S.A.* **118**, e2025866118 (2021). doi:10.1073/pnas.2025866118 Medline
17. Y. Luo, F. Yu, M. Zhou, Y. Liu, B. Xia, X. Zhang, J. Liu, J. Zhang, Y. Du, R. Li, L. Wu, X. Zhang, T. Pan, D. Guo, T. Peng, H. Zhang, Engineering a Reliable and Convenient SARS-CoV-2 Replicon System for Analysis of Viral RNA Synthesis and Screening of Antiviral Inhibitors. *mBio* **12**, e02754-20 (2021). doi:10.1128/mBio.02754-20 Medline
18. X. Ju, Y. Zhu, Y. Wang, J. Li, J. Zhang, M. Gong, W. Ren, S. Li, J. Zhong, L. Zhang, Q. C. Zhang, R. Zhang, Q. Ding, A novel cell culture system modeling the SARS-CoV-2 life cycle. *PLOS Pathog.* **17**, e1009439 (2021). doi:10.1371/journal.ppat.1009439 Medline

19. X. Zhang, Y. Liu, J. Liu, A. L. Bailey, K. S. Plante, J. A. Plante, J. Zou, H. Xia, N. E. Bopp, P. V. Aguilar, P. Ren, V. D. Menachery, M. S. Diamond, S. C. Weaver, X. Xie, P.-Y. Shi, A trans-complementation system for SARS-CoV-2 recapitulates authentic viral replication without virulence. *Cell* **184**, 2229–2238.e13 (2021). doi:10.1016/j.cell.2021.02.044 Medline
20. X. Ou, Y. Liu, X. Lei, P. Li, D. Mi, L. Ren, L. Guo, R. Guo, T. Chen, J. Hu, Z. Xiang, Z. Mu, X. Chen, J. Chen, K. Hu, Q. Jin, J. Wang, Z. Qian, Characterization of spike glycoprotein of SARS-CoV-2 on virus entry and its immune cross-reactivity with SARS-CoV. *Nat. Commun.* **11**, 1620–12 (2020). doi:10.1038/s41467-020-15562-9 Medline
21. K. H. D. Crawford, R. Eguia, A. S. Dingens, A. N. Loes, K. D. Malone, C. R. Wolf, H. Y. Chu, M. A. Tortorici, D. Veesler, M. Murphy, D. Pettie, N. P. King, A. B. Balazs, J. D. Bloom, Protocol and Reagents for Pseudotyping Lentiviral Particles with SARS-CoV-2 Spike Protein for Neutralization Assays. *Viruses* **12**, 513 (2020). doi:10.3390/v12050513 Medline
22. F. Schmidt, Y. Weisblum, F. Muecksch, H.-H. Hoffmann, E. Michailidis, J. C. C. Lorenzi, P. Mendoza, M. Rutkowska, E. Bednarski, C. Gaebler, M. Agudelo, A. Cho, Z. Wang, A. Gazumyan, M. Cipolla, M. Caskey, D. F. Robbiani, M. C. Nussenzweig, C. M. Rice, T. Hatziioannou, P. D. Bieniasz, Measuring SARS-CoV-2 neutralizing antibody activity using pseudotyped and chimeric viruses. *J. Exp. Med.* **217**, e20201181 (2020). doi:10.1084/jem.20201181 Medline
23. J. Nie, Q. Li, J. Wu, C. Zhao, H. Hao, H. Liu, L. Zhang, L. Nie, H. Qin, M. Wang, Q. Lu, X. Li, Q. Sun, J. Liu, C. Fan, W. Huang, M. Xu, Y. Wang, Establishment and validation of a pseudovirus neutralization assay for SARS-CoV-2. *Emerg. Microbes Infect.* **9**, 680–686 (2020). doi:10.1080/22221751.2020.1743767 Medline
24. M. Chen, X.-E. Zhang, Construction and applications of SARS-CoV-2 pseudoviruses: A mini review. *Int. J. Biol. Sci.* **17**, 1574–1580 (2021). doi:10.7150/ijbs.59184 Medline
25. A. Wu, Y. Peng, B. Huang, X. Ding, X. Wang, P. Niu, J. Meng, Z. Zhu, Z. Zhang, J. Wang, J. Sheng, L. Quan, Z. Xia, W. Tan, G. Cheng, T. Jiang, Genome Composition and Divergence of the Novel Coronavirus (2019-nCoV) Originating in China. *Cell Host Microbe* **27**, 325–328 (2020). doi:10.1016/j.chom.2020.02.001 Medline
26. I. Jungreis, R. Sealfon, M. Kellis, SARS-CoV-2 gene content and COVID-19 mutation impact by comparing 44 Sarbecovirus genomes. *Nat. Commun.* **12**, 2642–20 (2021). doi:10.1038/s41467-021-22905-7 Medline
27. N. Kouprina, V. Larionov, Transformation-associated recombination (TAR) cloning for genomics studies and synthetic biology. *Chromosoma* **125**, 621–632 (2016). doi:10.1007/s00412-016-0588-3 Medline
28. L. Blanco, A. Bernad, J. M. Lázaro, G. Martín, C. Garmendia, M. Salas, Highly efficient DNA synthesis by the phage phi 29 DNA polymerase. Symmetrical mode of DNA replication. *J. Biol. Chem.* **264**, 8935–8940 (1989). doi:10.1016/S0021-9258(18)81883-X Medline
29. F. B. Dean, J. R. Nelson, T. L. Giesler, R. S. Lasken, Rapid amplification of plasmid and phage DNA using Phi 29 DNA polymerase and multiply-primed rolling circle amplification. *Genome Res.* **11**, 1095–1099 (2001). doi:10.1101/gr.180501 Medline
30. J. A. Esteban, M. Salas, L. Blanco, Fidelity of phi 29 DNA polymerase. Comparison between protein-primed initiation and DNA polymerization. *J. Biol. Chem.* **268**, 2719–2726 (1993). doi:10.1016/S0021-9258(18)53833-3 Medline
31. J. Banér, M. Nilsson, M. Mendel-Hartvig, U. Landegren, Signal amplification of padlock probes by rolling circle replication. *Nucleic Acids Res.* **26**, 5073–5078 (1998). doi:10.1093/nar/26.22.5073 Medline
32. Y. Gao, L. Yan, Y. Huang, F. Liu, Y. Zhao, L. Cao, T. Wang, Q. Sun, Z. Ming, L. Zhang, J. Ge, L. Zheng, Y. Zhang, H. Wang, Y. Zhu, C. Zhu, T. Hu, T. Hua, B. Zhang, X. Yang, J. Li, H. Yang, Z. Liu, W. Xu, L. W. Guddat, Q. Wang, Z. Lou, Z. Rao, Structure of the RNA-dependent RNA polymerase from COVID-19 virus. *Science* **368**, 779–782 (2020). doi:10.1126/science.abb7498 Medline
33. C. J. Gordon, E. P. Tchesnokov, E. Woolner, J. K. Perry, J. Y. Feng, D. P. Porter, M. Götte, Remdesivir is a direct-acting antiviral that inhibits RNA-dependent RNA polymerase from severe acute respiratory syndrome coronavirus 2 with high potency. *J. Biol. Chem.* **295**, 6785–6797 (2020). doi:10.1074/jbc.RA120.013679 Medline
34. A. J. Pruijssers, A. S. George, A. Schäfer, S. R. Leist, L. E. Gralinksi, K. H. Dinnon 3rd, B. L. Yount, M. L. Agostini, L. J. Stevens, J. D. Chappell, X. Lu, T. M. Hughes, K. Gully, D. R. Martinez, A. J. Brown, R. L. Graham, J. K. Perry, V. Du Pont, J. Pitts, B. Ma, D. Babusis, E. Murakami, J. Y. Feng, J. P. Bilello, D. P. Porter, T. Cihlar, R. S. Baric, M. R. Denison, T. P. Sheahan, Remdesivir Inhibits SARS-CoV-2 in Human Lung Cells and Chimeric SARS-CoV Expressing the SARS-CoV-2 RNA Polymerase in Mice. *Cell Rep.* **32**, 107940 (2020). doi:10.1016/j.celrep.2020.107940 Medline
35. N. Drayman *et al.*, Drug repurposing screen identifies masitinib as a 3CLpro inhibitor that blocks replication of SARS-CoV-2 in vitro. bioRxiv 2020.08.31.274639 (2020); https://doi.org/10.1101/2020.08.31.274639.
36. A. Marcello, A. Civra, R. Milan Bonotto, L. Nascimento Alves, S. Rajasekharan, C. Giacobone, C. Caccia, R. Cavalli, M. Adami, P. Brambilla, D. Lembo, G. Poli, V. Leoni, The cholesterol metabolite 27-hydroxycholesterol inhibits SARS-CoV-2 and is markedly decreased in COVID-19 patients. *Redox Biol.* **36**, 101682 (2020). doi:10.1016/j.redox.2020.101682 Medline
37. S. Yuan, H. Chu, J. F.-W. Chan, Z.-W. Ye, L. Wen, B. Yan, P.-M. Lai, K.-M. Tee, J. Huang, D. Chen, C. Li, X. Zhao, D. Yang, M. C. Chiu, C. Yip, V. K.-M. Poon, C. C.-S. Chan, K.-H. Sze, J. Zhou, I. H.-Y. Chan, K.-H. Kok, K. K.-W. To, R. Y.-T. Kao, J. Y.-N. Lau, D.-Y. Jin, S. Perlman, K.-Y. Yuen, SREBP-dependent lipidomic reprogramming as a broad-spectrum antiviral target. *Nat. Commun.* **10**, 120–15 (2019). doi:10.1038/s41467-018-08015-x Medline
38. W. M. Schneider, J. M. Luna, H.-H. Hoffmann, F. J. Sánchez-Rivera, A. A. Leal, A. W. Ashbrook, J. Le Pen, I. Ricardo-Lax, E. Michailidis, A. Peace, A. F. Stenzel, S. W. Lowe, M. R. MacDonald, C. M. Rice, J. T. Poirier, Genome-Scale Identification of SARS-CoV-2 and Pan-coronavirus Host Factor Networks. *Cell* **184**, 120–132.e14 (2021). doi:10.1016/j.cell.2020.12.006 Medline
39. M. Thoms, R. Buschauer, M. Ameismeier, L. Koepke, T. Denk, M. Hirschenberger, H. Kratzat, M. Hayn, T. Mackens-Kiani, J. Cheng, J. H. Straub, C. M. Stürzel, T. Fröhlich, O. Berninghausen, T. Becker, F. Kirchhoff, K. M. J. Sparrer, R. Beckmann, Structural basis for translational shutdown and immune evasion by the Nsp1 protein of SARS-CoV-2. *Science* **369**, 1249–1255 (2020). doi:10.1126/science.abc8665 Medline
40. K. Schubert, E. D. Karousis, A. Jomaa, A. Scaiola, B. Echeverria, L.-A. Gurzeler, M. Leibundgut, V. Thiel, O. Mühlemann, N. Ban, SARS-CoV-2 Nsp1 binds the ribosomal mRNA channel to inhibit translation. *Nat. Struct. Mol. Biol.* **27**, 959–966 (2020). doi:10.1038/s41594-020-0511-8 Medline
41. C. P. Lapointe, R. Grosely, A. G. Johnson, J. Wang, I. S. Fernández, J. D. Puglisi, Dynamic competition between SARS-CoV-2 NSP1 and mRNA on the human ribosome inhibits translation initiation. *Proc. Natl. Acad. Sci. U.S.A.* **118**, e2017715118 (2021). doi:10.1073/pnas.2017715118 Medline
42. A. Tidu, A. Janvier, L. Schaeffer, P. Sosnowski, L. Kuhn, P. Hammann, E. Westhof, G. Eriani, F. Martin, The viral protein NSP1 acts as a ribosome gatekeeper for shutting down host translation and fostering SARS-CoV-2 translation. *RNA* **27**, 253–264 (2020). doi:10.1261/rna.078121.120 Medline
43. A. K. Banerjee, M. R. Blanco, E. A. Bruce, D. D. Honson, L. M. Chen, A. Chow, P. Bhat, N. Ollikainen, S. A. Quinodoz, C. Loney, J. Thai, Z. D. Miller, A. E. Lin, M. M. Schmidt, D. G. Stewart, D. Goldfarb, G. De Lorenzo, S. J. Rihn, R. M. Voorhees, J. W. Botten, D. Majumdar, M. Guttman, SARS-CoV-2 Disrupts Splicing, Translation, and Protein Trafficking to Suppress Host Defenses. *Cell* **183**, 1325–1339.e21 (2020). doi:10.1016/j.cell.2020.10.004 Medline
44. Y. Finkel, A. Gluck, A. Nachshon, R. Winkler, T. Fisher, B. Rozman, O. Mizrahi, Y. Lubelsky, B. Zuckerman, B. Slobodin, Y. Yahalom-Ronen, H. Tamir, I. Ulitsky, T. Israely, N. Paran, M. Schwartz, N. Stern-Ginossar, SARS-CoV-2 uses a multipronged strategy to impede host protein synthesis. *Nature* **594**, 240–245 (2021). doi:10.1038/s41586-021-03610-3 Medline
45. K. G. Lokugamage, K. Narayanan, C. Huang, S. Makino, Severe acute respiratory syndrome coronavirus protein nsp1 is a novel eukaryotic translation inhibitor that represses multiple steps of translation initiation. *J. Virol.* **86**, 13598–13608 (2012). doi:10.1128/JVI.01958-12 Medline
46. W. Kamitani, C. Huang, K. Narayanan, K. G. Lokugamage, S. Makino, A two-pronged strategy to suppress host protein synthesis by SARS coronavirus Nsp1 protein. *Nat. Struct. Mol. Biol.* **16**, 1134–1140 (2009). doi:10.1038/nsmb.1680 Medline
47. K. Narayanan, C. Huang, K. Lokugamage, W. Kamitani, T. Ikegami, C.-T. K. Tseng, S. Makino, Severe acute respiratory syndrome coronavirus nsp1 suppresses host gene expression, including that of type I interferon, in infected cells. *J. Virol.* **82**, 4471–4479 (2008). doi:10.1128/JVI.02472-07 Medline
48. X. Lei, X. Dong, R. Ma, W. Wang, X. Xiao, Z. Tian, C. Wang, Y. Wang, L. Li, L. Ren, F. Guo, Z. Zhao, Z. Zhou, Z. Xiang, J. Wang, Activation and evasion of type I interferon

responses by SARS-CoV-2. *Nat. Commun.* **11**, 3810–3812 (2020). doi:10.1038/s41467-020-17665-9 Medline
49. T. Hertzig, E. Scandella, B. Schelle, J. Ziebuhr, S. G. Siddell, B. Ludewig, V. Thiel, Rapid identification of coronavirus replicase inhibitors using a selectable replicon RNA. *J. Gen. Virol.* **85**, 1717–1725 (2004). doi:10.1099/vir.0.80044-0 Medline
50. R. Sumpter Jr., Y.-M. Loo, E. Foy, K. Li, M. Yoneyama, T. Fujita, S. M. Lemon, M. Gale Jr., Regulating intracellular antiviral defense and permissiveness to hepatitis C virus RNA replication through a cellular RNA helicase, RIG-I. *J. Virol.* **79**, 2689–2699 (2005). doi:10.1128/JVI.79.5.2689-2699.2005 Medline
51. M. Habjan, N. Penski, M. Spiegel, F. Weber, T7 RNA polymerase-dependent and -independent systems for cDNA-based rescue of Rift Valley fever virus. *J. Gen. Virol.* **89**, 2157–2166 (2008). doi:10.1099/vir.0.2008/002097-0 Medline
52. D. Dufour, P. A. Mateos-Gomez, L. Enjuanes, J. Gallego, I. Sola, Structure and functional relevance of a transcription-regulating sequence involved in coronavirus discontinuous RNA synthesis. *J. Virol.* **85**, 4963–4973 (2011). doi:10.1128/JVI.02317-10 Medline
53. I. Sola, F. Almazán, S. Zúñiga, L. Enjuanes, Continuous and Discontinuous RNA Synthesis in Coronaviruses. *Annu. Rev. Virol.* **2**, 265–288 (2015). doi:10.1146/annurev-virology-100114-055218 Medline
54. H. Tegally, E. Wilkinson, M. Giovanetti, A. Iranzadeh, V. Fonseca, J. Giandhari, D. Doolabh, S. Pillay, E. J. San, N. Msomi, K. Mlisana, A. von Gottberg, S. Walaza, M. Allam, A. Ismail, T. Mohale, A. J. Glass, S. Engelbrecht, G. Van Zyl, W. Preiser, F. Petruccione, A. Sigal, D. Hardie, G. Marais, N. Y. Hsiao, S. Korsman, M.-A. Davies, L. Tyers, I. Mudau, D. York, C. Maslo, D. Goedhals, S. Abrahams, O. Laguda-Akingba, A. Alisoltani-Dehkordi, A. Godzik, C. K. Wibmer, B. T. Sewell, J. Lourenço, L. C. J. Alcantara, S. L. Kosakovsky Pond, S. Weaver, D. Martin, R. J. Lessells, J. N. Bhiman, C. Williamson, T. de Oliveira, Detection of a SARS-CoV-2 variant of concern in South Africa. *Nature* **592**, 438–443 (2021). doi:10.1038/s41586-021-03402-9 Medline
55. D. F. Robbiani, C. Gaebler, F. Muecksch, J. C. C. Lorenzi, Z. Wang, A. Cho, M. Agudelo, C. O. Barnes, A. Gazumyan, S. Finkin, T. Hägglöf, T. Y. Oliveira, C. Viant, A. Hurley, H.-H. Hoffmann, K. G. Millard, R. G. Kost, M. Cipolla, K. Gordon, F. Bianchini, S. T. Chen, V. Ramos, R. Patel, J. Dizon, I. Shimeliovich, P. Mendoza, H. Hartweger, L. Nogueira, M. Pack, J. Horowitz, F. Schmidt, Y. Weisblum, E. Michailidis, A. W. Ashbrook, E. Waltari, J. E. Pak, K. E. Huey-Tubman, N. Koranda, P. R. Hoffman, A. P. West Jr., C. M. Rice, T. Hatziioannou, P. J. Bjorkman, P. D. Bieniasz, M. Caskey, M. C. Nussenzweig, Convergent antibody responses to SARS-CoV-2 in convalescent individuals. *Nature* **584**, 437–442 (2020). doi:10.1038/s41586-020-2456-9 Medline
56. Y. Weisblum, F. Schmidt, F. Zhang, J. DaSilva, D. Poston, J. C. Lorenzi, F. Muecksch, M. Rutkowska, H. H. Hoffmann, E. Michailidis, C. Gaebler, M. Agudelo, A. Cho, Z. Wang, A. Gazumyan, M. Cipolla, L. Luchsinger, C. D. Hillyer, M. Caskey, D. F. Robbiani, C. M. Rice, M. C. Nussenzweig, T. Hatziioannou, P. D. Bieniasz, Escape from neutralizing antibodies by SARS-CoV-2 spike protein variants. *eLife* **9**, e61312 (2020). doi:10.7554/eLife.61312 Medline
57. D.-Y. Chen *et al.*, SARS-CoV-2 desensitizes host cells to interferon through inhibition of the JAK-STAT pathway. bioRxiv 2020.10.27.358259 (2020); https://doi.org/10.1101/2020.10.27.358259.
58. J. Cronin, X.-Y. Zhang, J. Reiser, Altering the tropism of lentiviral vectors through pseudotyping. *Curr. Gene Ther.* **5**, 387–398 (2005). doi:10.2174/1566523054546224 Medline
59. K. Verhoeckx *et al.*, Caco-2 Cell Line. *The Impact of Food Bioactives on Health* (2015), 175, 103–111.
60. M. Letko, A. Marzi, V. Munster, Functional assessment of cell entry and receptor usage for SARS-CoV-2 and other lineage B betacoronaviruses. *Nat. Microbiol.* **5**, 562–569 (2020). doi:10.1038/s41564-020-0688-y Medline
61. Y. Zhu, A. Chidekel, T. H. Shaffer, Cultured human airway epithelial cells (calu-3): A model of human respiratory function, structure, and inflammatory responses. *Crit. Care Res. Pract.* **2010**, 1–8 (2010). doi:10.1155/2010/394578 Medline
62. V. Cagno, SARS-CoV-2 cellular tropism. *Lancet Microbe* **1**, e2–e3 (2020). doi:10.1016/S2666-5247(20)30008-2 Medline
63. D. J. Giard, S. A. Aaronson, G. J. Todaro, P. Arnstein, J. H. Kersey, H. Dosik, W. P. Parks, In vitro cultivation of human tumors: Establishment of cell lines derived from a series of solid tumors. *J. Natl. Cancer Inst.* **51**, 1417–1423 (1973). doi:10.1093/jnci/51.5.1417 Medline
64. D. Blanco-Melo, B. E. Nilsson-Payant, W.-C. Liu, S. Uhl, D. Hoagland, R. Møller, T. X. Jordan, K. Oishi, M. Panis, D. Sachs, T. T. Wang, R. E. Schwartz, J. K. Lim, R. A. Albrecht, B. R. tenOever, Imbalanced Host Response to SARS-CoV-2 Drives Development of COVID-19. *Cell* **181**, 1036–1045.e9 (2020). doi:10.1016/j.cell.2020.04.026 Medline
65. Y. L. Siu, K. T. Teoh, J. Lo, C. M. Chan, F. Kien, N. Escriou, S. W. Tsao, J. M. Nicholls, R. Altmeyer, J. S. M. Peiris, R. Bruzzone, B. Nal, The M, E, and N structural proteins of the severe acute respiratory syndrome coronavirus are required for efficient assembly, trafficking, and release of virus-like particles. *J. Virol.* **82**, 11318–11330 (2008). doi:10.1128/JVI.01052-08 Medline
66. M. Lu, P. D. Uchil, W. Li, D. Zheng, D. S. Terry, J. Gorman, W. Shi, B. Zhang, T. Zhou, S. Ding, R. Gasser, J. Prévost, G. Beaudoin-Bussières, S. P. Anand, A. Laumaea, J. R. Grover, L. Liu, D. D. Ho, J. R. Mascola, A. Finzi, P. D. Kwong, S. C. Blanchard, W. Mothes, Real-Time Conformational Dynamics of SARS-CoV-2 Spikes on Virus Particles. *Cell Host Microbe* **28**, 880–891.e8 (2020). doi:10.1016/j.chom.2020.11.001 Medline
67. S. Ramirez, C. Fernandez-Antunez, A. Galli, A. Underwood, L. V. Pham, L. A. Ryberg, S. Feng, M. S. Pedersen, L. S. Mikkelsen, S. Belouzard, J. Dubuisson, C. Sølund, N. Weis, J. M. Gottwein, U. Fahnøe, J. Bukh, Overcoming culture restriction for SARS-CoV-2 in human cells facilitates the screening of compounds inhibiting viral replication. *Antimicrob. Agents Chemother.* **65**, e0009721 (2021). doi:10.1128/AAC.00097-21 Medline
68. Z. Liu, H. Zheng, H. Lin, M. Li, R. Yuan, J. Peng, Q. Xiong, J. Sun, B. Li, J. Wu, L. Yi, X. Peng, H. Zhang, W. Zhang, R. J. G. Hulswit, N. Loman, A. Rambaut, C. Ke, T. A. Bowden, O. G. Pybus, J. Lu, Identification of Common Deletions in the Spike Protein of Severe Acute Respiratory Syndrome Coronavirus 2. *J. Virol.* **94**, e00790-20 (2020). doi:10.1128/JVI.00790-20 Medline
69. R. Sanchez-Velazquez, G. de Lorenzo, R. Tandavanitj, C. Setthapramote, P. J. Bredenbeek, L. Bozzacco, M. R. MacDonald, J. J. Clark, C. M. Rice, A. H. Patel, A. Kohl, M. Varjak, Generation of a reporter yellow fever virus for high throughput antiviral assays. *Antiviral Res.* **183**, 104939 (2020). doi:10.1016/j.antiviral.2020.104939 Medline
70. T. T. N. Thao, F. Labroussaa, N. Ebert, J. Jores, V. Thiel, In-Yeast Assembly of Coronavirus Infectious cDNA Clones Using a Synthetic Genomics Pipeline. *Methods Mol. Biol.* **2203**, 167–184 (2020). doi:10.1007/978-1-0716-0900-2_13 Medline
71. H.-H. Hoffmann, W. M. Schneider, K. Rozen-Gagnon, L. A. Miles, F. Schuster, B. Razooky, E. Jacobson, X. Wu, S. Yi, C. M. Rudin, M. R. MacDonald, L. K. McMullan, J. T. Poirier, C. M. Rice, TMEM41B Is a Pan-flavivirus Host Factor. *Cell* **184**, 133–148.e20 (2021). doi:10.1016/j.cell.2020.12.005 Medline
72. B. D. Lindenbach, M. J. Evans, A. J. Syder, B. Wölk, T. L. Tellinghuisen, C. C. Liu, T. Maruyama, R. O. Hynes, D. R. Burton, J. A. McKeating, C. M. Rice, Complete replication of hepatitis C virus in cell culture. *Science* **309**, 623–626 (2005). doi:10.1126/science.1114016 Medline
73. D. K. W. Chu, Y. Pan, S. M. S. Cheng, K. P. Y. Hui, P. Krishnan, Y. Liu, D. Y. M. Ng, C. K. C. Wan, P. Yang, Q. Wang, M. Peiris, L. L. M. Poon, Molecular Diagnosis of a Novel Coronavirus (2019-nCoV) Causing an Outbreak of Pneumonia. *Clin. Chem.* **66**, 549–555 (2020). doi:10.1093/clinchem/hvaa029 Medline

ACKNOWLEDGMENTS

We thank A. O'Connell, S.M. Pecoraro Di Vittorio, G. Santiago, M. E. Castillo, A. Webson, and S. Shirley for outstanding administrative or technical support. We thank F. Labroussaa, J. Jores, I. Minia and E. Wyler for technical advice, V. Larionov for TAR plasmid sequences, and M. C. Nussenzweig for neutralizing antibodies and helpful feedback. We thank the Rockefeller University Genomic Resource Center and High Throughput Facility cores, and the FACS Immunoassay and Digital PCR Instruments lab. **Funding:** The authors were supported for COVID-19 work with funding from the following awards, foundations, and charitable trusts: The G. Harold and Leila Y. Mathers Charitable Foundation (CMR), The Bawd Foundation (CMR), Fast Grants (www.fastgrants.org), a part of Emergent Ventures at the Mercatus Center, George Mason University (CMR), The Evergrande COVID-19 Response Fund Award from the Massachusetts Consortium on Pathogen Readiness, Swiss National Science Foundation grants 310030_173085 and 51NF40-182880 (VT), and National Institutes of Health administrative supplement to U19AI111825

(CMR). The authors were also supported with funding from the following awards, foundations, and charitable trusts: National Cancer Institute of the National Institutes of Health grant R01CA190261 (SWL), National Cancer Institute of the National Institutes of Health grant U01CA213359 (JTP), National Institute of Allergy and Infectious Diseases of the National Institutes of Health grants R01AI091707, R01AI143295, R01AI150275, R01AI124690, R01AI116943, P01AI138938 (CMR), The Robertson Foundation, the Center for Basic and Translational Research on Disorders of the Digestive System through the generosity of The Leona M. and Harry B. Helmsley Charitable Trust (CMR), National Institute of General Medical Sciences of the National Institutes of Health grant P41GM109824 (MPR), National Science Foundation grant NSF-1818129 (JFM), National Institute of Allergy and Infectious Diseases of the National Institutes of Health grants R03AI141855, R21AI142010 (MRM), Department of Defense award W81XWH1910409 (MRM), Bulgari Women & Science fellowship (IRL), EMBO Fellowship ALTF 380-2018 (JLP), HHMI Hanna Gray Fellowship (FJS-R), MSKCC Translational Research Oncology Training Fellowship NIH T32-CA160001 (FJS-R), Agilent Technologies Thought Leader Award (SWL), and Howard Hughes Medical Institute (SWL, PDB). **Author contributions:** Conceptualization: JML, IRL, TTNT, JLP, WMS, JTP, MRM, VT, CMR. Methodology: IRL, JML, TTNT, JLP, YY, HHH, WMS, JFM, BSR, FSR, NE. Formal analysis: JML, IRL. Investigation: IRL, JML, TTNT, JLP, YY, BT, IBV, BSR, KS. Resources: TTNT, YY, HHH, JFM, FS, YW, EM, BSR, FSR, SWL, MPR, TH, PDB, JTP, VT. Data curation: JML, IRL, JLP. Supervision: VT, CMR. Visualization: JML, IRL. Writing – original draft: IRL, JML, CMR. Writing – review & editing: TTNT, JLP, WMS, IBV, BSR, JFM, FSR, MPR, TH, JTP, MRM, VT, CMR. Project Administration: AP, MRM. Funding acquisition: SWL, JTP, VT, CMR. **Competing interests:** IRL, JML, JTP, VT, and CMR are inventors on a patent filed by The Rockefeller University, New York University, and the University of Bern based on the results described in this manuscript. CMR is a founder of Apath LLC, a Scientific Advisory Board member of Imvaq Therapeutics, Vir Biotechnology, and Arbutus Biopharma, and an advisor for Regulus Therapeutics and Pfizer. PDB has received compensation for consulting services to Pfizer. IRL is a paid consultant for Grit Bio. The remaining authors declare no competing interests. **Data and materials availability:** Data supporting the findings of this study are reported in the main text and figures and in supplementary figs. S1 to S4 and table S1. All reagents and materials generated in this study are available from the corresponding authors with a completed Materials Transfer Agreement to the Rockefeller University. Note that the biosafety containment level appropriate for use with these materials is subject to the policies of the requesting institution in addition to relevant regulatory bodies. This work is licensed under a Creative Commons Attribution 4.0 International (CC BY 4.0) license, which permits unrestricted use, distribution, and reproduction in any medium, provided the original work is properly cited. To view a copy of this license, visit https://creativecommons.org/licenses/by/4.0/. This license does not apply to figures/photos/artwork or other content included in the article that is credited to a third party; obtain authorization from the rights holder before using such material.

SUPPLEMENTARY MATERIALS

science.org/doi/10.1126/science.abj8430
Materials and Methods
Figs. S1 to S4
Table S1
References (*69*–*73*)
MDAR Reproducibility Checklist

5 June 2021; accepted 6 October 2021
Published online 14 October 2021
10.1126/science.abj8430

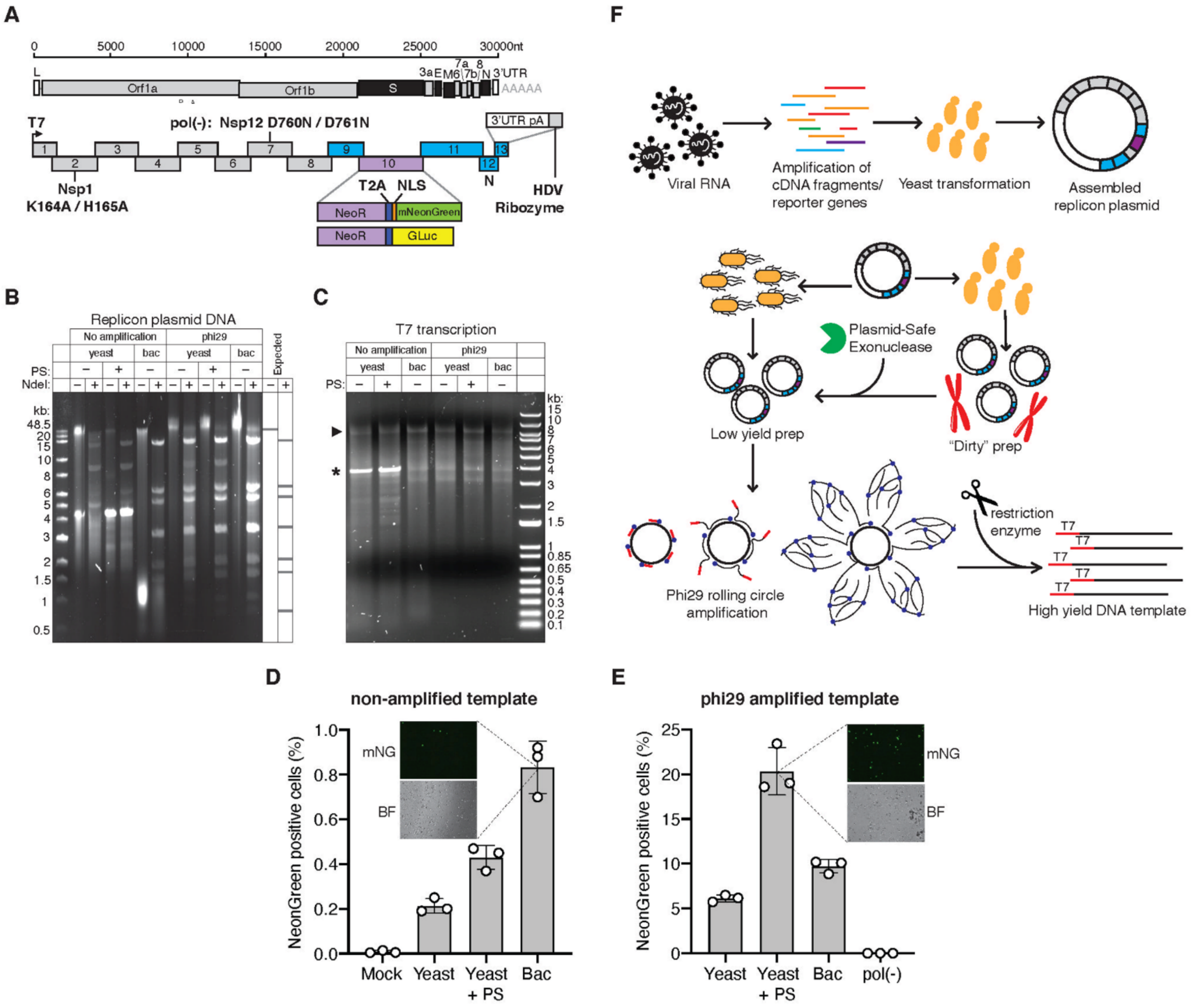

Fig. 1. SARS-CoV-2 replicon design and launch optimization. (**A**) Upper schematic: SARS-CoV-2 genome, with structural proteins in black. Lower schematic: Replicon amplicon fragments for yeast assembly. Fragments from (*10*) are shown in gray, fragments harboring mutations in Nsp1 or Nsp12 (pol-), are indicated. Reporter gene cassette in place of spike in purple; re-engineered flanking regions in blue. L: leader, UTR: untranslated region, pA: polyA, HDV: hepatitis delta virus, NLS: nuclear localization sequence. (**B**) Agarose gel of replicon DNA recovered from yeast or bacteria. Phi29 amplification or plasmid-Safe (PS) DNAse treatment indicated. Expected *Nde*I digest depicted at right. (**C**) Agarose gel of T7 RNA transcription reactions from DNA plasmids shown in (B). Arrow highlights the expected size of full-length RNA. Asterisk denotes truncated product. (**D** and **E**) Percent of mNeonGreen replicon positive BHK-21 cells from non-amplified (D) or phi29-amplified (E) DNA templates measured by flow cytometry. Insets show representative mNeonGreen (NG) and bright-field (BF) images. N = 3, error bars = SEM. Mock = no RNA electroporation. (**F**) Optimized RNA production for SARS-CoV-2 replicons. Overlapping PCR fragments are assembled in yeast and propagated in bacteria or yeast, in which case they are PS DNAse treated. Subsequent phi29 amplification ensures full-length DNA template availability for transcription.

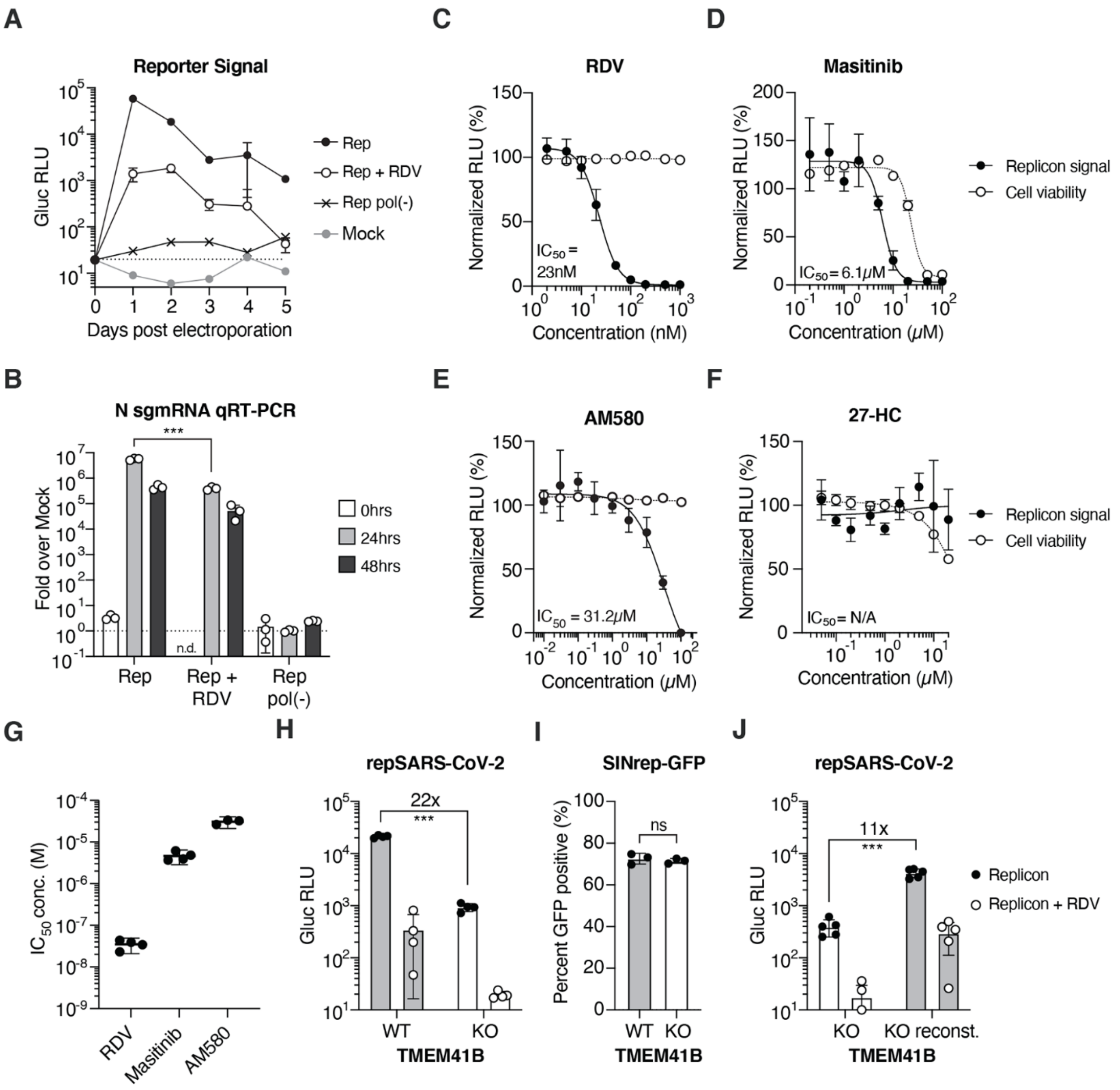

Fig. 2. SARS-CoV-2 replicons are sensitive to antiviral compounds, host factor loss, and viral mutant phenotypes. (**A**) *Gaussia* luciferase (Gluc) in Huh-7.5 supernatant from cells electroporated with Gluc replicon RNA, seeded with 100nM remdesivir (RDV) or vehicle. Mock electroporation and pol(-) replicons were used as controls. Dashed line indicates the limit of detection. N = 4. (**B**) qRT-PCR measurements for subgenomic N RNA for cells in (A). Signal from mock infected cells used for normalization (dashed line), N = 3. (**C** to **F**) Representative experiments in Huh-7.5 cells were electroporated with the Gluc replicon RNA and seeded with (C) remdesivir (N = 4), (D) masitinib (N = 4), (E) AM580 (N = 3), or (F) 27-hydroxycholesterol (27-HC) (N = 3). After 24 h, Gluc signal in the supernatant (black circles) and cell viability (empty circles) was measured and normalized to vehicle-treated cells, error bars = SEM. (**G**) IC_{50} values from independent experiments using the compounds presented in (C) to (E). (**H**) Parental Huh-7.5 (WT) and clonal TMEM41B KO cells were electroporated as in (A). Gluc was measured 24h post-electroporation, N = 4. (**I**) Cells as in (H) were electroporated with SinRep-GFP alphavirus replicon RNA. After 24 h, GFP-positive cells were quantified by flow cytometry, N = 3. (**J**) As in (H), using cells reconstituted with TMEM41B. N = 5. Error bars = SD, *** denotes P-value <0.001 using a two-sided Student's *t* test.

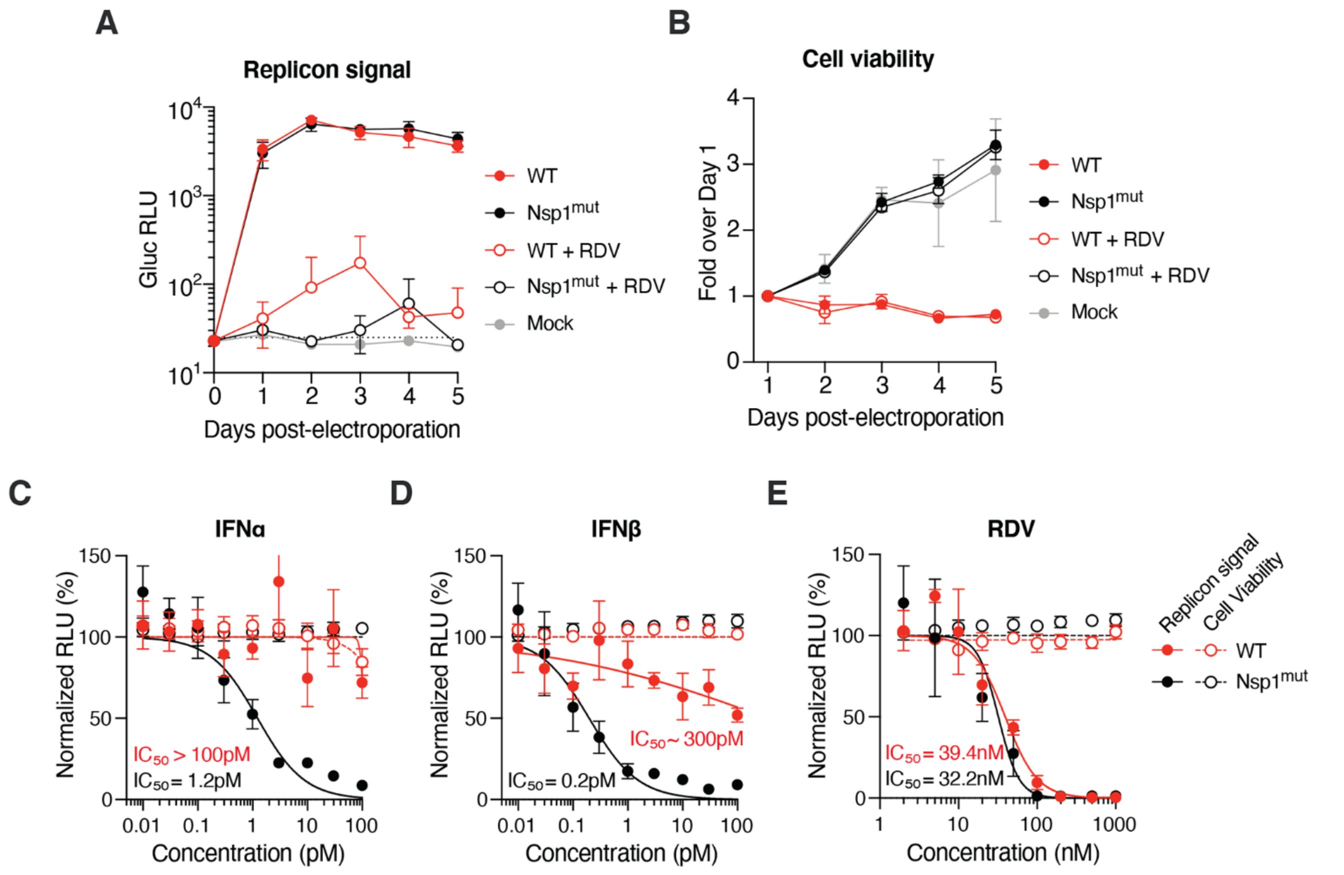

Fig. 3. Nsp1 deficient replicons are hypersensitive to interferons. (**A** and **B**) Timecourse measurements of Gluc in the supernatant (A) or cell viability (B) of Huh-7.5 cells electroporated with WT or Nsp1 K164A/H165A mutant (Nsp1^{mut}) Gluc replicon RNA. Cells were seeded with 100nM remdesivir (RDV) or vehicle and were washed in PBS 24 h prior to each respective timepoint collection. Mock-electroporated cells were used as controls for post-electroporation cell viability. The dashed line indicates the limit of detection. N = 4, error bars = SD. (**C** to **E**) Huh-7.5 were electroporated with WT or Nsp1^{mut} replicon RNA and seeded on 96-well plates containing indicated concentrations of (C) IFNα, (D) IFNβ, or (E) remdesivir (RDV). Gluc activity (closed circles) and cell viability (open circles) were measured 24 h post electroporation. N = 4, error bars = SD. IFN: interferon.

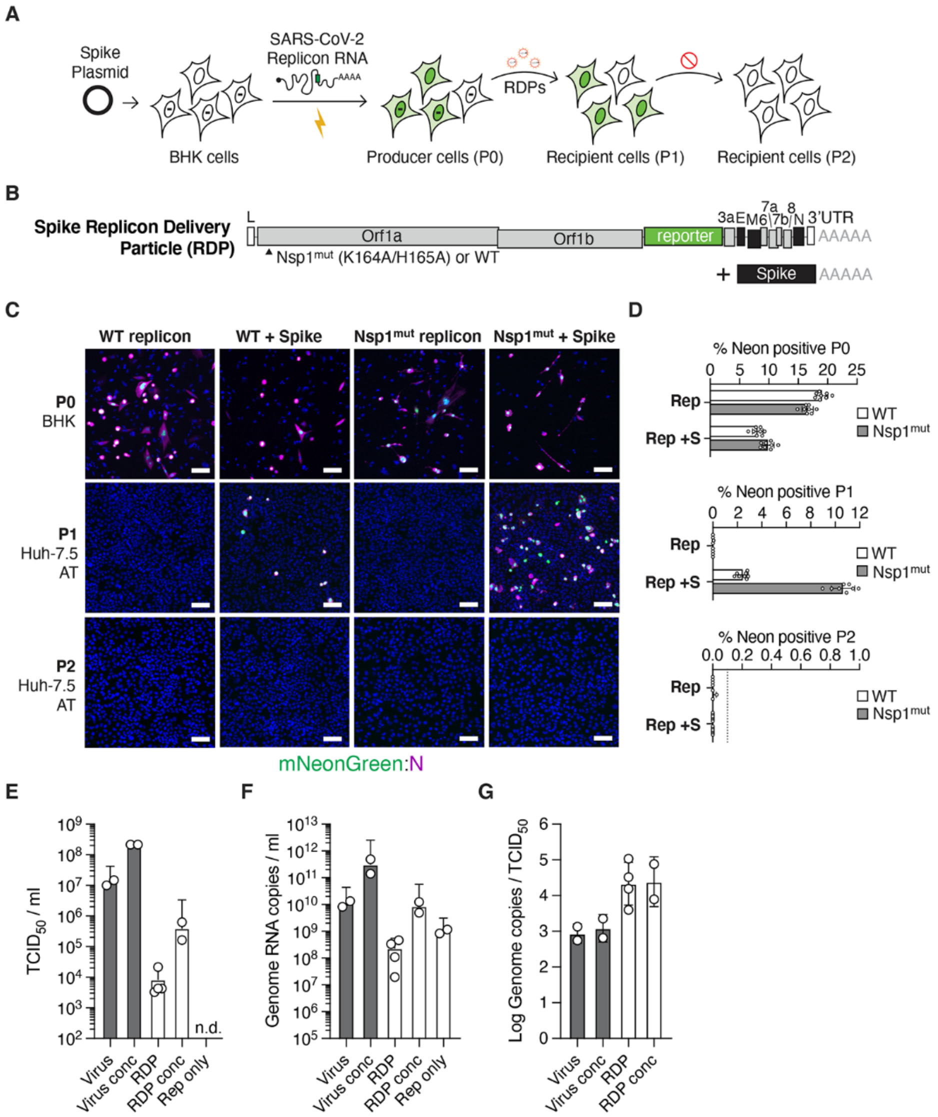

Fig. 4. Trans-complementation of replicons with spike yields single-cycle SARS-CoV-2. (A) A scheme to trans-complement replicons with ectopically expressed spike for single-cycle virion production. BHK-21 cells are transfected with a spike-encoding plasmid, and 24 h later are electroporated with ΔS mNeonGreen SARS-CoV-2 replicon RNA. Supernatant from these producer cells (P0) is collected and passed onto naïve recipient cells (P1) yielding reporter activity. A second round of passaging onto naïve recipient cells (P2) fails to propagate the replicon. (B) A spike trans-complemented replicon consists of spike-deleted replicon RNA alongside plasmid-driven spike expression. Nsp1 mutations relative to the WT sequence are indicated. (C) BHK-21 producer cells (P0) alone or transfected with a spike-encoding plasmid were electroporated with WT or $Nsp1^{mut}$ replicon RNAs. The RDPs in resulting supernatants were concentrated after 24 h and passaged onto Huh-7.5 ACE2/TMPRSS2 (Huh-7.5 AT) cells (P1 and P2) as in (A). Immunofluorescence images at 4X magnification of the mNeonGreen signal (green) and N antibody staining (magenta) are shown. Scale bar, 100 µm. (D) Quantification of the percentage of NeonGreen positive cells in each passage for the results in (C). The dashed lines denote the lower limit of quantification. N = 8, error bars = SD. (E) $TCID_{50}$/ml values of independently prepared SARS-CoV2 and RDP stocks were calculated by end-point dilution assay on Huh7.5 AT cells. Conc: stocks concentrated by PEG precipitation. n.d., not detected. (F) Genome RNA copies/ml from the virus and RDP stocks in (E) were calculated by qRT-PCR. (G) The ratio between RNA copies/ml in (F) and $TCID_{50}$/ml values in (E) was calculated to reflect specific infectivity.

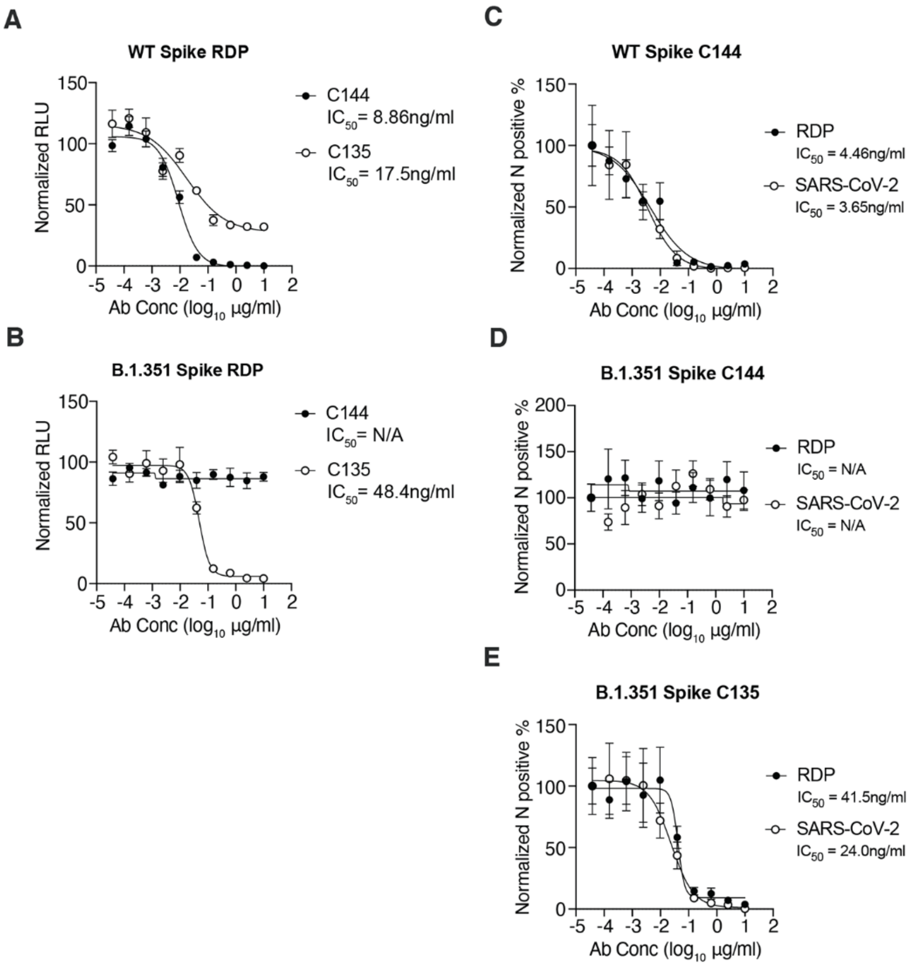

Fig. 5. Neutralization assays with RDPs recapitulate authentic SARS-CoV-2 antibody phenotypes. (**A** and **B**) Antibody neutralization assays in Huh-7.5 cells of Gluc RDPs trans-complemented with WA1/2020 spike (A) or the B.1.351 South African variant (B) in the presence of increasing concentrations of C144 and C135 neutralizing antibodies. Data are representative from two independent experiments and are normalized to infected cells without antibody. N = 3, error bars = SEM. IC_{50}, concentration resulting in 50% reduction in relative RLU values; NA, not applicable. (**C** to **E**) Neutralization assays for SARS-CoV-2 RDPs or virus in the presence of increasing concentrations of antibody. WT spike with C144 antibody (C), B.1.351 spike with C144 antibody (D) and B.1.351 with C135 antibody (E) are shown, representative from two independent experiments. Data are normalized to infected cells without antibody. N = 3, error bars = SEM. IC_{50}, concentration resulting in 50% relative reduction in RLU values; NA, not applicable.

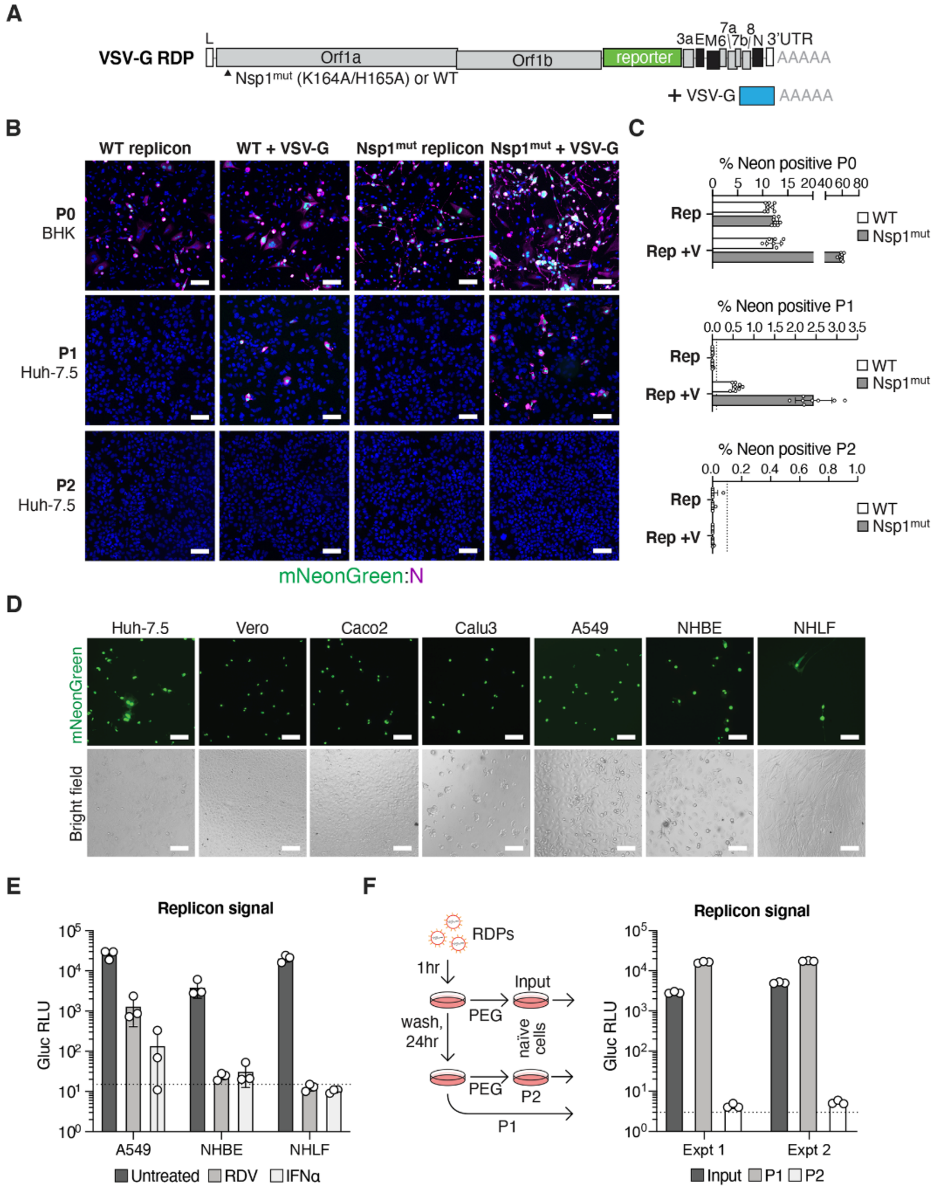

Fig. 6. VSV-G pseudotyping for efficient SARS-CoV-2 replicon delivery. (**A**) Schematic of the elements required for production of VSV-G RDPs. (**B**) BHK-21 producer cells (P0) alone or transfected with VSV-G were electroporated with WT or Nsp1^{mut} replicons. After 48 h, RDPs in supernatants were serially passaged onto Huh-7.5 cells (P1 and P2). mNeonGreen signal and N antibody staining depicted at 4X magnification. (**C**) Percentage of mNeonGreen positive cells in each passage from (B), N = 8, error bars = SD. (**D**) Indicated cell lines were incubated with mNeonGreen VSV-G RDPs for 24 h. Brightfield and fluorescent images were taken at 10X magnification. Scale bars, 100 μm. (**E**) NHBE, NHLF and A549 cells were pretreated for 24 h with 100nM of remdesivir (RDV) or 100pM of IFNα, and infected with Gluc VSV-G RDPs. Gluc activity was measured 24 h post-infection. N = 3, error bars = SD, dashed lines: signal from mock infected cells. (**F**) Single-cycle infectivity of VSV-G RDPs. Supernatant from RDP infected cells (P1) was read for Gluc activity and passaged onto naive cells (P2) after PEG concentration. Input supernatant serves as a positive control for concentrated input. Schema at left, results at right for two experiments.
