# Supplementary material for: Replication and single-cycle delivery of SARS-CoV-2 replicons
Source: Science. 2021 Oct 14;374(6571):1099–106. doi: 10.1126/science.abj8430 (PMC9007107; doi:10.1126/science.abj8430)
Supplement: Supplementary file 2 — Materials and Methods Figs. S1 to S4 References (69–73) [file science.abj8430_sm.pdf]

Science AAAS

Supplementary Materials for

Replication and single-cycle delivery of SARS-CoV-2 replicons

Inna Ricardo-Lax *et al.*

Corresponding authors: Volker Thiel, volker.thiel@vetsuisse.unibe.ch; Charles M. Rice, ricec@rockefeller.edu

Science **374**, 1099 (2021)
DOI: 10.1126/science.abj8430

The PDF file includes:

Materials and Methods
Figs. S1 to S4
References

Other Supplementary Material for this manuscript includes the following:

Table S1
MDAR Reproducibility Checklist

Materials and Methods

Replicon fragment cloning

Supplementary Table S1 contains all the primers used for fragment cloning. DNA fragments 2-6 and 8, inserted into pUC/pUC mini or the low-copy pCC1-BAC-HIS3 (in the case of fragment 5) were the same as described in (*10*). The rest of the fragments were PCR-amplified from SARS-Cov2 clone 3.1 ((*10*) except fragment 7, which was amplified from cDNA obtained by RT-PCR of viral RNA extracted from isolate USA-WA1/2020 (BEI Cat# NR-5281), grown in Vero-E6 cells. RNA was extracted from cells using Trizol (ThermoFisher #15596026) followed by RNeasy mini kit column purification (Qiagen Cat# 74104), and cDNA was prepared using SuperScript™ IV First-Strand Synthesis System with random primers (Thermo Cat# 18091050).

PCR amplification reactions for fragments 1, 9-13 were performed using KOD Xtreme Hot Start DNA Polymerase (EMD Millipore Cat# 71975). Reporter/selectable marker sequences, such as mNeonGreen, Gluc, NeoR, were PCR-amplified from plasmids or purchased as synthetic DNA (IDT). Amplicons of fragments 1, 9-13 were phosphorylated with T4 polynucleotide kinase (PNK) (NEB Cat# M0201) and ligated directly into a pGEM3Z plasmid amplified via PCR (T4 DNA ligase, NEB Cat# M0202T). Fragment 7, with or without viral RdRp mutations, was cloned into the backbone of low copy number plasmid pACNR (*69*). SARS-CoV-2 N protein was amplified by PCR to include a polyA sequence at the 3' end, and was cloned into *Kpn*I/*Xba*I digested pGEM3Z plasmid using Gibson assembly (NEB Cat# E2611) according to the manufacturer's protocol.

Fragments-containing pUC, pUC mini and pGEM3Z plasmids were grown in *E. coli* DH5α (ThermoFisher), pACNR and pCC1-BAC-His3 were grown in *E.coli* MC1061. Plasmid DNA was extracted with ZymoPURE II Plasmid Maxiprep (Zymo research Cat# D4202).

Mutagenesis of SARS-CoV-2 Nsp1 and Nsp12 was done using a two-step overlap PCR approach on fragments 2 and 7 respectively, using the primers listed in Table S1. The viral RdRp mutant was created by mutating Nsp12 catalytic residues at positions 760 and 761 from aspartic acid to asparagine (*32*). An Nsp1 mutant that does not bind the 40S ribosome was created by inserting K164A/H165A mutations (*39*, *40*). The mutated fragments were used for replicon assembly as detailed below.

The ΔAcc replicon was constructed using an alternative fragment 11 made via overlap PCR, which was designed to contain only E and M genes and their regulatory sequences, and none of the accessory genes. The primers for the EM-only fragment 11 are listed in Table S1.

Yeast assembly and bacterial propagation of assembled replicons

DNA fragments for assembly were prepared by restriction digestion or PCR as detailed in Table S1 and purified by agarose gel extraction. Yeast assembly was performed according to the protocol described in (*70*). Briefly, 50-100 ng of each fragment and the pCC1-BAC-His3 backbone were mixed together in an equimolar ratio and chemically transformed into *Saccharomyces cerevisiae* strain VL6-48N (ATCC Cat# MYA-3666). Transformed yeast cells were grown for 2-3 days on selective SD-HIS plates at 30ºC. Between 4-10 colonies from each plate were picked, re-streaked on a new selective plate and grown for 2 days at 30ºC. Crude DNA extraction was performed using glass beads (Biospec Cat# 11079110) and Chelex (SigmaAldrich Cat# 95577-100G-F) and

screened by multiplex PCR with the primers detailed in Table S1, using a multiplex PCR kit (Qiagen Cat# 206143) according to the manufacturer's protocol. Fragment 7, which contains the coding sequence for the viral RdRp, was amplified by PCR from positive clones and sequence-verified by sanger sequencing.

For large-scale plasmid extraction from yeast, the protocol in (*70*) was performed as described, however ZymoPURE II Plasmid Maxiprep (Zymo research Cat# D4202) kits were used in place of Qiagen kits.

To eliminate yeast chromosomal DNA, the plasmid preparation was digested with *Bam*HI-HF enzyme (NEB Cat# R3136T), for which no restriction sites are present in the replicon-containing plasmids. Next, DNA was digested with Plasmid-Safe™ ATP-Dependent DNase (Lucigen Cat# E3101K) for 24h at 37ºC and purified via extraction with Phenol-chloroform-isoamyl alcohol (SigmaAldrich Cat# 77617), followed by ethanol precipitation. Final DNA concentration was measured using Qbit with QuDye dsDNA HS Assay (ThermoFisher Cat# Q32851) following the manufacturer's instructions.

For bacterial propagation, 1µl of the crude yeast DNA prep was electroporated into *E. coli* TransforMax™ Epi300™ electrocompetent cells (Lucigen Cat# EC300110). The bacteria were then plated on chloramphenicol-containing plates and grown at 30°C overnight. Bacterial clones were used to make 5ml starter cultures and grown overnight at 30°C. The following day, the starter was diluted 1:10 in fresh Luria Broth (LB) media containing chloramphenicol with copy-control solution (Lucigen Cat# CCIS125), and incubated in a shaker incubator at 37ºC for 5h. Plasmids were then extracted using the Qiaprep spin miniprep kit (Qiagen Cat# 27104). As with the yeast preps, fragment 7 was amplified by PCR and the product sequence-verified by sanger sequencing.

Multiple displacement amplification (MDA) of replicon plasmids and DNA template preparation.

For amplification of pCC1-BAC-His3-replicon plasmids, 5-30ng of DNA was used with EquiPhi29™ DNA Polymerase (ThermoFisher Cat# A39390) according to the manufacturer's instructions, using exonuclease-resistant random primers (ThermoFisher Cat# SO181).

Amplified DNA was digested with *Not*I-HF enzyme (NEB #R3189S) and purified up with phenol-chloroform-isoamyl alcohol, followed by ethanol precipitation. The resulting DNA was sequence-verified by amplicon high-throughput sequencing at the Harvard MGH CCIB DNA Core Facility.

In vitro transcription of replicon RNA

Transcription reactions were set up with 1µg linear DNA templates using HiScribe™ T7 High Yield RNA Synthesis kit (NEB Cat# E2040S) or T7 RiboMAX™ Express Large Scale RNA Production System (Promega Cat# P1320) for 2h at 37ºC. After in-vitro transcription, RNA was treated with Ambion™ DNase I (ThermoFisher #AM2222) and cleaned using the Monarch® RNA Cleanup Kit (NEB Cat# T2050L). For co-transcriptional capping of RNA, Anti-Reverse Cap Analog (ARCA) was used at 1:2.8 (GTP:ARCA) ratio (NEB Cat# S1411S). For post-transcriptional capping, the ScriptCap Cap 1 Capping System (Cellscript Cat# C-SCCS1710**)** was used with the included ScriptCap 2'-O-Methyltransferase to generate Cap1 containing transcripts, or the ScriptCap 2'-O-Methyltransferase was omitted to generate Cap0 containing transcripts. Concentration and quality were assessed by nanodrop. To visualize the size of the RNA product, 1µl of finished transcription reactions were loaded with Gel Loading Buffer II (ThermoFisher Cat#

AM8546G) onto a 0.8% agarose gel stained with SYBR Safe (ThermoFisher, Cat# S33102). The gel was visualized on a 254nm UV light box or under EpiBlue light in a C600 gel imager (Azure Biosystems).

Sindbis replicon RNA was in-vitro transcribed from a SinRep5-GFP plasmid linearized with *Xho*I (NEB) (*2*), using the SP6 mMessage mMachine High Yield Capped RNA Transcription kit (ThermoFisher Cat# AM1340) according to the manufacturer's instructions.

Cell culture

Huh-7.5 cells (*H. sapiens*; sex: male; RRID:CVCL_7927) (*72*), Caco-2 (ATCC Cat# HTB-37, RRID:CVCL_0025, *H. sapiens*; sex: male), A549 (ATCC Cat# CCL-185, RRID:CVCL_0023, *H. sapiens*; sex: male) and VeroE6 cells (ATCC Cat# CRL-1586, RRID:CVCL_0574, *Chlorocebus sabaeus*) were maintained at 37ºC and 5% CO_2 in Dulbecco's Modified Eagle Medium (DMEM, Fisher Scientific #11995065) supplemented with 0.1 mM nonessential amino acids (NEAA, Fisher Scientific#11140076) and 10% hyclone fetal bovine serum (FBS, HyClone Laboratories, Lot. #AUJ35777). BHK-21 cells (ATCC Cat# CCL-10, RRID:CVCL_1915, *M. auratus*) were grown in Minimum Essential Medium (MEM, Fisher Scientific Cat# 11095080), supplemented with 8% FBS and 0.1 mM NEAA. Calu-3 cells (ATCC® HTB-55™, H. sapiens; sex: male) were grown in Eagle's Minimum Essential Medium (EMEM, ATCC Cat# 30-2003) supplemented with 10% FBS and 0.1 mM NEAA on collagen-coated dishes. Normal Human Lung Fibroblasts (NHLF, Lonza Cat# CC-2512) and Normal Human Bronchial Epithelial cells (NHBE, Lonza Cat# CC-2541) were subcultured using Reagent Pack™ Subculture Reagents (Lonza Cat# CC-5034) and maintained in FGM™-2 Fibroblast Growth Medium-2 BulletKit™ (Lonza Cat# CC-3132) and BEGM™-2 BulletKit™ Medium (Lonza Cat# CC-3170) respectively.

TMEM41B-KO and dox-inducible TMEM41B-reconstituted Huh-7.5 cells were previously described (*71*). TMEM41B expression was induced by 2µg/ml doxycycline (MilliporeSigma Cat# D9891) at least 24h before electroporation.

Huh-7.5 ACE2+TMPRSS2 cells were made by transduction with lentivirus bearing a TMPRSS2-2A-NeoR-ACE2 cassette (*38*) followed by selection with G418 (500µg/ml).

All cell lines tested negative for contamination with mycoplasma.

Electroporation of replicon RNA

RNA transcripts were electroporated into Huh-7.5 or BHK-21 cells using adapted protocols originally developed for launching HCVcc (*72*). Briefly, Huh-7.5 or BHK-21 cells were trypsinized, washed twice with ice-cold phosphate-buffered saline (PBS) (Invitrogen) and resuspended at 1.5 x 10^7 cells/ml in PBS. Subsequently, 5µg of SARS-CoV-2 replicon RNA and 2µg of SARS-CoV2 N mRNA were mixed with 0.4ml of cell suspension in a 2-mm cuvette (BTX #45-0125) and immediately pulsed using a BTX ElectroSquare Porator ECM 830 (860V, 99 µs, five pulses). Electroporated cells were incubated at room temperature for 10 min prior to resuspension in plating media.

Generation of spike-encoding plasmids

Spike matching the sequence of the USA-WA1/2020 isolate, was generated from the CDS of pSARS-CoV-2 (Sinobiological Cat# VG40589-UT) and subcloned into the *Nhe*I/*Xba*I linearized

vector pCR3.1 using Gibson assembly. To match the South African SARS-CoV2 isolate (hCoV-19/South Africa/KRISP-EC-K005321) the CDS of pSARS-CoV-2 VG40589-UT was used as a template to introduce equivalent spike substitutions D80A, D215G, K417N, E484K, N501Y, D614G, A701V and deletion of the positions 242-244 with a mutation in the furin cleavage site, R683G. The substitutions and deletion were introduced by fragment amplification, overlap extension PCR, and subsequent subcloning into the *Nhe*I/*Xba*I linearized vector pCR3.1 using Gibson assembly.

Spike and VSV-G trans-complementation for replicon delivery particle production

BHK-21 cells were transfected with spike- or VSV-G-encoding plasmids using Lipofectamine 3000 (ThermoFisher #L3000001), using a reverse-transfection protocol as per manufacturer's instructions (at 2µl reagent per ug DNA ratio). At 24h post transfection, 6 million cells were electroporated with 5µg replicon RNA and 2µg N protein mRNA as detailed above. Cells from one electroporation were seeded in a collagen-coated (Collagen from calf skin, Sigma #C8919) T75 flask or 3 electroporations were combined into a single T175 flask. For P0 imaging, some of the electroporated cells were seeded in parallel on collagen-coated 96-well plates for imaging. Supernatant was collected 24h post-electroporation for spike RDPs or 48h post-electroporation for VSV-G RDPs, and cleared by centrifugation. RDPs were concentrated via poly-ethylene glycol (PEG) precipitation, via an overnight incubation of the supernatant with a final concentration of 8% PEG6000 in 0.4M NaCl at 4°C. Subsequently, the PEG supernatant mixture was spun at 3000g for 30min. Pellets were resuspended in TNE Buffer (50mM Tris pH 7.4, 1mM EDTA, 200mM NaCl) and stored at -80°C until used.

Antiviral compounds

AM580 was purchased from Cayman Chemical (#15261), Remdesivir and Masitinib were purchased from MedChemExpress (#HY-104077 and #HY-10209 respectively), 27-hydroxycholesterol (27HC) was purchased from Sigma Aldrich (#SML2042), Human IFN Alpha A (Alpha 2a) and Human IFN Beta (1a) were purchased from PBL Assay Science (#11100-1 and #11410-2 respectively).

Secreted *Gaussia* luciferase assays

Following electroporation, between 15-30K cells in 100µl media per well were plated into 96-well plates containing equal volumes (100µl) of dilutions of the indicated compounds at 2x concentrations. Cell supernatants were cumulatively harvested at various timepoints or at 24hrs post-electroporation and luciferase signal was measured using the Renilla Luciferase Assay System (Promega, #E2820) in a Fluostar Omega microplate reader (BMG Labtech). Cell viability was measured using CellTiter-Glo® Luminescent Cell Viability Assay (Promega #G7570) following the manufacturer's instructions. For time-course experiments, cells were washed 3x with PBS 24h prior to luciferase harvest.

Viruses and virus titration

SARS-CoV-2, USA-WA1/2020 (#NR-52281) and South Africa/KRISP-EC-K005321/2020 Lineage B.1.351 (#NR-54008) were obtained from BEI resources. Viruses were amplified in VeroE6 and Huh-7.5 cells at 33 °C. Viral titers were measured on Huh-7.5 cells by standard plaque assay (PA). Briefly, 500 µL of serial 10-fold virus dilutions in Opti-MEM were used to infect

400,000 cells seeded the day prior in a 6-well plate format. After 90 min adsorption, the virus inoculum was removed, and cells were overlayed with DMEM containing 10% FBS with 1.2% microcrystalline cellulose (Avicel). Cells were incubated for five days at 33 °C, followed by fixation with 3.5% formaldehyde and crystal violet staining for plaque enumeration. For work in Huh7.5 AT cells, PA calculated Vero or Huh7.5 titers were used to perform serial dilution infectivity tests on Huh7.5 AT cells, where the dilution factor for either virus that resulted in approximately 40% of N positive cells 24h after infection was used for subsequent neutralization assays. For USA-WA1/2020, this dilution factor was 1:2000. For the B.1.351 virus variant, this dilution factor was 1:5000.

RDP $TCID_{50}$ measurements

To titrate spike or VSV-G RDPs, the relevant supernatants were used for end-point dilution assay by infecting Huh7.5 AT or Huh7.5 respectively. After 24h, the number of wells that had any positive signal (mNeonGreen or Gluc) were counted for each dilution, and $TCID_{50}$/ml values were calculated by the method of Spearman and Kärber, using the $TCID_{50}$ calculator (Marco Binder, Heidelberg University) accessible at https://www.klinikum.uni-heidelberg.de/fileadmin/inst_hygiene/molekulare_virologie/Downloads/TCID50_calculator_v2_17-01-20_MB.xlsx.

Neutralization Assays with SARS-CoV-2

The day prior to infection Huh-7.5 AT cells were seeded at 15,000 cells/well into 96-well plates in 100µl of plating media. Per well, human monoclonal antibodies C144 or C135 (a generous gift from the the lab of Michel Nussenzweig, The Rockefeller University) were serially diluted 4-fold in 50µl plating media. A fixed amount of SARS-CoV-2 (USA-WA1/2020 or B.1.351) based on the above determined dilution factors was mixed with antibody dilutions in equal volume (50µl) and incubated for 60 minutes at 37 °C. The 100µl antibody-virus-mix was then directly applied to Huh-7.5 AT cells. Cells were fixed 24h post infection in 2% formaldehyde for at least 20 minutes. Plates were subsequently processed for immunofluorescence of the N protein followed by a DAPI stain (see below). Percentage of N positive cells or foci were calculated per antibody dilution, and normalized to a no Ab control for quantification.

Neutralization Assays with RDPs

The day prior to infection Huh-7.5 AT cells were seeded at 15,000 cells/well into 96-well plates in 100µl of plating media. Human monoclonal antibodies C144 or C135 were 4-fold serially diluted in 50µl plating media and mixed with 700 $TCID_{50}$/well of USA-WA1/2020 (WT) spike RDPs or or B.1.351(South African) spike RDPs in 50µl media. The antibody-RDP mix was mixed 1:1 and incubated for 1 hour at 37 °C. The mix was then directly applied to the Huh-7.5 AT cells for an additional hour at 37 °C, followed by 4x washes with plating media. Cells were incubated in plating media at 37 °C. Supernatant was collected 24h later, and Gluc signal was read and normalized to a no Ab control. In parallel, cells were fixed with 2% PFA and stained for immunofluorescence of N protein as described below.

Immunofluorescence

For immunofluorescence experiments, between 15-30K cells in 100µl media per well were plated into black-walled clear bottom 96-well plates (Corning, Cat# 3904) following replicon

electroporation or in preparation for RDP addition. At time of harvest, cells were prepared by fixation with 2% paraformaldehyde (PFA) for at least 20 minutes. PFA was then removed and cells were stored at 4˚C in PBS containing 1% FBS until processing. Cells were washed in PBS containing 0.1% Tween-20 (PBST) and permeabilized with PBS containing 0.1% Triton X-100 for 10 min at room temperature. After washing with PBST, cells were incubated for 1h at room temperature with a blocking solution of 5% BSA in PBST. To stain SARS-CoV-2 infected cells, a rabbit polyclonal anti-SARS-CoV-2 nucleocapsid (N) antibody (GeneTex Cat# GTX135357, RRID:AB_2868464) was added to the cells at 1:1000-2000 dilution in PBST. VSV-G was stained with a mouse monoclonal anti-VSV-G antibody isolated from I1 hybridoma supernatant (ATCC Cat# CRL-2700, RRID:CVCL_G654) as described in (*22*) diluted 1:2,000 in PBST. SARS-CoV-2 spike was stained using the human C144 monoclonal antibody described in (*53*) and at 1:2,500 dilution in PBST. After overnight incubation at 4ºC, cells were washed and stained with secondary antibodies donkey anti-rabbit AlexaFluor 594 (ThermoFisher Scientific Cat# A-21207, RRID:AB_141637) at 1:2,000, donkey anti-mouse AlexaFluor 594 (Abcam Cat# ab150108, RRID:AB_2732073) at 1:2,000. Nuclei were counterstained with DAPI (ThermoFisher Scientific Cat# D1306, RRID:AB_2629482) at 1 µg/ml, for 5 minutes prior to imaging. For the results in Fig. 1 and Fig. 5D, fluorescent and brightfield images were taken with a Nikon eclipse TE300 fluorescent microscope at 10x magnification, using NIS-Elements 4.10.01 software (Nikon). For the results in Fig.s 4, 5, 6, S2, and S3, fluorescent images were obtained on a Keyence BZ-X710 microscope at 4x magnification, and quantified on Neon and antibody stained signals (N, VSV-G and/or spike) using the Hybrid Cell Count tool in the BZ-X Analyzer software (version 1.3.03). For virus and RDP area comparisons in Fig. S2, cell areas in pixels^2 were measured for both the DAPI channel or contiguous N signal in ImageJ and divided (N signal / DAPI) to achieve an N area per cell equivalent measure in arbitrary units.

qRT-PCR

Huh-7.5 cells containing Gluc SARS-CoV-2 replicons, or pol(-) replicons were seeded onto 12-well plates in triplicate at 1 x 10^5 cells/well and treated with 100nM remdesivir or DMSO vehicle. After incubating for 24 or 48h at 37ºC, supernatants were aspirated, cells were washed three times with PBS and subsequently lysed in 250 µl Tri-reagent (Zymo. Cat# R2050) per well. RNA was extracted using the Direct-zol RNA Miniprep Plus kit (Zymo Research Cat# R2072) according to the manufacturer's protocol, followed by reverse transcription into cDNA using random hexamer primers with the Superscript III First-Strand Synthesis System Kit (Invitrogen Cat# 18080051) also following the manufacturer's instructions. Gene expression was quantified by qRT-PCR using PowerUp SYBR Green Master Mix (Applied Biosystems Cat# A25742) and gene-specific primers for RPS11 (forward: 5'-GCCGAGACTATCTGCACTAC-3' and reverse: 5'-ATGTCCAGCCTCAGAACTTC-3') and SARS-CoV-2 subgenomic N (Leader forward: 5'-GTTTATACCTTCCCAGGTAACAAACC-3' and N reverse: 5'-GTAGAAATACCATCTTGGACTGAGATC-3'). SARS-CoV-2 primers targeting genomic N (forward: 5'-TAATCAGACAAGGAACTGATTA-3' and reverse: 5'-CGAAGGTGTGACTTCCATG-3') and Nsp14 (forward: 5'-TGGGGYTTTACRGGTAACCT-3' and reverse: 5'-AACRCGCTTAACAAAGCACTC-3') are from (*73*). The following PCR conditions were used: 50°C for 2 min and 95°C for 2 min (initial denaturation); 45 cycles of 95°C for 1 sec, 60°C for 30 sec (PCR), followed by 95°C for 15 sec, 65°C for 10 sec, and a slow increase to 95°C (0.07 °C/sec) for a melt curve. The data were analyzed by melt curve analysis for product specificity as well as ΔΔCT analysis for fold changes (after normalization to housekeeping genes)

and graphed using Prism 8 (GraphPad). A Ct value of 40 was used for samples that showed no detectable amplification (Mock samples) for the purposes of estimating a fold change. For genome copies / ml of superntant measurements, 200µl of RDP or virus supernatant, or 10µl of concentrated virus or RDP preps, was mixed with 800µl Trizol (ThermoFisher Cat# 15596026) and isolated following the manufacturers protocol, using Glycoblue (ThermoFisher Cat# AM9515) as a co-precipitant, and resuspended into 10µl water. The Luna Universal One-Step qRT-PCR kit (NEB Cat# E3005L) was used from a 1:1000 dilution of supernatant derived RNA per reaction. Standard curves were made from 10^6 to 1 copy per qPCR reaction of SARS-CoV-2 standard control RNAs (Twist Biosciences Cat #102019).

Flow cytometry

Cells were trypsinized, resuspended in PBS, and fixed with 2% paraformaldehyde (PFA) for at least 20 minutes. PFA was then removed and cells were resuspended in PBS containing 1%FBS. Flow cytometry was performed on a minimum of 10,000 single cells/sample using a LSRII Flow cytometer (BD Biosciences). Data analysis was performed with FloJo software (BD Biosciences).

Sample definition and statistical details

The experiments presented in this manuscript are representative results chosen from multiple independent experiments, defined here as originating from separate electroporations on different days, or from separate infections on different days. N values depicted in figures indicate independent biological replicates (separate wells) within a single representative experiment, with the exception of Figure 2G. Each experiment was repeated at least twice, with figure specific details as follows: Figure 1 electroporations, >5 repeats; Figure 2A-B, 2 repeats; Figure 2C-F, 3-4 repeats; Figure 2H-J, 2 repeats; Figure 3, 2 repeats; Figure 4C-D, 3 repeats; Figure 5, 2 repeats; Figure 6B-F, 2 repeats; Figures S2-S4, 2 repeats. Generation of plots and statistical analyses were performed using Prism8 (Graphpad). Error bars represent standard error of means or standard deviations, as indicated. We used Student's t-test (unpaired, two-tailed) to assess significance between treatment and control groups, and to calculate P values. $P < 0.05$ was considered statistically significant. We made no specific attempts to determine sample size, and any blinding, randomization or data attrition within an experiment was not carried out.

Biosafety considerations

While the work presented here was performed under Biological Safety Level 3 (BSL-3) conditions, the Institutional Biosafety Committee (IBC) of The Rockefeller University in coordination with the NIH Office of Science Policy, has granted investigator specific BSL-2 approval (to CMR) for the SARS-CoV-2 replicons and RDPs reported in this study. In this instance, the replicon data in Figures 1-2 and early RDP work demonstrating single cycle infectivity (specifically the experiment in Figure 6F) was presented to the Rockefeller IBC for guidance on whether this system would be appropriate for use at BSL-2. The determination was made that contacting the NIH Office of Science Policy was required, whereby we prepared materials outlining the construct details of the specific spike-deleted replicons reported in this manuscript, how they would be used to create RDPs using spike or VSV-G, how RDPs would be evaluated for single-cycle infectious activity, and an assessment on various means of replicon attenuation, such as the targeted use of loss-of-function mutants (e.g. Nsp1) and/or deletions of viral accessory or additional structural proteins. Based on this request and evaluation of the materials provided, this approval was granted

prior to journal submission. It is our intention that readers clearly understand the engineering controls and practices used in the performance of the experiments reported here. However, these considerations rest with each investigator, their Institutional Biosafety Committee, and their respective government regulatory body to determine appropriate biosafety practices for their experiments and we recognize the importance of standardized biosafety practices and the critical role that regulatory bodies play in defining safe laboratory operations for this virus.

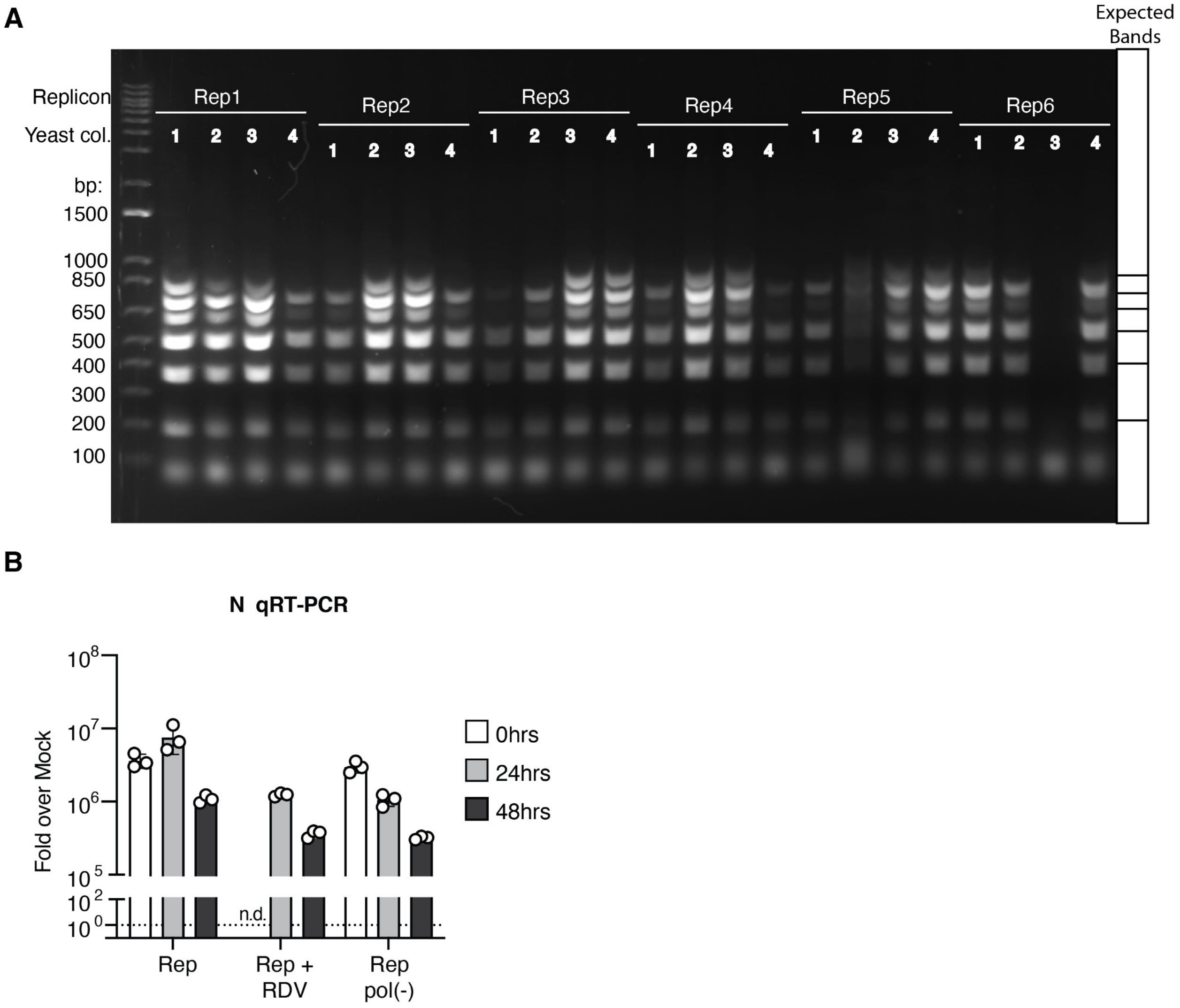

Fig. S1. Replicon assembly and RNA validation (Related to Fig. 1) (A) Representative multiplex PCR results for four clones each of six different replicons (Rep1-6) following assembly in yeast (see Methods). Expected PCR product sizes are indicated at right. **(B)** qRT-PCR measurements for N mRNA in Huh-7.5 cells 24h after electroporation with the 5µg Gluc replicon RNA plus 2µg N mRNA and seeding with 100nM remdesivir (RDV) or vehicle. Signal from mock electroporated cells was used for normalization (dashed line). N = 3, error bars = SD.

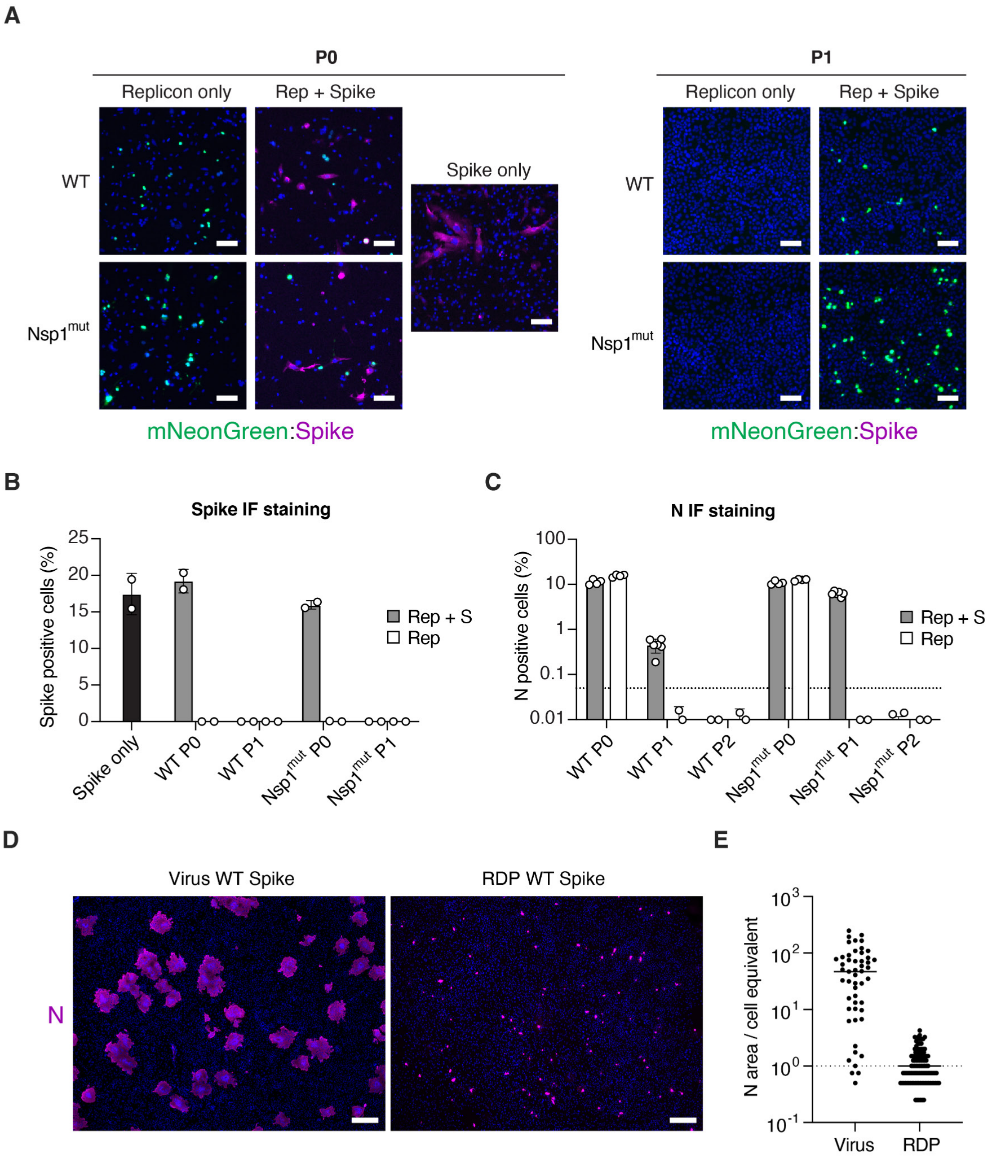

Fig. S2. Ectopic spike expression and quantification (Related to Fig. 4). (A) WT or Nsp1^{mut} replicon RNAs were electroporated into BHK-21 producer cells (P0) or into producer cells transfected with Spike-expressing plasmid and RDPs in the supernatants were subsequently passaged onto Huh-7.5 ACE2/TMPRSS2 (Huh-7.5 AT) cells (P1). Immunofluorescence images at 4X magnification of the mNeonGreen signal (green) and spike protein detected by C144 antibody staining (magenta) are shown. Scale bar, 100µm. **(B)** Quantification of the percentage of Spike positive cells at each passage for the results in **(A)**. N = 2, error bars = SD. **(C)** Quantification of the percentage of N positive cells at each passage for the results in Fig. 4C. N = 2-6, error bars = SD. **(D)** Immunofluorescence images showing N protein (magenta) in Huh-7.5 cells infected with SARS-CoV-2 (WA1/2020, left) or RDPs bearing WA1/2020 spike (right). Images were taken at 4X magnification, scale bar 500µm. **(E)** Quantification of results in **(D)**, estimating the per cell area for the N protein signal in virus or RDP positive cells. Area was normalized to the N signal from a single cell.

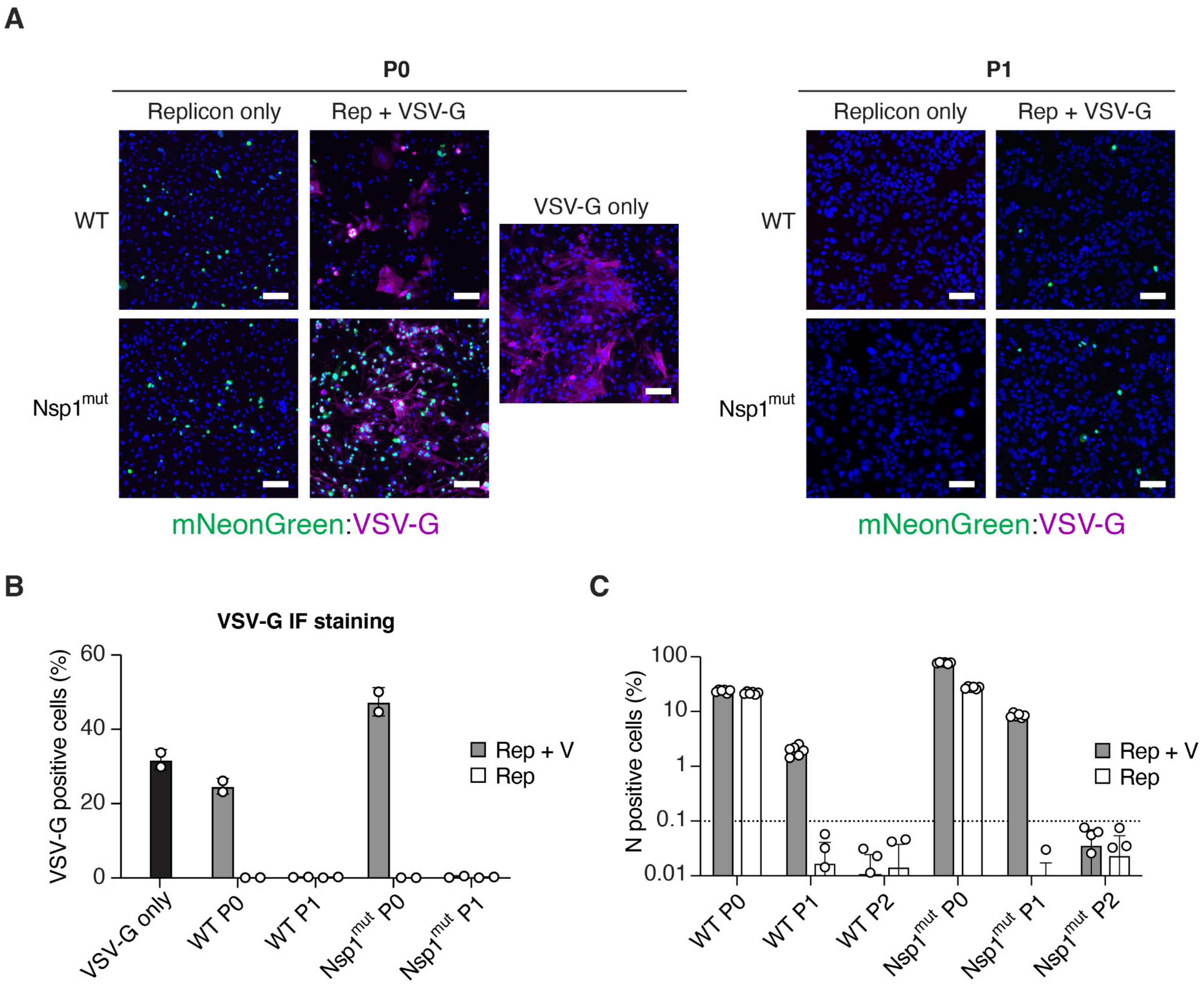

Fig. S3. Ectopic VSV-G expression and quantification (Related to Fig. 6). (A) WT or Nsp1^{mut} replicon RNAs were electroporated into BHK-21 producer cells (P0) or into producer cells transfected with VSV-G-expressing plasmid and supernatants were subsequently passaged onto Huh-7.5 cells (P1). Immunofluorescence images taken 24h later at 4X magnification of the mNeonGreen signal (green) and VSV-G antibody staining (magenta) are shown. Scale bar, 100µm. **(B)** Quantification of the percentage of VSV-G positive cells at each passage for the results in (A). N = 2, error bars = SD. **(C)** Quantification of the percentage of N positive cells at eachpassage for the results in Fig. 6C. N = 2-6, error bars = SD.

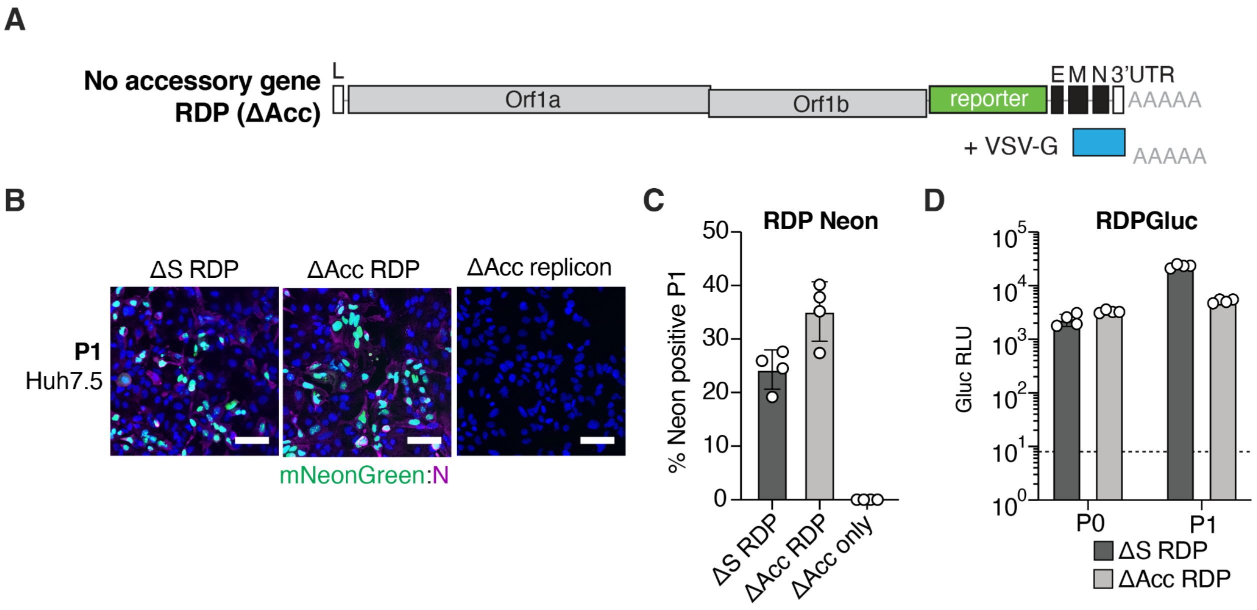

Fig. S4. VSV-G trans-complementation of a SARS-CoV-2 replicon lacking all accessory genes. (A) Schematic of the elements required for generating a VSV-G pseudotyped replicon lacking all accessory genes (ΔAcc). **(B)** VSV-G-pseudotyped ΔS or ΔAcc RDPs were concentrated and passaged onto Huh-7.5 cells (P1). Concentrated supernatant from ΔAcc electroporation alone was used as a negative control. Scale bar, 100µm. **(C)** Quantification of the percentage of NeonGreen positive cells for the results in (B). N = 4, error bars = SD. **(D)** VSV-G-pseudotyped ΔS or ΔAcc Gluc RDPs were made in producer BHK-21 cells (P0) and passaged onto Huh-7.5 cells (P1), and Gluc in the supernatants was measured after 48h/24h respectively. The dashed lines denote the lower limit of detection. N = 3, error bars = SD.

References and Notes

1. R. French, P. Ahlquist, Intercistronic as well as terminal sequences are required for efficient amplification of brome mosaic virus RNA3. *J. Virol.* **61**, 1457–1465 (1987). doi:10.1128/jvi.61.5.1457-1465.1987 Medline

2. P. J. Bredenbeek, I. Frolov, C. M. Rice, S. Schlesinger, Sindbis virus expression vectors: Packaging of RNA replicons by using defective helper RNAs. *J. Virol.* **67**, 6439–6446 (1993). doi:10.1128/jvi.67.11.6439-6446.1993 Medline

3. V. Lohmann, F. Körner, J. Koch, U. Herian, L. Theilmann, R. Bartenschlager, Replication of subgenomic hepatitis C virus RNAs in a hepatoma cell line. *Science* **285**, 110–113 (1999). doi:10.1126/science.285.5424.110 Medline

4. K. J. Blight, A. A. Kolykhalov, C. M. Rice, Efficient initiation of HCV RNA replication in cell culture. *Science* **290**, 1972–1974 (2000). doi:10.1126/science.290.5498.1972 Medline

5. V. Thiel, J. Herold, B. Schelle, S. G. Siddell, Viral replicase gene products suffice for coronavirus discontinuous transcription. *J. Virol.* **75**, 6676–6681 (2001). doi:10.1128/JVI.75.14.6676-6681.2001 Medline

6. K. M. Curtis, B. Yount, R. S. Baric, Heterologous gene expression from transmissible gastroenteritis virus replicon particles. *J. Virol.* **76**, 1422–1434 (2002). doi:10.1128/JVI.76.3.1422-1434.2002 Medline

7. G. Kaplan, V. R. Racaniello, Construction and characterization of poliovirus subgenomic replicons. *J. Virol.* **62**, 1687–1696 (1988). doi:10.1128/jvi.62.5.1687-1696.1988 Medline

8. H. Hannemann, Viral replicons as valuable tools for drug discovery. *Drug Discov. Today* **25**, 1026–1033 (2020). doi:10.1016/j.drudis.2020.03.010 Medline

9. V. Lohmann, Hepatitis C virus cell culture models: An encomium on basic research paving the road to therapy development. *Med. Microbiol. Immunol.* **208**, 3–24 (2019). doi:10.1007/s00430-018-0566-x Medline

10. T. Thi Nhu Thao, F. Labroussaa, N. Ebert, P. V'kovski, H. Stalder, J. Portmann, J. Kelly, S. Steiner, M. Holwerda, A. Kratzel, M. Gultom, K. Schmied, L. Laloli, L. Hüsser, M. Wider, S. Pfaender, D. Hirt, V. Cippà, S. Crespo-Pomar, S. Schröder, D. Muth, D. Niemeyer, V. M. Corman, M. A. Müller, C. Drosten, R. Dijkman, J. Jores, V. Thiel, Rapid reconstruction of SARS-CoV-2 using a synthetic genomics platform. *Nature* **582**, 561–565 (2020). doi:10.1038/s41586-020-2294-9 Medline

11. Y. J. Hou, K. Okuda, C. E. Edwards, D. R. Martinez, T. Asakura, K. H. Dinnon 3rd, T. Kato, R. E. Lee, B. L. Yount, T. M. Mascenik, G. Chen, K. N. Olivier, A. Ghio, L. V. Tse, S. R. Leist, L. E. Gralinski, A. Schäfer, H. Dang, R. Gilmore, S. Nakano, L. Sun, M. L. Fulcher, A. Livraghi-Butrico, N. I. Nicely, M. Cameron, C. Cameron, D. J. Kelvin, A. de Silva, D. M. Margolis, A. Markmann, L. Bartelt, R. Zumwalt, F. J. Martinez, S. P. Salvatore, A. Borczuk, P. R. Tata, V. Sontake, A. Kimple, I. Jaspers, W. K. O'Neal, S. H. Randell, R. C. Boucher, R. S. Baric, SARS-CoV-2 Reverse Genetics Reveals a Variable Infection Gradient in the Respiratory Tract. *Cell* **182**, 429–446.e14 (2020). doi:10.1016/j.cell.2020.05.042 Medline

12. S. Torii, C. Ono, R. Suzuki, Y. Morioka, I. Anzai, Y. Fauzyah, Y. Maeda, W. Kamitani, T. Fukuhara, Y. Matsuura, Establishment of a reverse genetics system for SARS-CoV-2 using circular polymerase extension reaction. *Cell Rep.* **35**, 109014 (2021). doi:10.1016/j.celrep.2021.109014 Medline

13. S. J. Rihn, A. Merits, S. Bakshi, M. L. Turnbull, A. Wickenhagen, A. J. T. Alexander, C. Baillie, B. Brennan, F. Brown, K. Brunker, S. R. Bryden, K. A. Burness, S. Carmichael, S. J. Cole, V. M. Cowton, P. Davies, C. Davis, G. De Lorenzo, C. L. Donald, M. Dorward, J. I. Dunlop, M. Elliott, M. Fares, A. da Silva Filipe, J. R. Freitas, W. Furnon, R. J. Gestuveo, A. Geyer, D. Giesel, D. M. Goldfarb, N. Goodman, R. Gunson, C. J. Hastie, V. Herder, J. Hughes, C. Johnson, N. Johnson, A. Kohl, K. Kerr, H. Leech, L. S. Lello, K. Li, G. Lieber, X. Liu, R. Lingala, C. Loney, D. Mair, M. J. McElwee, S. McFarlane, J. Nichols, K. Nomikou, A. Orr, R. J. Orton, M. Palmarini, Y. A. Parr, R. M. Pinto, S. Raggett, E. Reid, D. L. Robertson, J. Royle, N. Cameron-Ruiz, J. G. Shepherd, K. Smollett, D. G. Stewart, M. Stewart, E. Sugrue, A. M. Szemiel, A. Taggart, E. C. Thomson, L. Tong, L. S. Torrie, R. Toth, M. Varjak, S. Wang, S. G. Wilkinson, P. G. Wyatt, E. Zusinaite, D. R. Alessi, A. H. Patel, A. Zaid, S. J. Wilson, S. Mahalingam, A plasmid DNA-launched SARS-CoV-2 reverse genetics system and coronavirus toolkit for COVID-19 research. *PLOS Biol.* **19**, e3001091 (2021). doi:10.1371/journal.pbio.3001091 Medline

14. H. Xia, Z. Cao, X. Xie, X. Zhang, J. Y.-C. Chen, H. Wang, V. D. Menachery, R. Rajsbaum, P.-Y. Shi, Evasion of Type I Interferon by SARS-CoV-2. *Cell Rep.* **33**, 108234 (2020). doi:10.1016/j.celrep.2020.108234 Medline

15. Y. Zhang, W. Song, S. Chen, Z. Yuan, Z. Yi, A bacterial artificial chromosome (BAC)-vectored noninfectious replicon of SARS-CoV-2. *Antiviral Res.* **185**, 104974 (2021). doi:10.1016/j.antiviral.2020.104974 Medline

16. X. He, S. Quan, M. Xu, S. Rodriguez, S. L. Goh, J. Wei, A. Fridman, K. A. Koeplinger, S. S. Carroll, J. A. Grobler, A. S. Espeseth, D. B. Olsen, D. J. Hazuda, D. Wang, Generation of SARS-CoV-2 reporter replicon for high-throughput antiviral screening and testing. *Proc. Natl. Acad. Sci. U.S.A.* **118**, e2025866118 (2021). doi:10.1073/pnas.2025866118 Medline

17. Y. Luo, F. Yu, M. Zhou, Y. Liu, B. Xia, X. Zhang, J. Liu, J. Zhang, Y. Du, R. Li, L. Wu, X. Zhang, T. Pan, D. Guo, T. Peng, H. Zhang, Engineering a Reliable and Convenient SARS-CoV-2 Replicon System for Analysis of Viral RNA Synthesis and Screening of Antiviral Inhibitors. *mBio* **12**, e02754-20 (2021). doi:10.1128/mBio.02754-20 Medline

18. X. Ju, Y. Zhu, Y. Wang, J. Li, J. Zhang, M. Gong, W. Ren, S. Li, J. Zhong, L. Zhang, Q. C. Zhang, R. Zhang, Q. Ding, A novel cell culture system modeling the SARS-CoV-2 life cycle. *PLOS Pathog.* **17**, e1009439 (2021). doi:10.1371/journal.ppat.1009439 Medline

19. X. Zhang, Y. Liu, J. Liu, A. L. Bailey, K. S. Plante, J. A. Plante, J. Zou, H. Xia, N. E. Bopp, P. V. Aguilar, P. Ren, V. D. Menachery, M. S. Diamond, S. C. Weaver, X. Xie, P.-Y. Shi, A trans-complementation system for SARS-CoV-2 recapitulates authentic viral replication without virulence. *Cell* **184**, 2229–2238.e13 (2021). doi:10.1016/j.cell.2021.02.044 Medline

20. X. Ou, Y. Liu, X. Lei, P. Li, D. Mi, L. Ren, L. Guo, R. Guo, T. Chen, J. Hu, Z. Xiang, Z. Mu, X. Chen, J. Chen, K. Hu, Q. Jin, J. Wang, Z. Qian, Characterization of spike

glycoprotein of SARS-CoV-2 on virus entry and its immune cross-reactivity with SARS-CoV. *Nat. Commun.* **11**, 1620–12 (2020). doi:10.1038/s41467-020-15562-9 Medline

21. K. H. D. Crawford, R. Eguia, A. S. Dingens, A. N. Loes, K. D. Malone, C. R. Wolf, H. Y. Chu, M. A. Tortorici, D. Veesler, M. Murphy, D. Pettie, N. P. King, A. B. Balazs, J. D. Bloom, Protocol and Reagents for Pseudotyping Lentiviral Particles with SARS-CoV-2 Spike Protein for Neutralization Assays. *Viruses* **12**, 513 (2020). doi:10.3390/v12050513 Medline

22. F. Schmidt, Y. Weisblum, F. Muecksch, H.-H. Hoffmann, E. Michailidis, J. C. C. Lorenzi, P. Mendoza, M. Rutkowska, E. Bednarski, C. Gaebler, M. Agudelo, A. Cho, Z. Wang, A. Gazumyan, M. Cipolla, M. Caskey, D. F. Robbiani, M. C. Nussenzweig, C. M. Rice, T. Hatziioannou, P. D. Bieniasz, Measuring SARS-CoV-2 neutralizing antibody activity using pseudotyped and chimeric viruses. *J. Exp. Med.* **217**, e20201181 (2020). doi:10.1084/jem.20201181 Medline

23. J. Nie, Q. Li, J. Wu, C. Zhao, H. Hao, H. Liu, L. Zhang, L. Nie, H. Qin, M. Wang, Q. Lu, X. Li, Q. Sun, J. Liu, C. Fan, W. Huang, M. Xu, Y. Wang, Establishment and validation of a pseudovirus neutralization assay for SARS-CoV-2. *Emerg. Microbes Infect.* **9**, 680–686 (2020). doi:10.1080/22221751.2020.1743767 Medline

24. M. Chen, X.-E. Zhang, Construction and applications of SARS-CoV-2 pseudoviruses: A mini review. *Int. J. Biol. Sci.* **17**, 1574–1580 (2021). doi:10.7150/ijbs.59184 Medline

25. A. Wu, Y. Peng, B. Huang, X. Ding, X. Wang, P. Niu, J. Meng, Z. Zhu, Z. Zhang, J. Wang, J. Sheng, L. Quan, Z. Xia, W. Tan, G. Cheng, T. Jiang, Genome Composition and Divergence of the Novel Coronavirus (2019-nCoV) Originating in China. *Cell Host Microbe* **27**, 325–328 (2020). doi:10.1016/j.chom.2020.02.001 Medline

26. I. Jungreis, R. Sealfon, M. Kellis, SARS-CoV-2 gene content and COVID-19 mutation impact by comparing 44 Sarbecovirus genomes. *Nat. Commun.* **12**, 2642–20 (2021). doi:10.1038/s41467-021-22905-7 Medline

27. N. Kouprina, V. Larionov, Transformation-associated recombination (TAR) cloning for genomics studies and synthetic biology. *Chromosoma* **125**, 621–632 (2016). doi:10.1007/s00412-016-0588-3 Medline

28. L. Blanco, A. Bernad, J. M. Lázaro, G. Martín, C. Garmendia, M. Salas, Highly efficient DNA synthesis by the phage phi 29 DNA polymerase. Symmetrical mode of DNA replication. *J. Biol. Chem.* **264**, 8935–8940 (1989). doi:10.1016/S0021-9258(18)81883-X Medline

29. F. B. Dean, J. R. Nelson, T. L. Giesler, R. S. Lasken, Rapid amplification of plasmid and phage DNA using Phi 29 DNA polymerase and multiply-primed rolling circle amplification. *Genome Res.* **11**, 1095–1099 (2001). doi:10.1101/gr.180501 Medline

30. J. A. Esteban, M. Salas, L. Blanco, Fidelity of phi 29 DNA polymerase. Comparison between protein-primed initiation and DNA polymerization. *J. Biol. Chem.* **268**, 2719–2726 (1993). doi:10.1016/S0021-9258(18)53833-3 Medline

31. J. Banér, M. Nilsson, M. Mendel-Hartvig, U. Landegren, Signal amplification of padlock probes by rolling circle replication. *Nucleic Acids Res.* **26**, 5073–5078 (1998). doi:10.1093/nar/26.22.5073 Medline

32. Y. Gao, L. Yan, Y. Huang, F. Liu, Y. Zhao, L. Cao, T. Wang, Q. Sun, Z. Ming, L. Zhang, J. Ge, L. Zheng, Y. Zhang, H. Wang, Y. Zhu, C. Zhu, T. Hu, T. Hua, B. Zhang, X. Yang, J. Li, H. Yang, Z. Liu, W. Xu, L. W. Guddat, Q. Wang, Z. Lou, Z. Rao, Structure of the RNA-dependent RNA polymerase from COVID-19 virus. *Science* **368**, 779–782 (2020). doi:10.1126/science.abb7498 Medline

33. C. J. Gordon, E. P. Tchesnokov, E. Woolner, J. K. Perry, J. Y. Feng, D. P. Porter, M. Götte, Remdesivir is a direct-acting antiviral that inhibits RNA-dependent RNA polymerase from severe acute respiratory syndrome coronavirus 2 with high potency. *J. Biol. Chem.* **295**, 6785–6797 (2020). doi:10.1074/jbc.RA120.013679 Medline

34. A. J. Pruijssers, A. S. George, A. Schäfer, S. R. Leist, L. E. Gralinksi, K. H. Dinnon 3rd, B. L. Yount, M. L. Agostini, L. J. Stevens, J. D. Chappell, X. Lu, T. M. Hughes, K. Gully, D. R. Martinez, A. J. Brown, R. L. Graham, J. K. Perry, V. Du Pont, J. Pitts, B. Ma, D. Babusis, E. Murakami, J. Y. Feng, J. P. Bilello, D. P. Porter, T. Cihlar, R. S. Baric, M. R. Denison, T. P. Sheahan, Remdesivir Inhibits SARS-CoV-2 in Human Lung Cells and Chimeric SARS-CoV Expressing the SARS-CoV-2 RNA Polymerase in Mice. *Cell Rep.* **32**, 107940 (2020). doi:10.1016/j.celrep.2020.107940 Medline

35. N. Drayman *et al.*, Drug repurposing screen identifies masitinib as a 3CLpro inhibitor that blocks replication of SARS-CoV-2 in vitro. bioRxiv 2020.08.31.274639 (2020); https://doi.org/10.1101/2020.08.31.274639.

36. A. Marcello, A. Civra, R. Milan Bonotto, L. Nascimento Alves, S. Rajasekharan, C. Giacobone, C. Caccia, R. Cavalli, M. Adami, P. Brambilla, D. Lembo, G. Poli, V. Leoni, The cholesterol metabolite 27-hydroxycholesterol inhibits SARS-CoV-2 and is markedly decreased in COVID-19 patients. *Redox Biol.* **36**, 101682 (2020). doi:10.1016/j.redox.2020.101682 Medline

37. S. Yuan, H. Chu, J. F.-W. Chan, Z.-W. Ye, L. Wen, B. Yan, P.-M. Lai, K.-M. Tee, J. Huang, D. Chen, C. Li, X. Zhao, D. Yang, M. C. Chiu, C. Yip, V. K.-M. Poon, C. C.-S. Chan, K.-H. Sze, J. Zhou, I. H.-Y. Chan, K.-H. Kok, K. K.-W. To, R. Y.-T. Kao, J. Y.-N. Lau, D.-Y. Jin, S. Perlman, K.-Y. Yuen, SREBP-dependent lipidomic reprogramming as a broad-spectrum antiviral target. *Nat. Commun.* **10**, 120–15 (2019). doi:10.1038/s41467-018-08015-x Medline

38. W. M. Schneider, J. M. Luna, H.-H. Hoffmann, F. J. Sánchez-Rivera, A. A. Leal, A. W. Ashbrook, J. Le Pen, I. Ricardo-Lax, E. Michailidis, A. Peace, A. F. Stenzel, S. W. Lowe, M. R. MacDonald, C. M. Rice, J. T. Poirier, Genome-Scale Identification of SARS-CoV-2 and Pan-coronavirus Host Factor Networks. *Cell* **184**, 120–132.e14 (2021). doi:10.1016/j.cell.2020.12.006 Medline

39. M. Thoms, R. Buschauer, M. Ameismeier, L. Koepke, T. Denk, M. Hirschenberger, H. Kratzat, M. Hayn, T. Mackens-Kiani, J. Cheng, J. H. Straub, C. M. Stürzel, T. Fröhlich, O. Berninghausen, T. Becker, F. Kirchhoff, K. M. J. Sparrer, R. Beckmann, Structural basis for translational shutdown and immune evasion by the Nsp1 protein of SARS-CoV-2. *Science* **369**, 1249–1255 (2020). doi:10.1126/science.abc8665 Medline

40. K. Schubert, E. D. Karousis, A. Jomaa, A. Scaiola, B. Echeverria, L.-A. Gurzeler, M. Leibundgut, V. Thiel, O. Mühlemann, N. Ban, SARS-CoV-2 Nsp1 binds the ribosomal mRNA channel to inhibit translation. *Nat. Struct. Mol. Biol.* **27**, 959–966 (2020). doi:10.1038/s41594-020-0511-8 Medline

41. C. P. Lapointe, R. Grosely, A. G. Johnson, J. Wang, I. S. Fernández, J. D. Puglisi, Dynamic competition between SARS-CoV-2 NSP1 and mRNA on the human ribosome inhibits translation initiation. *Proc. Natl. Acad. Sci. U.S.A.* **118**, e2017715118 (2021). doi:10.1073/pnas.2017715118 Medline

42. A. Tidu, A. Janvier, L. Schaeffer, P. Sosnowski, L. Kuhn, P. Hammann, E. Westhof, G. Eriani, F. Martin, The viral protein NSP1 acts as a ribosome gatekeeper for shutting down host translation and fostering SARS-CoV-2 translation. *RNA* **27**, 253–264 (2020). doi:10.1261/rna.078121.120 Medline

43. A. K. Banerjee, M. R. Blanco, E. A. Bruce, D. D. Honson, L. M. Chen, A. Chow, P. Bhat, N. Ollikainen, S. A. Quinodoz, C. Loney, J. Thai, Z. D. Miller, A. E. Lin, M. M. Schmidt, D. G. Stewart, D. Goldfarb, G. De Lorenzo, S. J. Rihn, R. M. Voorhees, J. W. Botten, D. Majumdar, M. Guttman, SARS-CoV-2 Disrupts Splicing, Translation, and Protein Trafficking to Suppress Host Defenses. *Cell* **183**, 1325–1339.e21 (2020). doi:10.1016/j.cell.2020.10.004 Medline

44. Y. Finkel, A. Gluck, A. Nachshon, R. Winkler, T. Fisher, B. Rozman, O. Mizrahi, Y. Lubelsky, B. Zuckerman, B. Slobodin, Y. Yahalom-Ronen, H. Tamir, I. Ulitsky, T. Israely, N. Paran, M. Schwartz, N. Stern-Ginossar, SARS-CoV-2 uses a multipronged strategy to impede host protein synthesis. *Nature* **594**, 240–245 (2021). doi:10.1038/s41586-021-03610-3 Medline

45. K. G. Lokugamage, K. Narayanan, C. Huang, S. Makino, Severe acute respiratory syndrome coronavirus protein nsp1 is a novel eukaryotic translation inhibitor that represses multiple steps of translation initiation. *J. Virol.* **86**, 13598–13608 (2012). doi:10.1128/JVI.01958-12 Medline

46. W. Kamitani, C. Huang, K. Narayanan, K. G. Lokugamage, S. Makino, A two-pronged strategy to suppress host protein synthesis by SARS coronavirus Nsp1 protein. *Nat. Struct. Mol. Biol.* **16**, 1134–1140 (2009). doi:10.1038/nsmb.1680 Medline

47. K. Narayanan, C. Huang, K. Lokugamage, W. Kamitani, T. Ikegami, C.-T. K. Tseng, S. Makino, Severe acute respiratory syndrome coronavirus nsp1 suppresses host gene expression, including that of type I interferon, in infected cells. *J. Virol.* **82**, 4471–4479 (2008). doi:10.1128/JVI.02472-07 Medline

48. X. Lei, X. Dong, R. Ma, W. Wang, X. Xiao, Z. Tian, C. Wang, Y. Wang, L. Li, L. Ren, F. Guo, Z. Zhao, Z. Zhou, Z. Xiang, J. Wang, Activation and evasion of type I interferon responses by SARS-CoV-2. *Nat. Commun.* **11**, 3810–3812 (2020). doi:10.1038/s41467-020-17665-9 Medline

49. T. Hertzig, E. Scandella, B. Schelle, J. Ziebuhr, S. G. Siddell, B. Ludewig, V. Thiel, Rapid identification of coronavirus replicase inhibitors using a selectable replicon RNA. *J. Gen. Virol.* **85**, 1717–1725 (2004). doi:10.1099/vir.0.80044-0 Medline

50. R. Sumpter Jr., Y.-M. Loo, E. Foy, K. Li, M. Yoneyama, T. Fujita, S. M. Lemon, M. Gale Jr., Regulating intracellular antiviral defense and permissiveness to hepatitis C virus RNA replication through a cellular RNA helicase, RIG-I. *J. Virol.* **79**, 2689–2699 (2005). doi:10.1128/JVI.79.5.2689-2699.2005 Medline

51. M. Habjan, N. Penski, M. Spiegel, F. Weber, T7 RNA polymerase-dependent and -independent systems for cDNA-based rescue of Rift Valley fever virus. *J. Gen. Virol.* **89**, 2157–2166 (2008). doi:10.1099/vir.0.2008/002097-0 Medline

52. D. Dufour, P. A. Mateos-Gomez, L. Enjuanes, J. Gallego, I. Sola, Structure and functional relevance of a transcription-regulating sequence involved in coronavirus discontinuous RNA synthesis. *J. Virol.* **85**, 4963–4973 (2011). doi:10.1128/JVI.02317-10 Medline

53. I. Sola, F. Almazán, S. Zúñiga, L. Enjuanes, Continuous and Discontinuous RNA Synthesis in Coronaviruses. *Annu. Rev. Virol.* **2**, 265–288 (2015). doi:10.1146/annurev-virology-100114-055218 Medline

54. H. Tegally, E. Wilkinson, M. Giovanetti, A. Iranzadeh, V. Fonseca, J. Giandhari, D. Doolabh, S. Pillay, E. J. San, N. Msomi, K. Mlisana, A. von Gottberg, S. Walaza, M. Allam, A. Ismail, T. Mohale, A. J. Glass, S. Engelbrecht, G. Van Zyl, W. Preiser, F. Petruccione, A. Sigal, D. Hardie, G. Marais, N. Y. Hsiao, S. Korsman, M.-A. Davies, L. Tyers, I. Mudau, D. York, C. Maslo, D. Goedhals, S. Abrahams, O. Laguda-Akingba, A. Alisoltani-Dehkordi, A. Godzik, C. K. Wibmer, B. T. Sewell, J. Lourenço, L. C. J. Alcantara, S. L. Kosakovsky Pond, S. Weaver, D. Martin, R. J. Lessells, J. N. Bhiman, C. Williamson, T. de Oliveira, Detection of a SARS-CoV-2 variant of concern in South Africa. *Nature* **592**, 438–443 (2021). doi:10.1038/s41586-021-03402-9 Medline

55. D. F. Robbiani, C. Gaebler, F. Muecksch, J. C. C. Lorenzi, Z. Wang, A. Cho, M. Agudelo, C. O. Barnes, A. Gazumyan, S. Finkin, T. Hägglöf, T. Y. Oliveira, C. Viant, A. Hurley, H.-H. Hoffmann, K. G. Millard, R. G. Kost, M. Cipolla, K. Gordon, F. Bianchini, S. T. Chen, V. Ramos, R. Patel, J. Dizon, I. Shimeliovich, P. Mendoza, H. Hartweger, L. Nogueira, M. Pack, J. Horowitz, F. Schmidt, Y. Weisblum, E. Michailidis, A. W. Ashbrook, E. Waltari, J. E. Pak, K. E. Huey-Tubman, N. Koranda, P. R. Hoffman, A. P. West Jr., C. M. Rice, T. Hatziioannou, P. J. Bjorkman, P. D. Bieniasz, M. Caskey, M. C. Nussenzweig, Convergent antibody responses to SARS-CoV-2 in convalescent individuals. *Nature* **584**, 437–442 (2020). doi:10.1038/s41586-020-2456-9 Medline

56. Y. Weisblum, F. Schmidt, F. Zhang, J. DaSilva, D. Poston, J. C. Lorenzi, F. Muecksch, M. Rutkowska, H. H. Hoffmann, E. Michailidis, C. Gaebler, M. Agudelo, A. Cho, Z. Wang, A. Gazumyan, M. Cipolla, L. Luchsinger, C. D. Hillyer, M. Caskey, D. F. Robbiani, C. M. Rice, M. C. Nussenzweig, T. Hatziioannou, P. D. Bieniasz, Escape from neutralizing antibodies by SARS-CoV-2 spike protein variants. *eLife* **9**, e61312 (2020). doi:10.7554/eLife.61312 Medline

57. D.-Y. Chen *et al.*, SARS-CoV-2 desensitizes host cells to interferon through inhibition of the JAK-STAT pathway. bioRxiv 2020.10.27.358259 (2020); https://doi.org/10.1101/2020.10.27.358259.

58. J. Cronin, X.-Y. Zhang, J. Reiser, Altering the tropism of lentiviral vectors through pseudotyping. *Curr. Gene Ther.* **5**, 387–398 (2005). doi:10.2174/1566523054546224 Medline

59. K. Verhoeckx *et al.*, Caco-2 Cell Line. *The Impact of Food Bioactives on Health* (2015), 175, 103–111.

60. M. Letko, A. Marzi, V. Munster, Functional assessment of cell entry and receptor usage for SARS-CoV-2 and other lineage B betacoronaviruses. *Nat. Microbiol.* **5**, 562–569 (2020). doi:10.1038/s41564-020-0688-y Medline

61. Y. Zhu, A. Chidekel, T. H. Shaffer, Cultured human airway epithelial cells (calu-3): A model of human respiratory function, structure, and inflammatory responses. *Crit. Care Res. Pract.* **2010**, 1–8 (2010). doi:10.1155/2010/394578 Medline

62. V. Cagno, SARS-CoV-2 cellular tropism. *Lancet Microbe* **1**, e2–e3 (2020). doi:10.1016/S2666-5247(20)30008-2 Medline

63. D. J. Giard, S. A. Aaronson, G. J. Todaro, P. Arnstein, J. H. Kersey, H. Dosik, W. P. Parks, In vitro cultivation of human tumors: Establishment of cell lines derived from a series of solid tumors. *J. Natl. Cancer Inst.* **51**, 1417–1423 (1973). doi:10.1093/jnci/51.5.1417 Medline

64. D. Blanco-Melo, B. E. Nilsson-Payant, W.-C. Liu, S. Uhl, D. Hoagland, R. Møller, T. X. Jordan, K. Oishi, M. Panis, D. Sachs, T. T. Wang, R. E. Schwartz, J. K. Lim, R. A. Albrecht, B. R. tenOever, Imbalanced Host Response to SARS-CoV-2 Drives Development of COVID-19. *Cell* **181**, 1036–1045.e9 (2020). doi:10.1016/j.cell.2020.04.026 Medline

65. Y. L. Siu, K. T. Teoh, J. Lo, C. M. Chan, F. Kien, N. Escriou, S. W. Tsao, J. M. Nicholls, R. Altmeyer, J. S. M. Peiris, R. Bruzzone, B. Nal, The M, E, and N structural proteins of the severe acute respiratory syndrome coronavirus are required for efficient assembly, trafficking, and release of virus-like particles. *J. Virol.* **82**, 11318–11330 (2008). doi:10.1128/JVI.01052-08 Medline

66. M. Lu, P. D. Uchil, W. Li, D. Zheng, D. S. Terry, J. Gorman, W. Shi, B. Zhang, T. Zhou, S. Ding, R. Gasser, J. Prévost, G. Beaudoin-Bussières, S. P. Anand, A. Laumaea, J. R. Grover, L. Liu, D. D. Ho, J. R. Mascola, A. Finzi, P. D. Kwong, S. C. Blanchard, W. Mothes, Real-Time Conformational Dynamics of SARS-CoV-2 Spikes on Virus Particles. *Cell Host Microbe* **28**, 880–891.e8 (2020). doi:10.1016/j.chom.2020.11.001 Medline

67. S. Ramirez, C. Fernandez-Antunez, A. Galli, A. Underwood, L. V. Pham, L. A. Ryberg, S. Feng, M. S. Pedersen, L. S. Mikkelsen, S. Belouzard, J. Dubuisson, C. Sølund, N. Weis, J. M. Gottwein, U. Fahnøe, J. Bukh, Overcoming culture restriction for SARS-CoV-2 in human cells facilitates the screening of compounds inhibiting viral replication. *Antimicrob. Agents Chemother.* **65**, e0009721 (2021). doi:10.1128/AAC.00097-21 Medline

68. Z. Liu, H. Zheng, H. Lin, M. Li, R. Yuan, J. Peng, Q. Xiong, J. Sun, B. Li, J. Wu, L. Yi, X. Peng, H. Zhang, W. Zhang, R. J. G. Hulswit, N. Loman, A. Rambaut, C. Ke, T. A. Bowden, O. G. Pybus, J. Lu, Identification of Common Deletions in the Spike Protein of Severe Acute Respiratory Syndrome Coronavirus 2. *J. Virol.* **94**, e00790-20 (2020). doi:10.1128/JVI.00790-20 Medline

69. R. Sanchez-Velazquez, G. de Lorenzo, R. Tandavanitj, C. Setthapramote, P. J. Bredenbeek, L. Bozzacco, M. R. MacDonald, J. J. Clark, C. M. Rice, A. H. Patel, A. Kohl, M. Varjak, Generation of a reporter yellow fever virus for high throughput antiviral assays. *Antiviral Res.* **183**, 104939 (2020). doi:10.1016/j.antiviral.2020.104939 Medline

70. T. T. N. Thao, F. Labroussaa, N. Ebert, J. Jores, V. Thiel, In-Yeast Assembly of Coronavirus Infectious cDNA Clones Using a Synthetic Genomics Pipeline. *Methods Mol. Biol.* **2203**, 167–184 (2020). doi:10.1007/978-1-0716-0900-2_13 Medline

71. H.-H. Hoffmann, W. M. Schneider, K. Rozen-Gagnon, L. A. Miles, F. Schuster, B. Razooky, E. Jacobson, X. Wu, S. Yi, C. M. Rudin, M. R. MacDonald, L. K. McMullan, J. T. Poirier, C. M. Rice, TMEM41B Is a Pan-flavivirus Host Factor. *Cell* **184**, 133–148.e20 (2021). doi:10.1016/j.cell.2020.12.005 Medline

72. B. D. Lindenbach, M. J. Evans, A. J. Syder, B. Wölk, T. L. Tellinghuisen, C. C. Liu, T. Maruyama, R. O. Hynes, D. R. Burton, J. A. McKeating, C. M. Rice, Complete replication of hepatitis C virus in cell culture. *Science* **309**, 623–626 (2005). doi:10.1126/science.1114016 Medline

73. D. K. W. Chu, Y. Pan, S. M. S. Cheng, K. P. Y. Hui, P. Krishnan, Y. Liu, D. Y. M. Ng, C. K. C. Wan, P. Yang, Q. Wang, M. Peiris, L. L. M. Poon, Molecular Diagnosis of a Novel Coronavirus (2019-nCoV) Causing an Outbreak of Pneumonia. *Clin. Chem.* **66**, 549–555 (2020). doi:10.1093/clinchem/hvaa029 Medline
