# Supplementary material for: Replication and single-cycle delivery of SARS-CoV-2 replicons
Source: Science. 2021 Oct 14;374(6571):1099–106. doi: 10.1126/science.abj8430 (PMC9007107; doi:10.1126/science.abj8430)
Supplement: Supplementary file 3 — Table S1 [file science.abj8430_table_s1.zip › science.abj8430_table_s1_caption.pdf]

Table S1. Primers used in this study. List of primers for SARS-CoV-2 replicon construction organized by PCR reaction. Final fragments for subcloning and yeast transformation are highlighted in green, all others are intermediate overlap PCR templates. Primers in blue are from (*10*).
